# Design, microwave-assisted synthesis, bioactivity and SAR of novel substituted 2-phenyl-2-cyclohexanedione enol ester derivatives†

**DOI:** 10.1039/c8ra02647e

**Published:** 2018-05-29

**Authors:** Fei Ye, Peng Ma, Yue Zhai, Fei Yang, Shuang Gao, Li-Xia Zhao, Ying Fu

**Affiliations:** Department of Applied Chemistry, College of Science, Northeast Agricultural University Harbin 150030 P. R. China fuying@neau.edu.cn

## Abstract

Based on the structure–activity relationship and active substructure combination, a novel class of substituted 2-phenyl-2-cyclohexanedione enol ester derivatives was designed for use as potential herbicide safeners. A microwave-assisted synthetic route was developed for the substituted 2-phenyl-2-cyclohexenone enol ester derivatives *via* coupling and acylation reactions. In the modified protocol, the reactions were performed under microwave irradiation, resulting in significant improvements in the yields and reaction times. All of the structures were characterized using IR, ^1^H NMR, ^13^C NMR and HRMS spectroscopies. The bioassay results demonstrated that most of these compounds could alleviate clethodim injury to maize. Molecular docking modeling showed that the potential antagonism between compound 3(S24) and clethodim plays a key role in the metabolism of herbicides. This paper presents a new safener candidate for maize protection.

## Abstract 2

Based on the structure–activity relationship and active substructure combination, a novel class of substituted 2-phenyl-2-cyclohexanedione enol ester derivatives was designed for use as potential herbicide safeners.

## Graphical abstract

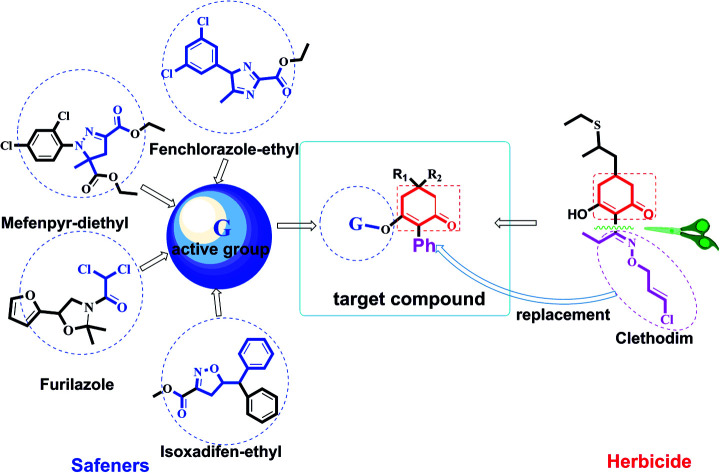

## Introduction

Clethodim, an acetyl CoA carboxylase (ACCase) inhibitor that belongs to the chemical family of cyclohexenone herbicides, specifically targets the carboxylase-transferase (CT) domain of the plastidic ACCase, which inhibits fatty acid biosynthesis and ultimately causes crop death.^1,2^ Cyclohexenone herbicides are toxic to non-target organisms that also contain this enzyme.^3,4^ Clethodim has been shown to cause albinism in canola *via* the inhibition of fatty acid biosynthesis, which not only causes dramatic crop yield losses but also die-off. As a consequence, the continuous development of new safeners to protect crops is urgently required to expand the application and increase the safety of herbicides.

It is widely accepted that broad-spectrum herbicides can be combined with a safener for crop protection and efficient weed management. The safener induces the degradation of the herbicides only in the crops, not in the weeds.^5^ This effect is connected with the increased expression of gene coding for the enzymes responsible for herbicide degradation in crops, such as the cytochrome P450 monooxygenases (CYP), glutathione S-transferases (GST) and ATP-binding cassette (ABC) transporters.^6–9^ For example, isoxadifen-ethyl exhibited safening activity to protect corn against injury caused by the herbicide foramsulfuron by inducing CYP or glycosyl transferase activity.^10–12^ Several studies have reported that the safener isoxadifen-ethyl enhances GST activity and herbicide tolerance.^13,14^ A new era in safener research began with the discovery of 1,2,4-triazolcarboxylates and fenchlorazole-ethyl was developed as a post-emergence safener for wheat against the ACCase inhibitor fenoxaprop-ethyl.^15^ Similarly, the dihydropyrazol dicarboxylate mefenpyr-diethyl has been used against ACCase inhibitors, including fenoxaprop herbicides.^16^

The structure–activity relationship (SAR), an important measure for novel agrochemical discovery, has been introduced into many research works focused on the search for bioactive compounds.^17^ Stephenson *et al.* showed that compounds that share structural similarities to the thiocarbamate herbicides were found to be highly active antidotes for those herbicides in corn.^18^ Sulfamide compounds have been used as safeners to protect plants from the injury caused by sulfonylurea herbicides. Cyprosulfamide, designed based on the SAR, could protect plants from the injury caused by thiencarbazone-methyl.^19^ Safeners, having structural features closely resembling those of herbicides, may interfere in the metabolism of herbicides that protect crops.^20^

**Scheme 1:**
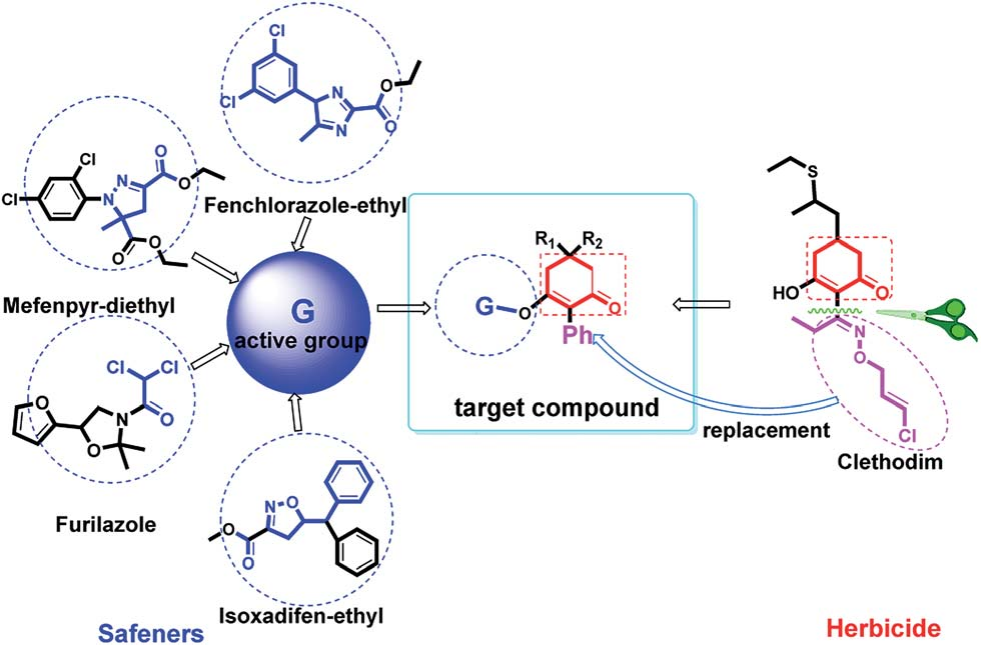
Design of the target compound.

In recent years, the active substructure combination has become an effective method for improving the hit of compounds. The safener benzhydryloxy-acetic acid combined with the potential safener 5-phenyl-4,5-dihydroisoxazole-3-ethyl ester led to the production of the strong rice safener isoxadifen-ethyl.^21^ A class of new isoquinolinium-like compounds and novel chiral succinate dehydrogenase inhibitors was also designed by utilizing the active substructure combination theory, and they exhibited excellent and broad spectrum antifungal activity.^22,23^ In connection with the facts mentioned above and our earlier work on the design and synthesis of N-containing heterocyclic safeners,^24–26^ herein substituted 2-phenyl-2-cyclohexanedione enol ester derivatives were designed based on the SAR and active substructure combination (Scheme 1). Greenhouse experiments demonstrated that some of them exhibited promising safener activity for protecting crops from injury by clethodim.

## Experimental

### Chemicals and instruments

Infrared (IR) spectra were recorded on FTIR-8400S and ALPHA-T infrared spectrophotometers as KBr pellets. The nuclear magnetic resonance (NMR) spectra were recorded on Bruker AVANVE 400 MHz and Bruker AVANVE 600 MHz spectrometers, using CDCl_3_ as the solvent and TMS as the internal standard. The melting points were determined on a Beijing Taike melting point apparatus (X-4) and were uncorrected. The mass spectra were obtained by a Xevo TQ spectrometer. Microwave activation was carried out using an XH-100A focused microwave (2450 MHz, 1000 W, 40 L, normal pressure, Beijing Xiang Hu Sci. and Tech., Beijing, NC, China). All reagents used were of analytical grade (purchased from Aladdin Industrial Inc., Shanghai, China).

### General procedure for the preparation of substituted 3-hydroxy-2-phenylcyclohex-2-en-1-one derivatives, 2

To dimethylsulfoxide (DMSO, 50 ml) kept at 90 °C under microwave irradiation (800 W), iodobenzene (1.02 g, 5 mmol), l-proline (0.12 g, 1 mmol) and CuI (0.10 g, 0.5 mmol) were added. Substituted 1,3-cyclohexanedione 1 (15 mmol) was added *via* syringe. After 40 min, the organic phase was dried over anhydrous Na_2_SO_4_ and the solvent was removed by vacuum distillation. The pure products were obtained by column chromatography on silica gel eluted with petroleum ether and ethyl acetate (3 : 1).

#### 3-Hydroxy-2-phenylcyclohex-2-en-1-one (2a)

White solid; yield 79%; mp 150–151 °C; ^1^H NMR (400 MHz, CDCl_3_): 7.48–7.20 (m, 5H, Ar–H), 2.60–2.55 (t, *J* = 6.3 Hz, 4H, CH_2_), 2.15–2.06 (m, 2H, CH_2_); ^13^C NMR (100 MHz, CDCl_3_): 196.8, 170.7, 130.9, 130.7 (2C), 129.4 (2C), 128.3, 118.2, 37.0, 28.0, 20.5; IR (KBr, cm^−1^): 3035–2865 (C–H), 1591 (C=O), 1554 (C=C), 1176 (C–O).

#### 3-Hydroxy-5-methyl-2-phenylcyclohex-2-en-1-one (2b)

White solid; yield 84%; mp 167–168 °C; ^1^H NMR (400 MHz, CDCl_3_): 7.49–7.21 (m, 5H, Ar–H), 6.06 (s, 1H, O–H), 2.64–2.60 (d, *J* = 15.7 Hz, 2H, CH_2_), 2.40–2.36 (m, 2H, CH_2_), 1.60 (s, 1H,CH), 1.18–1.17 (d, *J* = 6.0 Hz, 3H, CH_3_); ^13^C NMR (100 MHz, CDCl_3_): 130.8, 130.6 (2C), 129.4 (2C), 128.3, 117.7, 45.3, 28.1, 21.1; IR (KBr, cm^−1^): 3027–2846 (C–H), 1592 (C=O), 1557 (C=C), 1137 (C–O).

#### 3-Hydroxy-5,5-dimethyl-2-phenylcyclohex-2-en-1-one (2c)

White solid; yield 88%; mp 161–162 °C; ^1^H NMR (400 MHz, CDCl_3_): 7.47–7.21 (m, 5H, Ar–H), 6.02 (s, 1H, O–H), 2.45 (s, 4H, CH_2_), 1.18 (s, 6H, CH_3_); ^13^C NMR (100 MHz, CDCl_3_): 130.7, 130.6 (2C), 129.4 (2C), 128.3, 116.9, 31.8, 28.4 (2C); IR (KBr, cm^−1^): 3028–2852 (C–H), 1606 (C=O), 1581 (C=C), 1133 (C–O).

### General procedure for the preparation of substituted 2-phenyl-2-cyclohexanedione enol ester derivatives, 3

To CH_2_Cl_2_ (20 ml) maintained at 0 °C, intermediate 2(a–c) and Et_3_N (1.5 mmol) were added. After five minutes of stirring, acyl chloride (1.2 mmol) was added *via* syringe. The solution was warmed to room temperature over 1 hour. The reaction mixture was extracted with CH_2_Cl_2_ and filtered. The organic layer was dried over anhydrous Na_2_SO_4_ and the CH_2_Cl_2_ was evaporated under vacuum. The pure products were obtained by recrystallization or column chromatography on silica gel eluting with petroleum ether and ethyl acetate (3 : 1–9 : 1).

#### 3-Benzoyloxy-2-phenyl-2-cyclohexen-1-one 3(S1)

White solid; yield 50%; mp 75.7–77.2 °C; spectroscopic data consistent with those observed previously.^27^

#### 3-Benzoyloxy-2-phenyl-5-methyl-2-cyclohexen-1-one 3(S2)

White solid; yield 56%; mp 80.2–81.0 °C; ^1^H NMR (600 MHz, CDCl_3_): 7.22–7.88 (m, 10H, Ar–H); 2.76–2.86 (m, 2H, CH_2_); 2.69–2.73 (m, 1H, CH_2_); 2.54–2.55 (m, 1H, CH); 2.38–2.45 (m, 1H, CH_2_); 1.21–1.23 (d, 3H, *J* = 6.4 Hz, CH_3_); ^13^C NMR (150 MHz, CDCl_3_): 197.9, 164.8, 163.6, 133.9, 131.3, 130.1 (2C), 130.0, 129.7 (2C), 128.6 (3C), 127.9 (2C), 127.7, 45.9, 37.3, 28.6, 20.9; IR (KBr, cm^−1^): 3036, 2851 (C–H), 1721, 1665 (C=O), 1587 (C=C), 1234 (C–O); HRMS (ESI): *m*/*z* [M + Na^+^] calcd for monoisotopic 329.1256, found 329.1148.

#### 3-Benzoyloxy-2-phenyl-5,5-dimethyl-2-cyclohexen-1-one 3(S3)

White solid; yield 65%; mp 84.3–86.2 °C; ^1^H NMR (400 MHz, CDCl_3_): 7.19–7.88 (m, 10H, Ar–H); 2.77 (s, 2H, CH_2_); 2.56 (s, 2H, CH_2_); 1.26 (s, 6H, CH_3_); ^13^C NMR (100 MHz, CDCl_3_): 197.7, 163.7, 163.6, 133.8, 131.2, 130.0 (2C), 129.6 (2C), 129.5, 128.6, 128.6 (2C), 127.9 (2C), 127.7, 51.6, 43.0, 32.7, 28.2 (2C); IR (KBr, cm^−1^): 3065, 2872 (C–H), 1732, 1677 (C=O), 1599 (C=C), 1257 (C–O); HRMS (ESI): *m*/*z* [M + H^+^] calcd for monoisotopic mass 321.1412, found 321.1485.

#### 3-(2,4-Dichlorobenzoyloxy)-2-phenyl-2-cyclohexen-1-one 3(S4)

White solid; yield 59%; mp 103.9–104.9 °C; ^1^H NMR (600 MHz, CDCl_3_): 7.17–7.45 (m, 8H, Ar–H); 2.86–2.89 (t, 2H, *J* = 9.3 Hz, CH_2_); 2.67–2.70 (t, 2H, *J* = 10.2 Hz, CH_2_); 2.24–2.27 (t, 2H, *J* = 9.9 Hz, CH_2_); ^13^C NMR (150 MHz, CDCl_3_): 197.9, 164.9, 161.3, 139.3, 135.4, 132.6, 131.3, 131.2, 131.0, 129.6 (2C), 128.1 (2C), 128.0, 127.1, 126.5, 37.6, 29.0, 20.8; IR (KBr, cm^−1^): 3059, 2850 (C–H), 1734, 1667 (C=O), 1572 (C=C), 1256 (C–O); HRMS (ESI): *m*/*z* [M + Na^+^] calcd for monoisotopic mass 383.0320, found 383.0212.

#### 3-(2,4-Dichlorobenzoyloxy)-2-phenyl-5-methyl-2-cyclohexen-1-one 3(S5)

White solid; yield 62%; mp 73.3–74.3 °C; ^1^H NMR (600 MHz, CDCl_3_): 7.16–7.46 (m, 8H, Ar–H); 2.80–2.85 (m, 1H, CH_2_); 2.67–2.77 (m, 2H, CH_2_); 2.51–2.60 (m, 1H, CH); 2.37–2.44 (m, 1H, CH_2_); 1.22–1.24 (d, 3H, CH_3_); ^13^C NMR (150 MHz, CDCl_3_): 197.8, 164.2, 161.3, 139.3, 135.5, 133.4, 132.6, 131.2, 130.5, 129.6 (2C), 128.1 (2C), 128.0, 127.1, 126.5, 45.8, 37.1, 28.6, 20.9; IR (KBr, cm^−1^): 3060, 2851 (C–H), 1734, 1667 (C=O), 1571 (C=C), 1256 (C–O); HRMS (ESI): *m*/*z* [M + Na^+^] calcd for monoisotopic mass 397.0476, found 397.0369.

#### 3-(2,4-Dichlorobenzoyloxy)-2-phenyl-5,5-dimethyl-2-cyclohexen-1-one 3(S6)

Colourless oil; yield 70%; ^1^H NMR (600 MHz, CDCl_3_) δ: 7.17–7.46 (m, 8H, Ar–H), 2.76 (s, 2H, CH_2_), 2.56 (m, 2H, CH_2_), 1.27 (s, 6H, CH_3_); ^13^C NMR (150 MHz, CDCl_3_): 197.6, 163.1, 161.4, 139.3, 135.4, 132.6, 131.2, 131.1, 129.9 (2C), 129.5, 128.1 (2C), 128.0, 127.1, 126.5, 51.5, 42.7, 32.7, 28.2 (2C). IR (KBr, cm^−1^): 3060, 2849 (C–H), 1735, 1668 (C=O), 1571 (C=C), 1256 (C–O); HRMS (ESI): *m*/*z* [M + Na^+^] calcd for monoisotopic mass 411.0633, found 411.0525.

#### 3-Chloroacetyloxy-2-phenyl-5-methyl-2-cyclohexen-1-one 3(S7)

Yellow oil; yield 33%; ^1^H NMR (400 MHz, CDCl_3_): 7.10–7.37 (m, 5H, Ar–H); 3.91 (s, 2H, CH_2_–Cl); 2.73–2.77 (t, 2H, *J* = 8.2 Hz, CH_2_); 2.62–2.66 (t, 2H, *J* = 9.0 Hz, CH_2_); 2.16–2.24 (m, 2H, CH_2_); ^13^C NMR (100 MHz, CDCl_3_): 197.5, 164.2, 164.1, 130.8, 129.5 (2C), 128.1 (2C), 128.1 (2C), 40.3, 37.4, 28.7, 20.6; IR (KBr, cm^−1^): 3064, 2872 (C–H), 1776, 1680 (C=O), 1598 (C=C), 1232 (C–O); HRMS (ESI): *m*/*z* [M + H^+^] calcd for monoisotopic mass 265.7042, found 265.0626.

#### 3-Chloroacetyloxy-2-phenyl-5-methyl-2-cyclohexen-1-one 3(S8)

Yellow oil; yield 35%; ^1^H NMR (600 MHz, CDCl_3_): 7.10–7.39 (m, 5H, Ar–H); 3.918 (s, 2H, CH_2_–Cl); 2.66–2.73 (m, 2H, CH_2_); 2.54–2.61 (m, 1H, CH_2_); 2.48–2.51 (m, 1H, CH); 2.33–2.39 (m, 1H, CH_2_); 1.19–1.20 (d, 3H, *J* = 9.7 Hz, CH_3_); ^13^C NMR (150 MHz, CDCl_3_): 197.6, 164.2, 163.6, 130.8, 129.5 (2C), 128.1 (2C), 128.1, 127.2, 45.7, 40.3, 36.7, 28.4, 20.8; IR (KBr, cm^−1^): 3060, 2872 (C–H), 1778, 1674 (C=O), 1645 (C=C), 1126 (C–O); HRMS (ESI): *m*/*z* [M + Na^+^] calcd for monoisotopic mass 301.0710, found 301.0602.

#### 3-Chloroacetyloxy-2-phenyl-5,5-dimethyl-2-cyclohexen-1-one 3(S9)

Yellow oil; yield 40%; ^1^H NMR (400 MHz, CDCl_3_): 7.11–7.39 (m, 5H, Ar–H); 3.91 (s, 2H, CH_2_–Cl); 2.63 (s, 2H, CH_2_); 2.51 (s, 2H, CH_2_); 1.22 (s, 6H, –(CH_3_)_2_); ^13^C NMR (CDCl_3_): 197.3, 164.3, 162.4, 130.7, 129.7, 129.4 (2C), 128.1 (2C), 128.0, 51.4, 42.4, 40.3, 32.6 (2C), 28.1; IR (KBr, cm^−1^): 3058, 2872 (C–H), 1776, 1680 (C=O), 1598 (C=C), 1141 (C–O); HRMS (ESI): *m*/*z* [M + H^+^] calcd for monoisotopic mass 293.0866, found 293.0939.

#### 3-Dichloroacetyloxy-2-phenyl-5,5-dimethyl-2-cyclohexen-1-one 3(S10)

White solid; yield 48%; mp 79.6–81.3 °C; ^1^H NMR (400 MHz, CDCl_3_): 7.11–7.40 (m, 5H, Ar–H); 5.80 (s, 1H, CH–Cl_2_); 2.64 (s, 2H, CH_2_); 2.53 (s, 2H, CH_2_); 1.24 (s, 6H, –(CH_3_)_2_); ^13^C NMR (100 MHz, CDCl_3_): 197.1, 161.7, 161.1, 130.3, 130.0, 129.5 (2C), 128.2, 128.1 (2C), 63.5, 51.4, 41.8, 32.6, 28.2 (2C); IR (KBr, cm^−1^): 3060, 2872 (C–H), 1754, 1659 (C=O), 1576 (C=C), 1257 (C–O); HRMS (ESI): *m*/*z* [M + H^+^] calcd for monoisotopic mass 327.0476, found 327.0549.

#### 3-Phenoxyacetyloxy-2-phenyl-2-cyclohexen-1-one 3(S11)

White solid, yield 50%; mp 80.1–81.7 °C; ^1^H NMR (400 MHz, CDCl_3_): 6.62–7.42 (m, 10H, Ar–H), 4.50 (s, 2H, O=C–CH_2_–O), 2.73–2.77 (t, 2H, *J* = 6.2 Hz, CH_2_), 2.63–2.66 (t, 2H, *J* = 6.9 Hz, CH_2_), 2.17–2.24 (m, 2H, CH_2_); ^13^C NMR (100 MHz, CDCl_3_): 197.5, 165.9, 164.2, 157.3, 131.1, 131.0, 129.7 (2C), 129.6 (2C), 128.1 (2C), 128.0, 122.0, 114.4 (2C), 64.8, 37.5, 28.9, 20.6; IR (KBr, cm^−1^): 3034–2852 (C–H), 1767, 1653 (C=O), 1586 (C=C), 1124 (C–O); HRMS (ESI): *m*/*z* [M + Na^+^] calcd for monoisotopic mass 345.1405, found 345.1097.

#### 3-Phenoxyacetyloxy-2-phenyl-5-methyl-2-cyclohexen-1-one 3(S12)

Colourless oil; yield 57%; ^1^H NMR (600 MHz, CDCl_3_): 6.62–7.42 (m, 10H, Ar–H), 4.51 (s, 2H, O=C–CH_2_–O), 2.71–2.73 (m, 1H, CH_2_), 2.67–2.69 (m, 1H, CH_2_), 2.53–2.60 (m, 1H, CH_2_), 2.47–2.51 (m, 1H, CH), 2.33–2.40 (m, 1H, CH_2_), 1.19–1.20 (d, 3H, *J* = 9.6 Hz, CH_3_). ^13^C NMR (150 MHz, CDCl_3_): 197.5, 166.0, 163.6, 157.4, 131.0, 130.5, 129.7 (2C), 129.6 (2C), 128.1 (2C), 128.0, 122.0, 114.4 (2C), 64.8, 45.7, 36.9, 28.3, 20.8; IR (KBr, cm^−1^): 3035–2850 (C–H), 1770, 1665 (C=O), 1586(C=C), 1132 (C–O); HRMS (ESI): *m*/*z* [M + Na^+^] calcd for monoisotopic mass 359.1362, found 359.1254.

#### 3-Phenoxyacetyloxy-2-phenyl-5,5-dimethyl-2-cyclohexen-1-one 3(S13)

White solid; yield 64%; mp 84.7–86.7 °C; ^1^H NMR (600 MHz, CDCl_3_): 6.61–7.41 (m, 10H, Ar–H), 4.51 (s, 2H, O=C–CH_2_–O), 2.63 (s, 2H, CH_2_), 2.52 (s, 2H, CH_2_), 1.22 (s, 6H, –(CH_3_)_2_). ^13^C NMR (150 MHz, CDCl_3_): 197.4, 166.1, 162.6, 157.3, 130.9, 129.9, 129.6 (4C), 128.2 (2C), 128.0, 122.0, 114.4 (2C), 64.7, 51.4, 42.7, 32.6, 28.2 (2C); IR (KBr, cm^−1^) *ν*: 3033–2843 (C–H), 1758, 1666 (C=O), 1585 (C=C), 1137 (C–O); HRMS (ESI): *m*/*z* [M + H^+^] calcd for monoisotopic mass 351.1518, found 351.1519.

#### 3-(3-Acetylpropionoxy)-2-phenyl-2-cyclohexen-1-one 3(S14)

Colourless oil; yield 39%; ^1^H NMR (400 MHz, CDCl_3_): 7.09–7.40 (m, 5H, Ar–H), 4.45 (s, 2H, O=C–CH_2_–O), 2.74–2.78 (t, 2H, *J* = 8.2 Hz, CH_2_), 2.60–2.65 (t, 2H, *J* = 8.9 Hz, CH_2_), 2.14–2.23 (m, 2H, CH_2_), 2.12 (s, 3H, O=C–CH_3_); ^13^C NMR (100 MHz, CDCl_3_): 197.5, 170.0, 164.9, 164.1, 130.9, 130.6, 129.5 (2C), 128.0 (2C), 127.9, 60.2, 37.5, 28.8, 20.6, 20.2; IR (KBr, cm^−1^): 3058–2893 (C–H), 1755, 1680 (C=O), 1599 (C=C), 1150 (C–O); HRMS (ESI): *m*/*z* [M + Na^+^] calcd for monoisotopic mass 311.0998, found 311.0890. 3(S14).

#### 3-(3-Acetylpropionoxy)-2-phenyl-5-methyl-2-cyclohexen-1-one 3(S15)

Colourless oil; yield 43%; ^1^H NMR (600 MHz, CDCl_3_): 7.10–7.40 (m, 5H, Ar–H), 4.46 (s, 2H, O=C–CH_2_–O), 2.71–2.73 (m, 1H, CH_2_), 2.67–2.69 (m, 1H, CH_2_), 2.55–2.62 (m, 1H, CH_2_), 2.46–2.51 (m, 1H, CH), 2.31–2.38 (m, 1H, CH_2_), 2.13 (s, 3H, O=C–CH_3_), 1.18–1.20 (d, 3H, *J* = 9.7 Hz, CH_3_); ^13^C NMR (150 MHz, CDCl_3_): 197.6, 170.1, 165.0, 163.6, 130.8, 130.2, 129.5 (2C), 128.1 (2C), 128.0, 60.2, 45.7, 36.8, 28.4, 20.8, 20.3; IR (KBr, cm^−1^): 3034–2851 (C–H), 1742, 1667 (C=O), 1143 (C–O); HRMS (ESI): *m*/*z* [M + Na^+^] calcd for monoisotopic mass 325.1154, found 325.1046.

#### 3-(3-Acetylpropionoxy)-2-phenyl-5,5-dimethyl-2-cyclohexen-1-one 3(S16)

Yellow oil; yield 47%; ^1^H NMR (400 MHz, CDCl_3_): 7.09–7.39 (m, 5H, Ar–H), 4.44 (s, 2H, O=C–CH_2_–O), 2.62 (s, 2H, CH_2_), 2.49 (s, 2H, CH_2_), 2.11 (s, 3H, O=C–CH_3_), 1.20 (s, 6H, –(CH_3_)_2_); ^13^C NMR (100 MHz, CDCl_3_): 197.5, 170.1, 165.1, 162.5, 130.8, 129.6, 129.5 (2C), 128.1 (2C), 128.0, 60.2, 51.4, 42.5, 32.6, 28.1 (2C), 20.3; IR (KBr, cm^−1^): 3058–2872 (C–H), 1756, 1680 (C=O), 1587 (C=C), 1145 (C–O); HRMS (ESI): *m*/*z* [M + Na^+^] calcd for monoisotopic mass 339.1311, found 339.1203.

#### 3-[1-(2,4-Dichlorophenyl)-5-(trichloromethyl)-1*H*-1,2,4-triazol-3-yl]carbonyloxy-2-phenyl-2-cyclohexen-1-one 3(S17)

White solid; yield 46%; mp 172.4–173.1 °C; ^1^H NMR (600 MHz, CDCl_3_): 7.22–7.61 (m, 8H, Ar–H), 2.88–2.92 (m, 2H, CH_2_), 2.67–2.71 (t, 2H, *J* = 9.9 Hz, CH_2_), 2.22–2.28 (m, 2H, CH_2_); ^13^C NMR (150 MHz, CDCl_3_): 197.5, 163.9, 155.9, 155.2, 151.1, 138.5, 134.0, 133.2 (2C), 130.7 (2C), 130.6, 129.8 (2C), 128.0 (2C), 127.9, 127.8, 98.0, 37.6, 28.9, 20.7; IR (KBr, cm^−1^): 3083–2857 (C–H), 1746, 1684 (C=O), 1647 (C=C), 1173 (C–O); HRMS (ESI): *m*/*z* [M + H^+^] calcd for monoisotopic mass 543.9478 found, 543.9551.

#### 3-[1-(2,4-Dichlorophenyl)-5-(trichloromethyl)-1*H*-1,2,4-triazol-3-yl]carbonyloxy-2-phenyl-5-methyl-2-cyclohexen-1-one 3(S18)

White solid; yield 54%; mp 193.9–195.1 °C; ^1^H NMR (600 MHz, CDCl_3_): 7.22–7.61 (m, 8H, Ar–H), 2.80–2.88 (m, 1H, CH_2_), 2.66–2.76 (m, 2H, CH_2_), 2.52–2.57 (m, 1H, CH), 2.38–2.45 (m, 1H, CH_2_), 1.21–1.23 (d, 3H, *J* = 10.2 Hz, CH_3_); ^13^C NMR (150 MHz, CDCl_3_): 197.5, 163.2, 155.9, 155.2, 151.5, 138.4, 134.0, 133.2, 130.6 (2C), 130.6 (2C), 129.7 (2C), 128.0 (2C), 127.9, 127.7, 84.8, 45.8, 36.9, 28.5, 20.9; IR (KBr, cm^−1^): 3133–2876 (C–H), 1748, 1677 (C=O), 1542 (C=C), 1171 (C–O); HRMS (ESI): *m*/*z* [M + H^+^] calcd for monoisotopic mass 557.9634, found 557.9707.

#### 3-[1-(2,4-Dichlorophenyl)-5-(trichloromethyl)-1*H*-1,2,4-triazol-3-yl]carbonyloxy-2-phenyl-5,5-dimethyl-2-cyclohexen-1-one 3(S19)

White solid; yield 80%; mp 180.8–182.0 °C; ^1^H NMR (400 MHz, CDCl_3_): 7.22–7.60 (m, 8H, Ar–H), 2.75–2.77 (d, 2H, *J* = 6.4 Hz, CH_2_), 2.56 (s, 2H, CH_2_), 1.24–1.25 (d, 6H, *J* = 2.4 Hz, CH_2_); ^13^C NMR (100 MHz, CDCl_3_): 197.5, 162.2, 156.0, 155.3, 151.6, 138.5, 134.1, 133.2, 130.7, 130.6 (2C), 130.1, 129.8 (2C), 128.0 (2C), 127.9, 127.8, 84.9, 51.6, 42.6, 32.7 (2C), 28.4; IR (KBr, cm^−1^): 3084–2874 (C–H), 1749, 1682 (C=O), 1639 (C=C), 1185 (C–O); HRMS (ESI): *m*/*z* [M + H^+^] calcd for monoisotopic mass 571.9791, found 571.9864.

#### 3-[5-Methyl-3-phenylisoxazole-4-carbonyloxy]-2-phenyl-2-cyclohexen-1-one 3(S20)

White solid; yield 79%; mp 99.3–101.1 °C; ^1^H NMR (400 MHz, CDCl_3_): 7.03–7.47 (m, 10H, Ar–H), 2.73–2.77 (t, 2H, *J* = 8.4 Hz, CH_2_), 2.59–2.63 (t, 2H, *J* = 9.0 Hz, CH_2_), 2.45 (s, 3H, C=C–CH_3_), 2.06–2.22 (m, 2H, CH_2_); ^13^C NMR (100 MHz, CDCl_3_): 197.5, 177.3, 164.3, 162.5, 158.6, 131.5, 130.9, 129.9, 129.5 (2C), 129.2 (2C), 128.1 (2C), 128.1 (2C), 128.0, 127.7, 107.0, 37.4, 29.0, 20.7, 13.5; IR (KBr, cm^−1^): 3058–2871 (C–H), 1739, 1679 (C=O), 1595 (C=C), 1142 (C–O); HRMS (ESI): *m*/*z* [M + H^+^] calcd for monoisotopic mass 374.1314, found 374.1387.

#### 3-[5-Methyl-3-phenylisoxazole-4-carbonyloxy]-2-phenyl-5-methyl-2-cyclohexen-1-one 3(S21)

Colourless oil; yield 83%; ^1^H NMR (600 MHz, CDCl_3_): 7.03–7.50 (m, 10H, Ar–H), 2.66–2.73 (m, 2H, CH_2_), 2.55–2.62 (m, 1H, CH_2_), 2.46 (s, 3H, C=C–CH_3_), 2.30–2.37 (m, 1H, CH_2_), 1.67 (s, 1H, CH), 1.17–1.19 (d, 3H, *J* = 9.6 Hz, CH_3_). ^13^C NMR (150 MHz, CDCl_3_): 197.6, 177.4, 163.8, 162,5, 158.7, 131.4, 130.4, 130.0, 129.5 (2C), 129.2 (2C), 128.2 (2C), 128.1 (2C), 128.0, 127.7, 107.0, 45.7, 37.2, 28.5, 20.9, 13.5; IR (KBr, cm^−1^): 3058–2874 (C–H), 1739, 1681 (C=O), 1595 (C=C), 1065 (C–O); HRMS (ESI): *m*/*z* [M + H^+^] calcd for monoisotopic mass 388.1471, found 388.1543.

#### 3-[5-Methyl-3-phenylisoxazole-4-carbonyloxy]-2-phenyl-5,5-dimethyl-2-cyclohexen-1-one 3(S22)

White solid; yield 91%; mp 105.2–106.9 °C; ^1^H NMR (600 MHz, CDCl_3_): 7.02–7.50 (m, 10H, Ar–H), 2.64 (s, 2H, CH_2_), 2.49 (s, 2H, CH_2_), 2.47 (s, 3H, C=C–CH_3_), 1.21 (s, 6H, –(CH_3_)_2_); ^13^C NMR (150 MHz, CDCl_3_): 197.5, 177.4, 162.6, 162.5, 158.7, 131.3, 130.0, 129.7, 129.5 (2C), 129.2 (2C), 128.2 (2C), 128.1 (2C), 128.0, 127.7, 107.1, 51.4, 42.8, 32.7, 28.2 (2C), 13.5; IR (KBr, cm^−1^): 3065–2870 (C–H), 1723, 1657 (C=O), 1597 (C=C), 1064 (C–O); HRMS (ESI): *m*/*z* [M + H^+^] calcd for monoisotopic mass 402.1627, found 402.1700.

#### 3-[5-Methyl-3-(2-fluoro-6-chlorophenyl)isoxazole-4-carbonyloxy]-2-phenyl-2-cyclohexen-1-one 3(S23)

Colourless liquid; yield 59%; ^1^H NMR (600 MHz, CDCl_3_): 6.90–7.45 (m, 8H, Ar–H), 2.72–2.75 (t, 2H, *J* = 9.0 Hz, CH_2_), 2.58–2.61 (t, 2H, *J* = 9.9 Hz, CH_2_), 2.51 (s, 3H, C=C–CH_3_), 2.12–2.19 (m, 2H, CH_2_); ^13^C NMR (150 MHz, CDCl_3_): 197.6, 177.1 (2C), 161.9, 159.4, 157.7, 155.3, 135.1, 131.9, 131.0, 129.3 (2C), 128.0 (2C), 127.9, 125.3, 116.9, 114.3, 108.5, 37.5, 28.8, 20.7, 13.2; IR (KBr, cm^−1^): 3059–2850 (C–H), 1726, 1666 (C=O), 1587 (C=C), 1071 (C–O); HRMS (ESI): *m*/*z* [M + H^+^] calcd for monoisotopic mass 426.0830 found 426.0903.

#### 3-[5-Methyl-3-(2-fluoro-6-chlorophenyl)isoxazole-4-carbonyloxy]-2-phenyl-5-methyl-2-cyclohexen-1-one 3(S24)

White solid; yield 70%; mp 143.3–144.2 °C; ^1^H NMR (600 MHz, CDCl_3_): 6.88–7.45 (m, 8H, Ar–H), 2.64–2.71 (m, 2H, CH_2_), 2.54–2.59 (t, 1H, *J* = 13.5 Hz, CH_2_), 2.52 (s, 3H, C=C–CH_3_), 2.42–2.46 (m, 1H, CH), 2.27–2.34 (m, 1H, CH_2_), 1.16–1.17 (d, 3H, *J* = 4.8 Hz, –CH_3_); ^13^C NMR (150 MHz, CDCl_3_): 197.5, 177.2, 163.4, 159.4, 157.7, 155.3, 135.1, 131.8, 130.9, 130.2, 129.3 (2C), 128.0 (2C), 127.9, 125.3, 116.8, 114.2, 108.5, 45.6, 36.7, 28.6, 20.6, 13.1; IR (KBr, cm^−1^): 3054–2864 (C–H), 1738, 1679 (C=O), 1599 (C=C), 1071 (C–O); HRMS (ESI): *m*/*z* [M + H^+^] calcd for monoisotopic mass 440.0987, found 440.1059.

#### 3-[5-Methyl-3-(2-fluoro-6-chlorophenyl)isoxazole-4-carbonyloxy]-2-phenyl-5,5-dimethyl-2-cyclohexen-1-one 3(S25)

White solid; yield 79%; mp 106.6–107.3 °C; ^1^H NMR (600 MHz, CDCl_3_): 6.86–7.45 (m, 8H, Ar–H), 2.62 (s, 2H, CH_2_), 2.56 (s, 3H, C=C–CH_3_), 2.46 (s, 2H, CH_2_), 1.18 (s, 6H, –(CH_3_)_2_); ^13^C NMR (150 MHz, CDCl_3_): 196.9, 177.1, 162.7, 159.3 (2C), 155.5, 135.1, 131.8, 130.8, 129.4, 129.3 (2C), 128.0 (2C), 127.7, 125.2, 116.9, 114.2, 108.5, 51.6, 42.2, 32.8, 27.8 (2C), 13.1; IR (KBr, cm^−1^): 3084–2872 (C–H), 1738, 1680 (C=O), 1600 (C=C), 1071 (C–O); HRMS (ESI): *m*/*z* [M + H^+^] calcd for monoisotopic mass 454.1143, found 454.1216.

#### 3-[(2-Trifluoromethyl-4-methyl)pyrazoloyloxy]-2-phenyl-2-cyclohexen-1-one 3(S26)

White solid; yield 38%; mp 85.9–87.0 °C; ^1^H NMR (400 MHz, CDCl_3_): 7.70 (s, 1H, =CH–N), 7.13–7.32 (m, 5H, Ar–H), 3.90 (s, 3H, N–CH_3_), 2.81–2.86 (t, 2H, *J* = 8.2 Hz, CH_2_), 2.62–2.67 (t, 2H, *J* = 9.0 Hz, CH_2_), 2.17–2.26 (m, 2H, CH_2_); ^13^C NMR (100 MHz, CDCl_3_): 197.7, 164.4, 157.0, 136.8, 131.3, 130.7, 129.6 (2C), 127.9 (2C), 127.7, 121.8, 118.2, 111.3, 39.9, 37.6, 29.0, 20.8; IR (KBr, cm^−1^): 3139–2875 (C–H), 1754, 1672 (C=O), 1540 (C=C), 1168 (C–O); HRMS (ESI): *m*/*z* [M + H^+^] calcd for monoisotopic mass 365.1035, found 365.1108.

#### 3-[(2-Trifluoromethyl-4-methyl)pyrazoloyloxy]-2-phenyl-5-methyl-2-cyclohexen-1-one 3(S27)

White solid; yield 69%; mp 115.9–118.3 °C; ^1^H NMR (600 MHz, CDCl_3_): 7.71 (s, 1H, =CH–N), 7.14–7.37 (m, 5H, Ar–H), 3.92 (s, 3H, N–CH_3_), 2.76–2.82 (m, 1H, CH_2_), 2.70–2.74 (m, 1H, CH_2_), 2.63–2.67 (m, 1H, CH_2_), 2.51–2.52 (m, 1H, CH), 2.34–2.41 (m, 1H, CH_2_), 1.20–1.21 (d, 3H, *J* = 9.6 Hz, –CH_3_); ^13^C NMR (150 MHz, CDCl_3_): 197.8 (2C), 157.0, 142.0, 136.8, 131.2 (2C), 129.6 (2C), 127.9 (2C), 127.7, 118.6, 111.3, 45.8, 39.9, 37.1, 28.6, 20.9; IR (KBr, cm^−1^): 3133–2876 (C–H), 1748, 1677 (C=O), 1542 (C=C), 1171 (C–O); HRMS (ESI): *m*/*z* [M + H^+^] calcd for monoisotopic mass 379.1191, found 379.1264.

#### 3-[(2-Trifluoromethyl-4-methyl)pyrazoloyloxy]-2-phenyl-5,5-dimethyl-2-cyclohexen-1-one 3(S28)

White solid; yield 80%; mp 100.9–102.1 °C; ^1^H NMR (400 MHz, CDCl_3_): 7.70 (s, 1H, =CH–N), 7.14–7.35 (m, 5H, Ar–H), 3.92 (s, 3H, N–CH_3_), 2.72 (s, 2H, CH_2_), 2.52 (s, 2H, CH_2_), 1.24 (s, 6H, –(CH_3_)_2_); ^13^C NMR (150 MHz, CDCl_3_): 197.6, 162.7, 157.1, 142.5, 136.8, 131.2, 129.7, 129.6 (2C), 127.9 (2C), 127.7, 111.4, 100.0, 51.5, 42.8, 39.9, 32.7 (2C), 28.2; IR (KBr, cm^−1^): 3133–2873 (C–H), 1749, 1677 (C=O), 1543 (C=C), 1146 (C–O); HRMS (ESI): *m*/*z* [M + H^+^] calcd for monoisotopic mass 393.1348, found 393.1421.

### X-ray diffraction

**Fig. 1:**
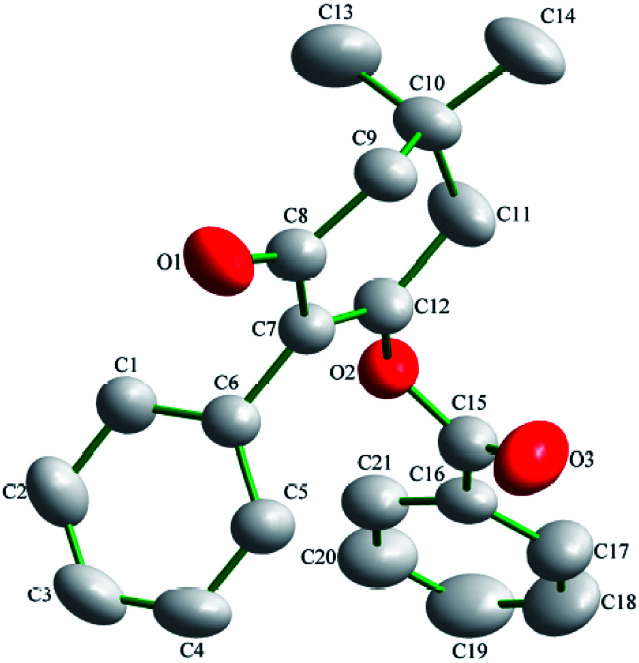
The molecular structure of compound 3(S3).

**Fig. 2:**
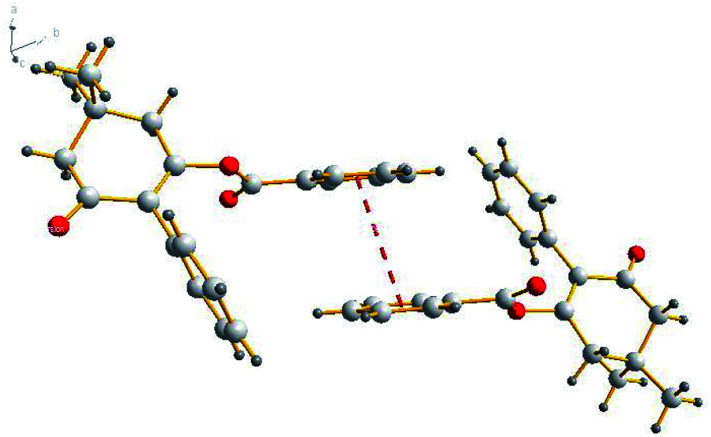
π–π stacking interactions between the core planes.

Suitable single-crystals of compound 3(S3) were recrystallized from ethanol. The molecular structure of compound 3(S3) is shown in Fig. 1. The π–π stacking interaction is shown in Fig. 2. The X-ray data were collected on a Rigaku R-AXIS RAPID diffractometer with graphite-monochromator Mo Kα radiation (*λ* = 0.071073 nm) at 293(2) K. A total of 6888 reflections were measured, of which 3054 independent reflections (*R*_int_ = 0.0233) were obtained in the range of 3.14° < *θ* < 25.00° (*h*, 9 to 9; *k*, −10 to 10; *l*, −15 to 15), and 2357 observed reflections with *I* > 2*σ*(*I*) were used in the refinement on *F*^2^. The structure was solved by direct methods using SHELXS-97 and refined by the least-squares procedures on *F*^2^ (SHELXL-97) in the full matrix anisotropic approximation for all non-hydrogen atoms.^28,29^ Symmetry equivalent reflections were used to optimize the crystal shape and size. The crystallographic data has been deposited at the Cambridge Crystallographic Data Centre as supplementary publication number CCDC 1588796.

### Biological assays

The concentration of the safener and compounds applied in the bioassay was determined after a preliminary screening. Maize seeds were soaked in solutions of synthesized compounds at a concentration of 1 μmol L^−1^ for 12 h, and the control was soaked in water. Then, the seeds were germinated in incubators for 24 h. The seeds were sown in paper cups (10 cm × 15 cm), with seven seeds per cup, and incubated in an incubator with a 12 : 12 h photoperiod, 26.5 ± 1 °C, and 75% relative humidity. The clethodim concentration was set to 10 mL hm^−2^ to cause injury to the maize. The spraying treatment was conducted when the maize had reached the two-leaf stage. After 6 days, the chlorophyll content of the maize was determined. Each treatment was replicated three times.

### Computational methods

The three-dimensional structures of compound 3(S24), compound 3(S12), clethodim and cloquitocet-mexyl were constructed using the sketch module of SYBYL-X 2.0.^30^ Subsequently, the molecules were optimized, and the Gasteiger–Huckel charges were calculated. The crystal structure of ACCase was taken from the Protein Data Bank (PDB ID 3K8X). Docking modeling was achieved using the CDOCKER method in Accelrys Discovery Studio 2.5.^31^ Before docking, the protein structure was given the CHARMM force field, and water and other co-crystallized small molecules were removed. After the protein preparation, the active site was defined, with a subset region of 13.0 Å from the centre of the known ligand. During the docking process, the top 10 conformations were saved for each ligand based on the −CDOCKER_ENERGY after the energy minimization using the smart minimize method in DS 2.5, and the default values were used for the remaining parameters.

## Results and discussion

### Chemistry

**Scheme 2:**
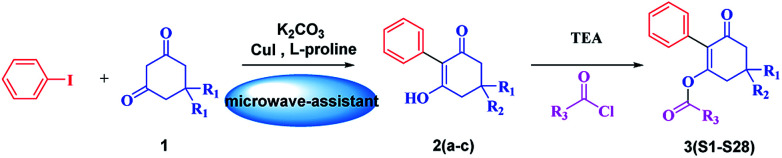
Route for the synthesis of the title compounds.

The synthetic route is depicted in Scheme 2. A series of 3-hydroxy-2-phenylcyclohex-2-en-1-one derivatives (2) were synthesized from substituted 1,3-cyclohexanedione (1) and iodobenzene in DMSO under microwave irradiation, the conditions of which were optimized. Initially, the intermediates 2a–c were synthesized by a coupling reaction in CH_2_Cl_2_ using l-proline and CuI as the catalyst with 25.6–43.8% yields. This promising result indicated that it was feasible to synthesize 3-hydroxy-2-phenylcyclohex-2-en-1-one through a coupling reaction. Subsequently, we investigated the effects of microwave power, time, base and solvents on the coupling reaction. It was found that microwave power was crucial for this reaction. The reaction cannot proceed with a power less than 200 W. Moreover, the reaction yields constantly improved upon increasing the power, and the yields were highest at 800 W. In addition, the optimum yields were achieved by adjusting the reaction time from 5 to 40 min. Furthermore, the bases Et_3_N, NaOH and anhydrous K_2_CO_3_ were employed as the acid-binding agents, and anhydrous K_2_CO_3_ was found to be the best acid-binding agent for this coupling reaction. To further improve the reaction yields, we evaluated the effects of different solvents on the yields. The experimental results indicated that DMSO provided higher yields than CH_2_Cl_2_, THF, and 1,4-dioxane due to polar aprotic solvents being the best at promoting this coupling reaction. The optimum conditions were as follows: 800 W as the power, 40 min as the reaction time, and DMSO as the solvent. The yields were higher, and the reaction time was much shorter than that of the reported conventional method^32^ (Table 1).

**Table 1:** Comparisons of the conventional and microwave irradiation methods

Compound	Conventional method		Microwave irradiation method	
	Time (h)	Yield (%)	Time (min)	Yield (%)
2a	48 h	64	40 min	79
2c	48 h	35	40 min	88

**Scheme 3:**
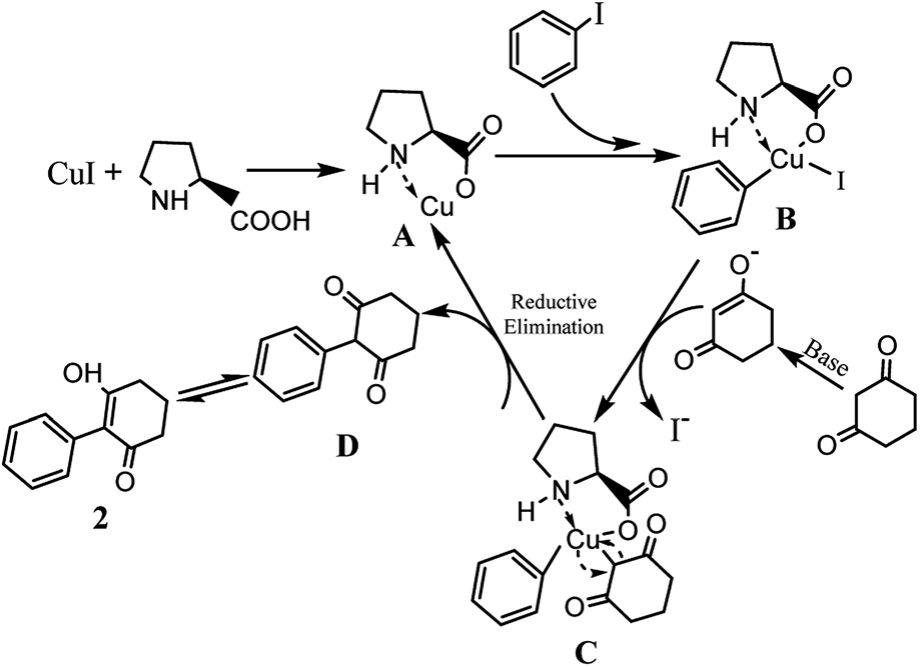
Reaction mechanism of intermediates.

The possible reaction mechanism outlined in Scheme 3 is similar to that reported.^33,34^l-proline and Cu(i) formed complex A, and the chelation of l-proline with Cu(i) makes the Cu(i) more reactive towards an oxidative addition and stabilizes the formed intermediate B, facilitating the coupling reaction. Subsequently, the iodine coordinated to the copper was exchanged for the carbanion of the activated methylene group of 1,3-cyclohexanedione, forming the intermediate C*via* a reductive elimination, releasing the coupling product D and regenerating the active Cu(i) catalyst A.

The substituted 2-phenyl-2-cyclohexanedione enol ester derivatives 3 were prepared from the intermediates 2 and different acyl chlorides, with yields of 33–91% (Table 2). Et_3_N was employed to promote the acylation in the positive direction as an acid-attaching agent.

**Table 2:** The structures of the novel substituted 2-phenyl-2-cyclohexanedione enol ester compounds

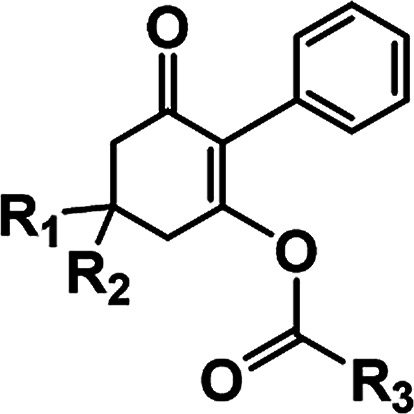					
Compound	R_1_	R_2_	R_3_	Yield (%)	Melting point (°C)
3(S1)	H	H	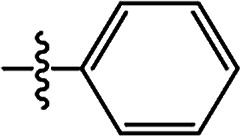	50	76.5–78.1
3(S2)	CH_3_	H	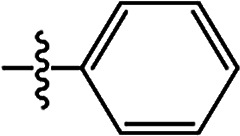	56	80.2–81.0
3(S3)	CH_3_	CH_3_	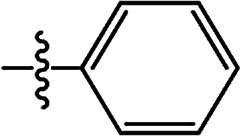	65	84.3–86.2
3(S4)	H	H	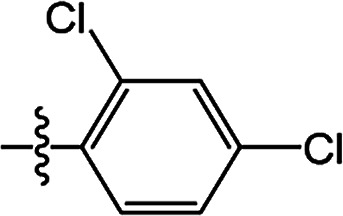	59	103.9–104.9
3(S5)	CH_3_	H	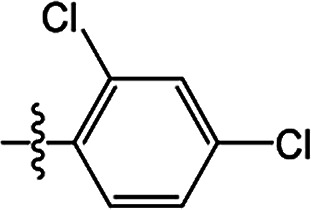	62	73.3–74.3
3(S6)	CH_3_	CH_3_	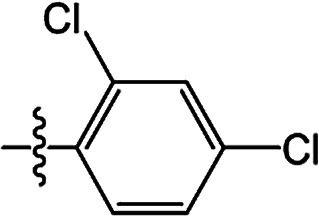	70	—
3(S7)	H	H	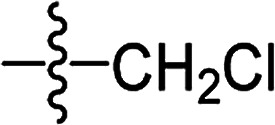	33	—
3(S8)	CH_3_	H	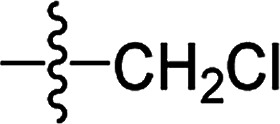	35	—
3(S9)	CH_3_	CH_3_	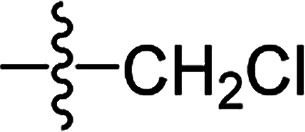	40	—
3(S10)	CH_3_	CH_3_	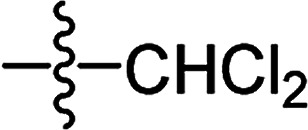	48	79.6–81.3
3(S11)	H	H	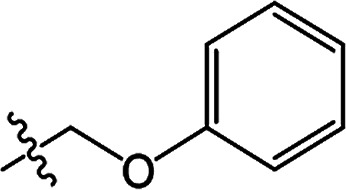	50	80.1–81.7
3(S12)	CH_3_	H	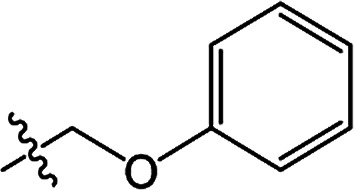	57	—
3(S13)	CH_3_	CH_3_	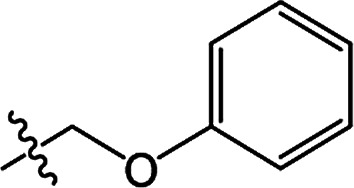	64	84.7–86.7
3(S14)	H	H	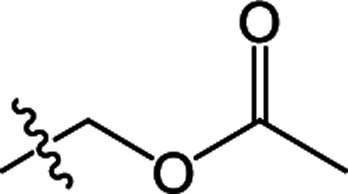	39	—
3(S15)	CH_3_	H	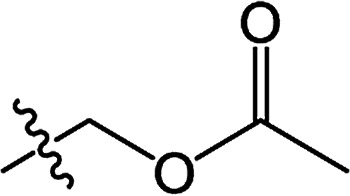	43	—
3(S16)	CH_3_	CH_3_	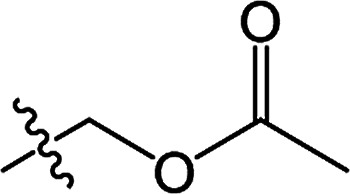	47	—
3(S17)	H	H	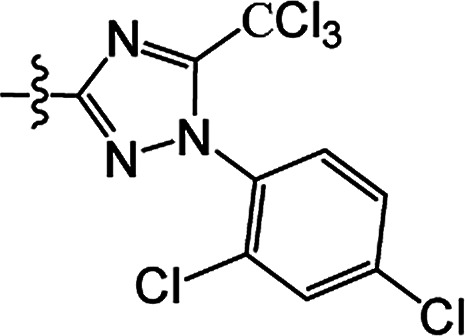	46	172.4–173.1
3(S18)	CH_3_	H	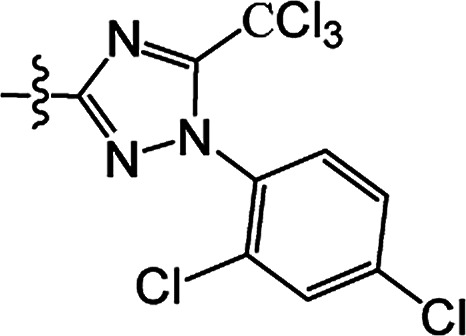	54	193.9–195.1
3(S19)	CH_3_	CH_3_	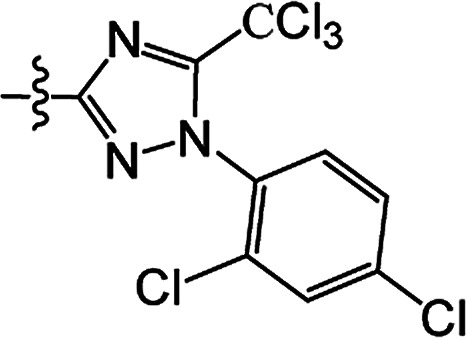	80	180.8–182.0
3(S20)	H	H	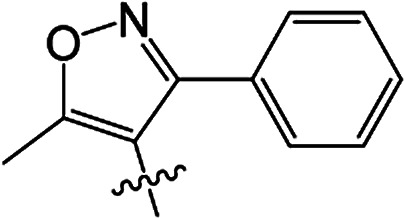	79	99.3–101.1
3(S21)	CH_3_	H	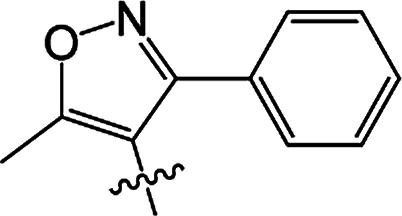	83	—
3(S22)	CH_3_	CH_3_	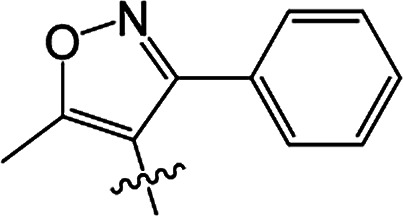	91	105.2–106.9
3(S23)	H	H	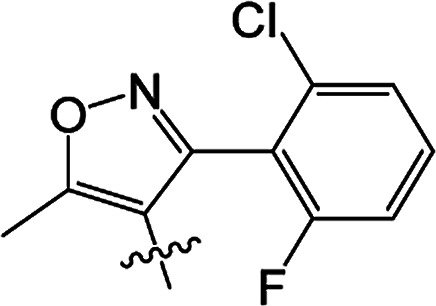	59	—
3(S24)	CH_3_	H	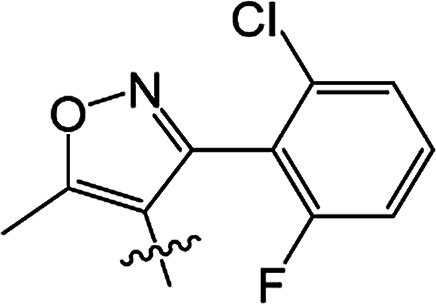	70	143.3–144.2
3(S25)	CH_3_	CH_3_	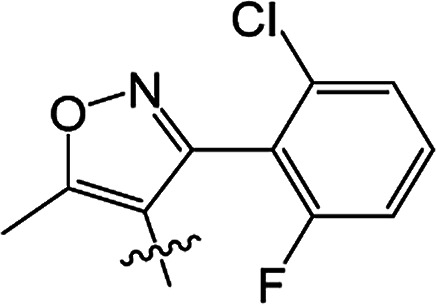	79	106.6–107.3
3(S26)	H	H	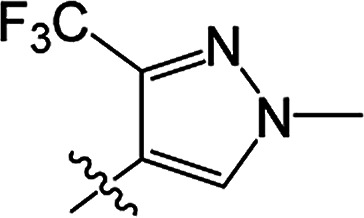	38	85.9–87.0
3(S27)	CH_3_	H	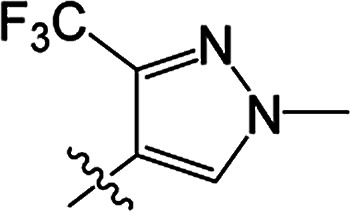	69	115.9–118.3
3(S28)	CH_3_	CH_3_	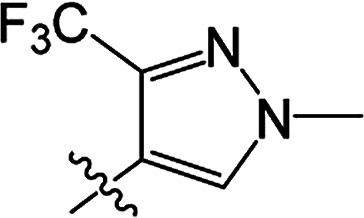	80	100.9–102.1

In general, there were significant effects on the yields of the target products caused by the presence of electron-donating groups. Notably, the presence of a methyl group at the 5-position of the cyclohexanedione increased the yields significantly. Two methyl groups in the 5-position of cyclohexanedione provided better yields than one methyl group, and the yields were lowest when there was no substitution. For example, the yield of 3(S28) was significantly higher than that of 3(S26).

All of the structures were characterized by IR, ^1^H NMR, ^13^C NMR and HRMS spectroscopies. The peaks at 1732 and 1677 cm^−1^ in the IR for compound 3(S3) confirmed the presence of the carbonyl groups. The ^1^H NMR spectrum also confirmed the proposed structure. The signals at *δ* 6.88–7.45 ppm were related to the benzene rings. The single signal observed at *δ* 1.26 ppm was characteristic of the two methyl groups linked to the cyclohexanedione.

### Structure analysis

The molecular structure of compound 3(S3) is shown in Fig. 1. Compound 3(S3) contains a cyclohexanedione ring and two benzene rings. Cyclohexanedione adopts a half-chair conformation structure. The cyclohexanedione and benzene ring II [C16, C17, C18, C19, C20 and C21] are not coplanar, with a dihedral angle of 63.803 (62)°. Benzene ring II [C16, C17, C18, C19, C20 and C21] is also not coplanar with benzene ring I [C1, C2, C3, C4, C5 and C6] with a dihedral angle of 62.933 (65)°. The π–π stacking interaction is shown in Fig. 2. Within the double chain, weak π–π packing interactions exist between the two benzene rings. These interactions further reinforce the supramolecular structural stability. No significant hydrogen bonding was found in the crystal structure.

### Biological activity

The *in vivo* safener activities of the title compound (1 μmol L^−1^) against clethodim were evaluated (Table 3). The chlorophyll content was measured after treatment with clethodim for 6 d.

**Table 3:** The chlorophyll content of maize treated with the target compounds (1 μmol L^−1^)^a^^,^^b^

Compound	Chlorophyll content (mg g^−1^)	Compound	Chlorophyll content (mg g^−1^)	Compound	Chlorophyll content (mg g^−1^)
Clethodim	14.9 ± 0.3	3(S9)	15.5 ± 0.5	3(S19)	16.3 ± 1.0
Cloquitocet-mexyl	17.6 ± 0.8	3(S10)	16.3 ± 0.4	3(S20)	16.8 ± 0.6
3(S1)	16.7 ± 1.2	3(S11)	15.8 ± 0.9	3(S21)	17.3 ± 0.4
3(S2)	15.6 ± 0.9	3(S12)	13.8 ± 1.4	3(S22)	18.2 ± 1.2
3(S3)	17.6 ± 1.3	3(S13)	16.3 ± 1.2	3(S23)	19.9 ± 0.7
3(S4)	15.1 ± 1.1	3(S14)	14.3 ± 0.6	3(S24)	20.5 ± 0.5
3(S5)	15.8 ± 0.3	3(S15)	16.1 ± 0.8	3(S25)	15.3 ± 1.1
3(S6)	16.5 ± 0.6	3(S16)	15.7 ± 0.5	3(S26)	16.1 ± 0.6
3(S7)	15.5 ± 0.9	3(S17)	16.1 ± 1.1	3(S27)	15.9 ± 0.9
3(S8)	16.1 ± 1.2	3(S18)	16.6 ± 1.2	3(S28)	15.3 ± 1.0

a Data are the means of three replicates.

b Water treated was used as a contrast and the herbicide is clethodim.

Photosynthesis is an indispensable physiological activity in the process of plant growth. The chlorophyll content in leaves directly affects photosynthesis sufficiently to promote plant growth and plant nutrient accumulation. Clethodim can provoke an obvious decrease in the chlorophyll content of maize, but significant differences were observed after the initial introduction of the compounds. Compounds 3(S1–S28) showed some recovery of chlorophyll content. Among the compounds tested, compound 3(S24) showed the best activity against the injury of clethodim, even better than that of the commercial safener cloquitocet-mexyl.

From a structural perspective, the introduction of the subunit of 5-methyl-3-phenylisoxazole exhibited excellent safener activity in protecting maize. The 5-position substituents on the cyclohexanedione were further studied. It was noticeable that the presence of a methyl group at the 5-position gave rise to a positive improvement in the safener activity. Two methyl groups at the 5-position provided higher safener activity than one methyl group, and the safener activity was the lowest when there was no substitution. The results indicate that the 5-position substituents were greatly associated with safener activity.

The greenhouse experiments showed that compound 3(S24) exhibited the best safener activity, and compound 3(S12) showed the worst. To prove the hypothesis that safeners may compete for the target site with herbicides, the chemical properties of the compounds 3(S12) and 3(S24), clethodim and cloquitocet-mexyl were compared, such as the log *p*, aromatic rings, surface area and electronegativity (Table 4). It was observed that the log *p*, aromatic rings, surface area and electronegativity of compound 3(S24) were all similar to those of the safener cloquitocet-mexyl. The properties of compound 3(S12) regarding the log *p*, surface area and electronegativity shared a similarity with the herbicide clethodim. This indicated that, in terms of the investigated features and SAR theory, compound 3(S24) may be a lead candidate compound for use as a novel safener, and compound 3(S12) might be a candidate for a potential herbicide.

**Table 4:** Comparisons of the chemical properties of clethodim, cloquitocet-mexyl, 3(S24) and 3(S12)

Compounds	log *p*^a^	Aromatic rings^a^	Surface area^b^	Rotatable bonds^b^	Molecular weight^a^	Electronegativity^c^
Clethodim	2.23	0	363.73	9	359.90	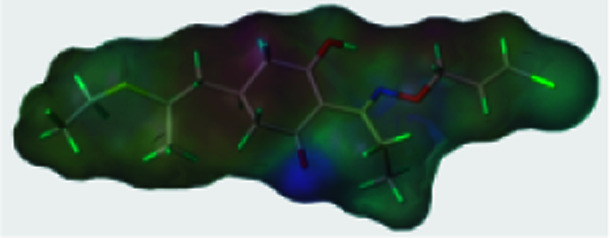
Cloquitocet-mexyl	4.47	2	332.46	9	335.12	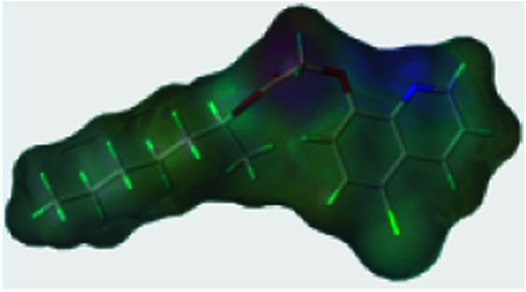
3(S24)	4.59	3	322.51	5	424.83	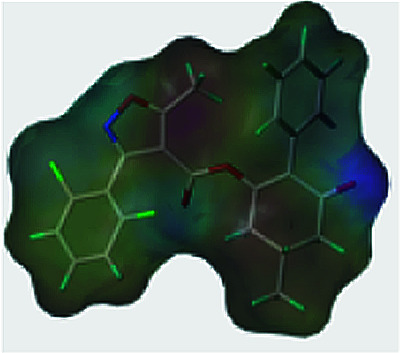
3(S12)	2.85	2	400.15	6	336.38	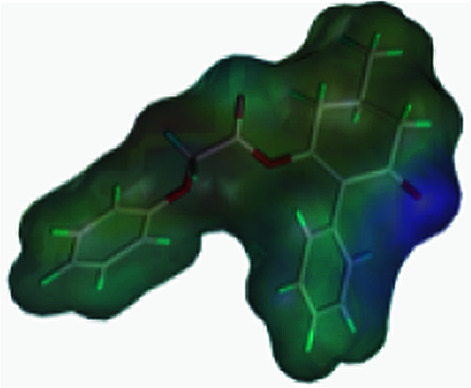

a The log *p*, numbers of aromatic rings and molecular weights were predicted by ChemBioOffice 2014.

b The surface areas and number of rotatable bonds were predicted by Discovery Studio 2.5.

c The electronegativity was predicted by Sybyl-X 2.0 (Tripos Inc., St. Louis, MO).

**Fig. 3:**
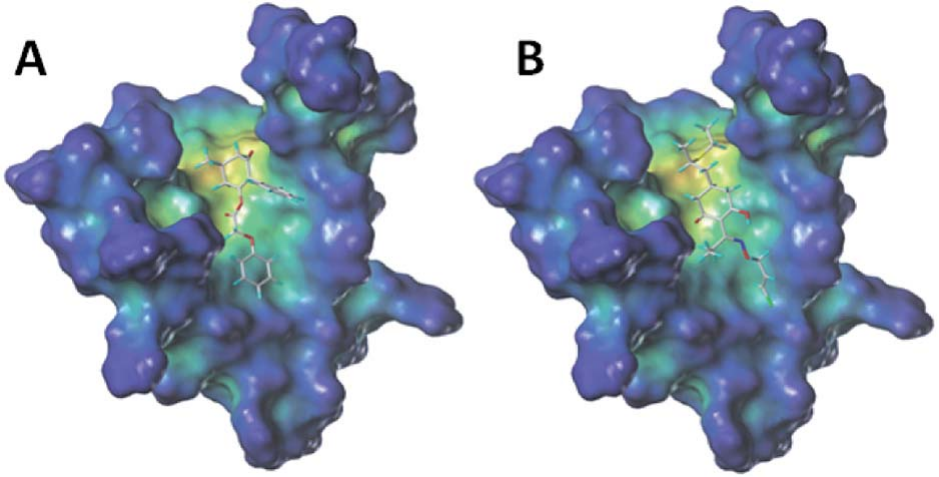
The docking modeling of compound 3(S12) (A) and clethodim (B) with ACCase at the active site. The carbon atoms are shown in grey, the hydrogen atoms are shown in cyan, the sulphur atoms are shown in light yellow, the oxygen atoms are shown in red, and the nitrogen atoms are shown in light blue.

To further study the effect of the safener on the target enzyme of the herbicides, a molecular docking experiment was conducted. It showed that both compound 3(S24) and cloquitocet-mexyl had no interactions with the surrounding residues. Interestingly, the docking score of both compound 3(S24) and cloquitocet-mexyl were negative in value, representing a bad docking result. However, compound 3(S12) and clethodim were well matched to the active site of ACCase (Fig. 3).

**Fig. 4:**
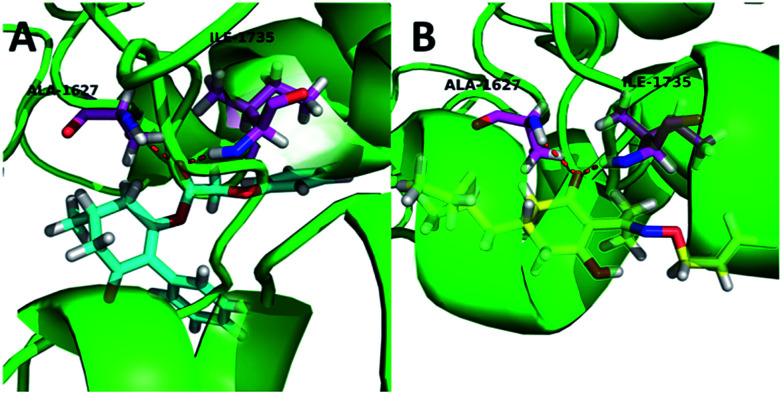
The receptor–ligand interaction of 3(S12) and clethodim with the active site of ACCase, and 3(S12) and clethodim are shown in cyan and yellow, respectively.

Compound 3(S12) and clethodim have a similar binding position at the active site, and this indicated the excellent similarity between the activities of compound 3(S12) and clethodim. As shown in Fig. 4, the ester carbonyl formed two H-bond interactions with Ala1627 and ILE1735 in the protein hinge region. Similarly, the cyclohexanedione group of clethodim generated two H-bond interactions with Ala1627 and ILE1735. Based on the facts mentioned above, the theory of competing with the herbicide at the active site might be ruled out; in connection with the results of greenhouse experiments, we speculated that antagonism potentially existed between compound 3(S24) and clethodim in maize, which protected the maize from injury by clethodim.

## Conclusion

In conclusion, a novel class of substituted 2-phenyl-2-cyclohexanedione enol ester derivatives were designed and synthesized based on the SAR and active substructure combination. The intermediate 3-hydroxy-2-phenylcyclohex-2-en-1-one derivatives were synthesized using microwave irradiation, and the reaction conditions were optimized. The bioactivity results demonstrated that most of the compounds showed excellent safener activity; compound 3(S24) displayed inspired safener activity in protecting maize from clethodim injury comparable to that of the commercial safener cloquitocet-mexyl. Molecular docking experiments revealed that the promising safener potency may be ascribed to the antagonism between the compounds 3(S24) and clethodim. The present results provide a powerful complement to the SAR in designing bioactive molecules. Thus, substituted 2-phenyl-2-cyclohexanedione enol ester derivatives have emerged as novel lead compounds for developing new safener agents.

## Conflicts of interest

The authors have no conflicts of interest to declare.

## Supplementary Material

RA-008-C8RA02647E-s001

RA-008-C8RA02647E-s002

## References

[cit1] Zhou Q., He X., Liu F., Li Y., Chen C., Peng Q. (2017). J. Mol. Liq..

[cit2] Shergill L. S., Malone J., Boutsalis P. (2017). Pest Manage. Sci..

[cit3] Laforest M., Soufiane B., Simard M. J., Obeid K., Page E., Nurse R. E. (2017). Pest Manage. Sci..

[cit4] Sasaki Y., Nagano Y. (2004). Biosci., Biotechnol., Biochem..

[cit5] Elmore M. T., Brosnan J. T., Armel G. R., Vargas J. J., Breeden G. K. (2015). Weed Technol..

[cit6] Mcquarters A. B., Wolf M. W. (2014). Angew. Chem..

[cit7] Riechers D. E., Kreuz K., Zhang Q. (2010). Plant Physiol..

[cit8] Jeschke P. (2016). Pest Manage. Sci..

[cit9] Busi R., Nguyen N. K., Chauhan B. S., Vidotto F., Tabacchi M., Powles S. B. (2016). Pest Manage. Sci..

[cit10] Williams II M. M., Pataky J. K. (2010). Weed Sci..

[cit11] Bunting J. A., Sprague C. L., Riechers D. E. (2004). Weed Sci..

[cit12] Paporisch A., Rubin B. (2017). Pestic. Biochem. Physiol..

[cit13] Cummins I., Dixon D. P., Freitag-Pohl S., Skipsey M., Edwards R. (2011). Drug Metab. Rev..

[cit14] Cataneo A. C., Ferreira L. C., Mischan M. M., Velini E. D., Corniani N., Cerdeira A. L. (2013). Planta Daninha.

[cit15] Jablonkai I. , Herbicide Safeners: Effective Tools to Improve Herbicide Selectivity, ed. A. J. Price and J. A. Kelton, InTech, Croatia, 2013, ch. 23, pp. 589–620

[cit16] Kraehmer H., Laber B., Rosinger C., Schulz A. (2014). Plant Physiol..

[cit17] Del B. D., Scarponi L., Espen L. (2007). Phytochemistry.

[cit18] Stephenson G. R. and Chang F. Y., Comparative activity and selectivity of herbicide antidotes, ed. F. Pallos, Elsevier Inc., Canada, 1978, pp. 35–61

[cit19] Zheng Y., Liu B., Gou Z., Liu Y., Zhang X., Wang Y. (2015). Bioorg. Med. Chem..

[cit20] Yu H., Cheng Y., Xu M., Song Y. Q., Luo Y. M., Li B. (2016). J. Agric. Food Chem..

[cit21] Ahrens H., Lange G., Muller T., Rosinger C., Willms L., van Almsick A. (2013). Angew. Chem., Int. Ed..

[cit22] Hou Z., Zhu L. F., Yu X. C., Sun M. Q., Miao F., Zhou L. (2016). J. Agric. Food Chem..

[cit23] Li S. K., Li D. D., Xiao T. F., Zhang S. S., Song Z. H., Ma H. Y. (2016). J. Agric. Food Chem..

[cit24] Fu Y., Wang J. Y., Zhang D., Chen Y. F., Gao S., Zhao L. X., Ye F. (2017). Molecules.

[cit25] Ye F., Wang C., Ma P., Zhao L. X., Gao S., Fu Y. (2018). J. Heterocycl. Chem..

[cit26] Fu Y., Wang M. X., Zhang D., Hou Y. W., Gao S., Zhao L. X. (2017). RSC Adv..

[cit27] Goldblum A., Mechoulam R. (1977). J. Chem. Soc., Perkin Trans. 1.

[cit28] Sheldrick G. M. , SHELXS-97, Program for X-ray Crystal Structure Solution, University of Göttingen, Göttingen, Germany

[cit29] Sheldrick G. M. , SHELXS-97, Program for X-ray Crystal Structure Refinement, University of Göttingen, Göttingen, Germany

[cit30] Sybyl, Version 6.9, Tripos Inc., St. Louis, MO

[cit31] Catalyst, Version 4.10, Accelrys Inc., San Diego, CA, USA, 2005

[cit32] Reddy C. S., Khan I., Lam H. W. (2012). Angew. Chem., Int. Ed..

[cit33] Sperotto E., van Klink G. P., Van K. G., de Vries J. G. (2010). Dalton Trans..

[cit34] Monnier F., Taillefer M. (2009). Angew. Chem..

