# Supplementary material for: Design, microwave-assisted synthesis, bioactivity and SAR of novel substituted 2-phenyl-2-cyclohexanedione enol ester derivatives
Source: RSC Adv. 2018 May 29;8(35):19883–93. doi: 10.1039/c8ra02647e (PMC9080744; doi:10.1039/c8ra02647e)
Supplement: RA-008-C8RA02647E-s001 [file RA-008-C8RA02647E-s001.pdf]

Electronic Supplementary Material (ESI) for RSC Advances.
This journal is © The Royal Society of Chemistry 2018

Design, Microwave-assistant Synthesis, Bioactivity and SAR of Novel Substituted 2-Phenyl-2-Cyclohexanedione Enol Ester Derivatives

Fei Ye, Peng Ma, Yue Zhai, Fei Yang, Shuang Gao, Li-Xia Zhao, Ying Fu*

Supporting information

3-Hydroxy -2-phenylcyclohex-2-en-1-one (2a)

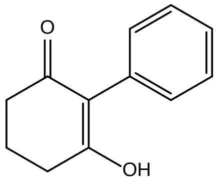

$C_{12}H_{12}O_2$

IR

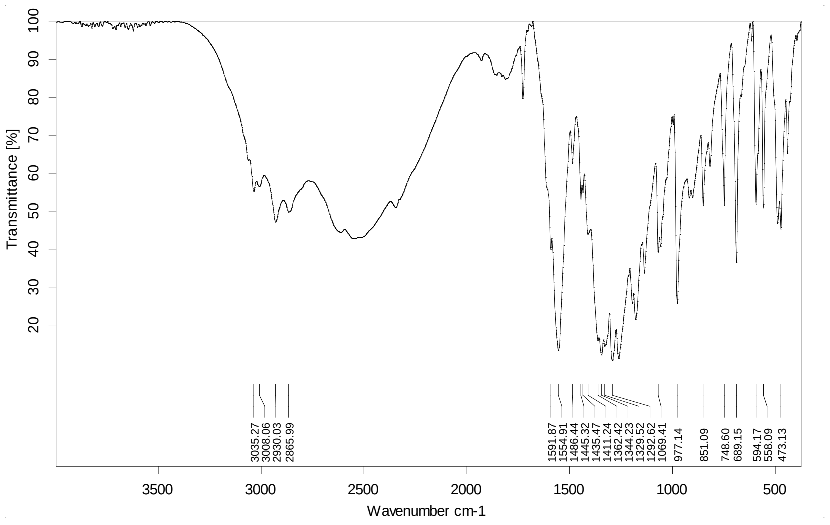

F:\Sample description.153 Sample description Instrument type and / or accessory	21/04/2018

Page 1/1

^{1}H NMR

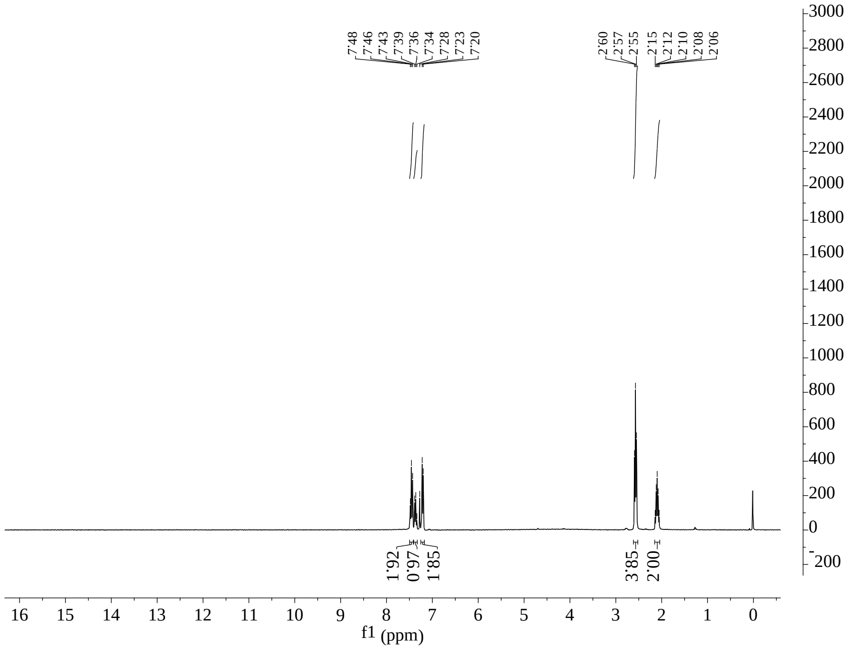

^{13}C NMR

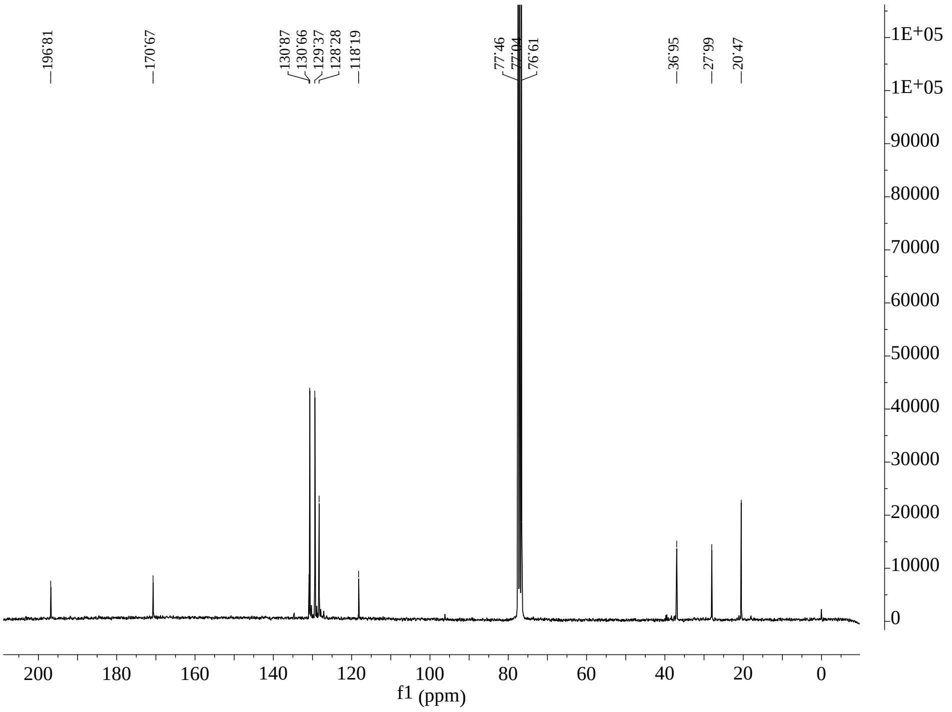

196.81
170.67
130.87
130.66
129.37
128.28
118.19
77.46
77.04
76.61
36.95
27.99
20.47
1E+05
1E+05
90000
80000
70000
60000
50000
40000
30000
20000
10000
0
200
180
160
140
120
100
80
60
40
20
0
f1 (ppm)

3-Hydroxy-5-methyl-2-phenylcyclohex-2-en-1-one (2b)

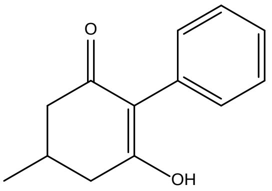

$C_{13}H_{14}O_2$

IR

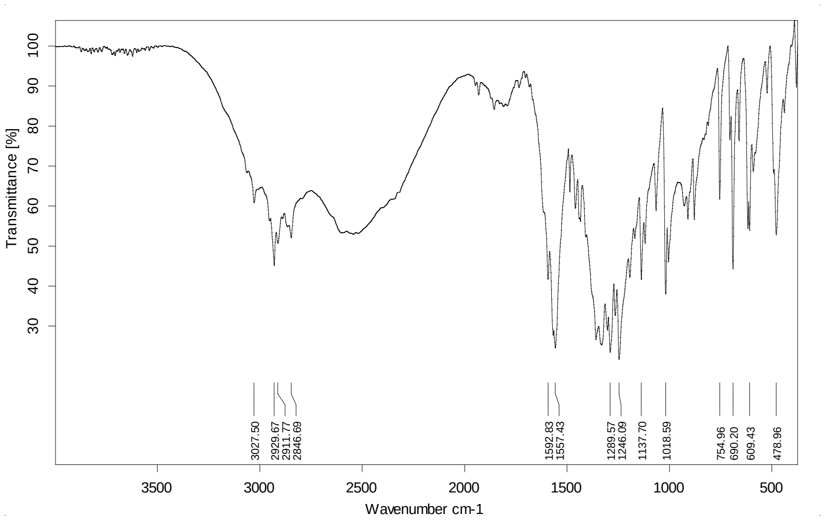

^{1}H NMR

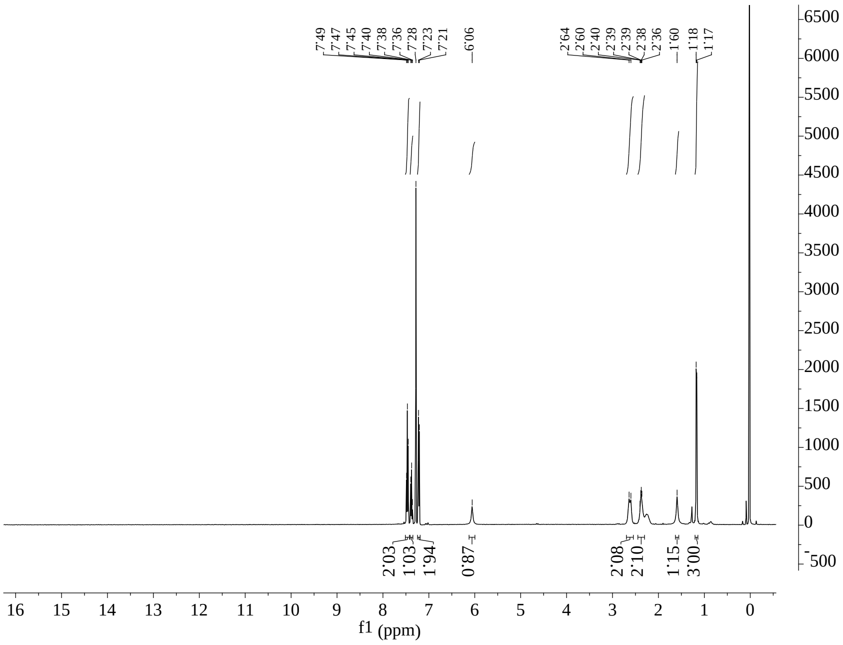

^{13}C NMR

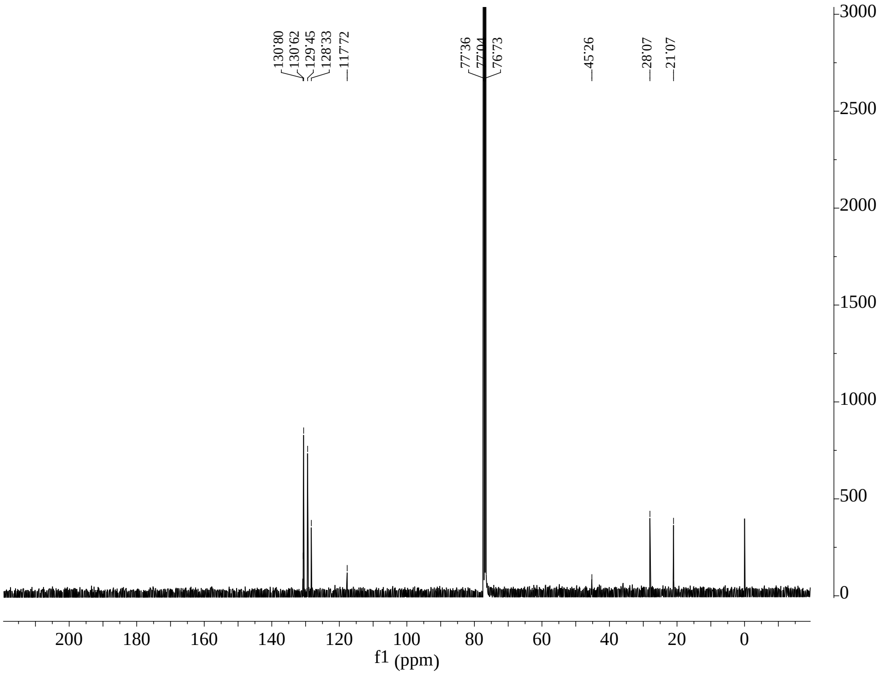
130.80
130.62
129.45
128.33
117.72
77.36
77.04
76.73
45.26
28.07
21.07
3000
2500
2000
1500
1000
500
0
200 180 160 140 120 100 80 60 40 20 0
f1 (ppm)

3-Hydroxy-5,5-dimethyl-2-phenylcyclohex-2-en-1-one (2c)

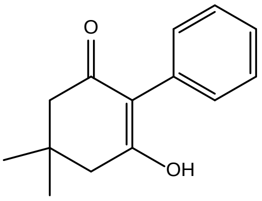

$C_{14}H_{16}O_2$

IR

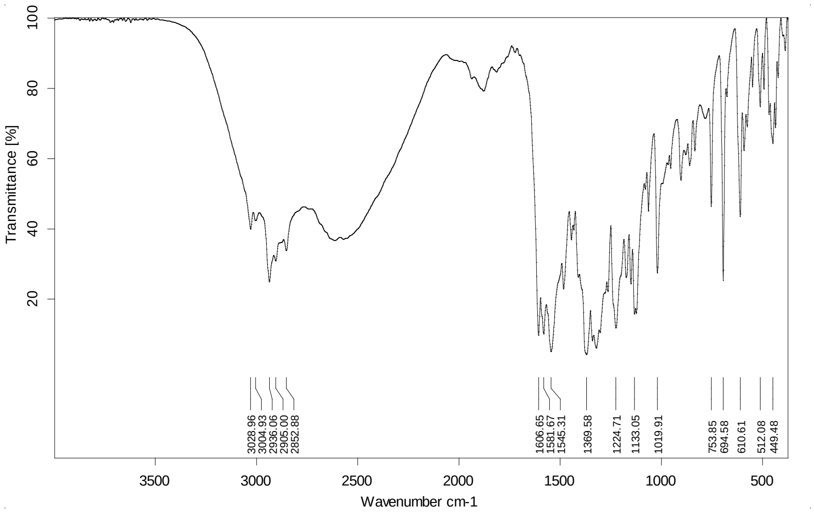

^{1}H NMR

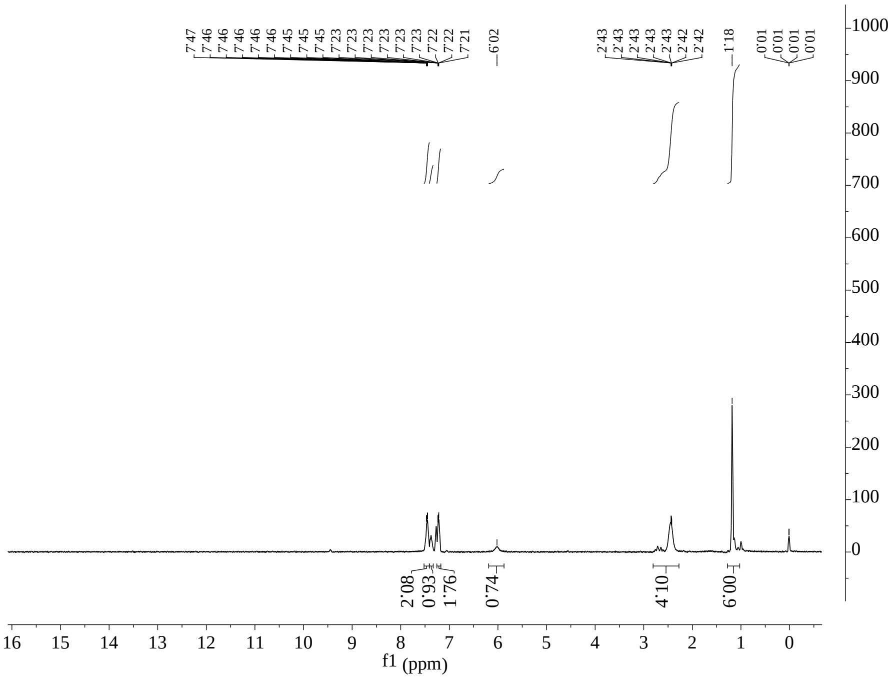

^{13}C NMR

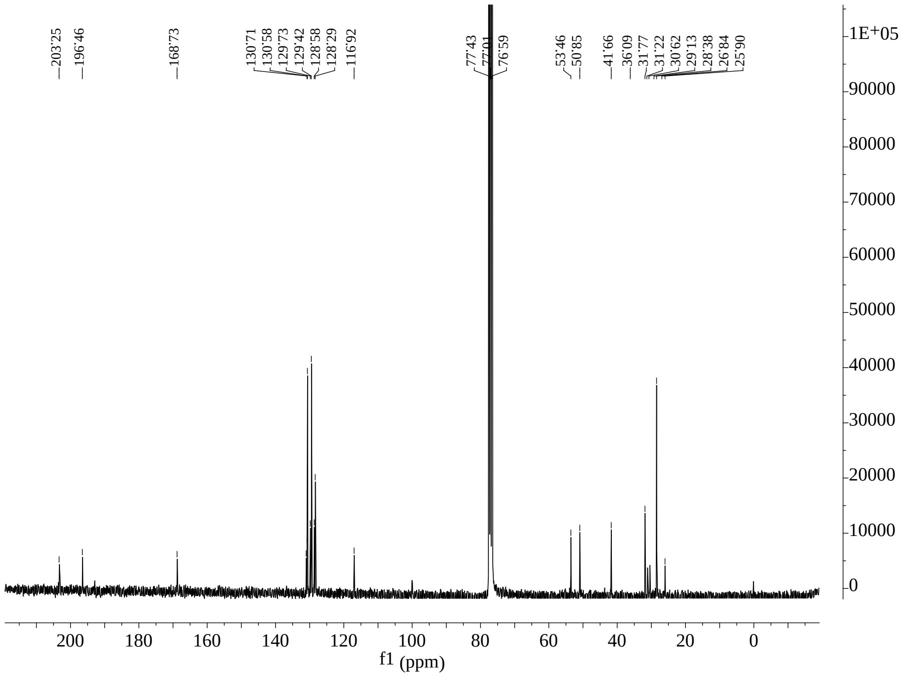

203.25
196.46
168.73
130.71
130.58
129.73
129.42
128.58
128.29
116.92
77.43
77.01
76.59
53.46
50.85
41.66
36.09
31.77
31.22
30.62
29.13
28.38
26.84
25.90
1E+05
90000
80000
70000
60000
50000
40000
30000
20000
10000
0
200
180
160
140
120
100
80
60
40
20
0
f1 (ppm)

3-(Benzoyloxy)-2-phenyl-2-cyclohexen-1-one (S1)

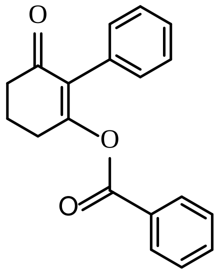

$C_{19}H_{16}O_3$

IR

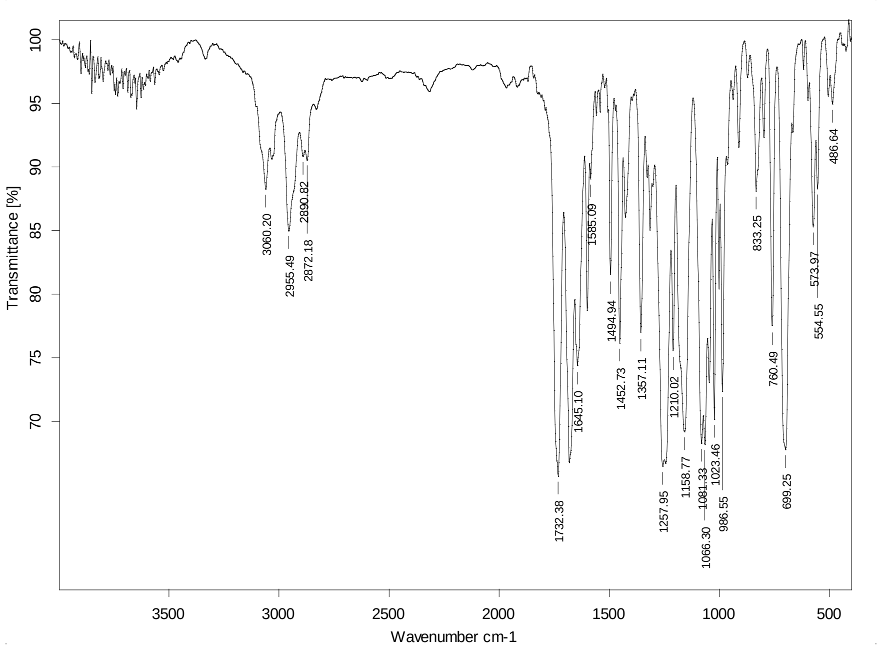

^{1}H NMR

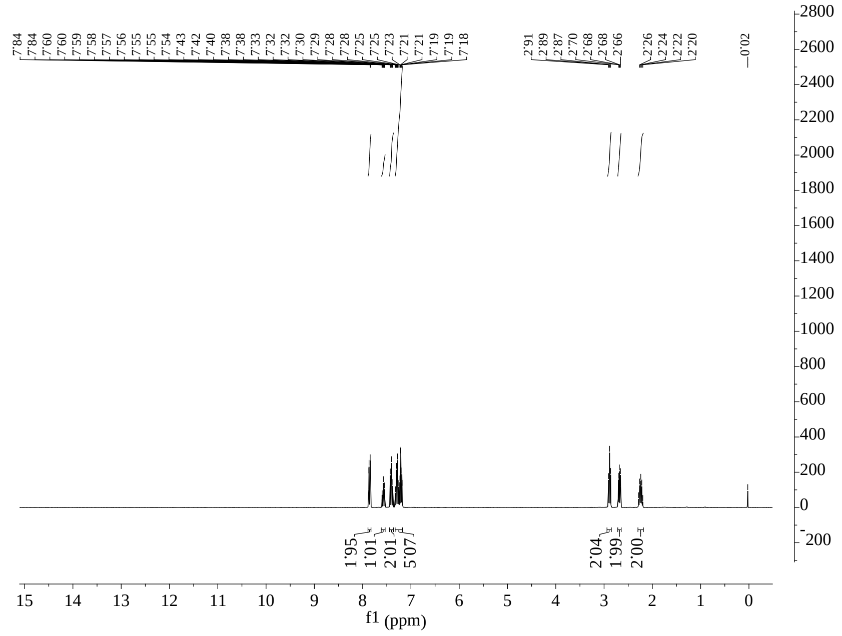

^{13}C NMR

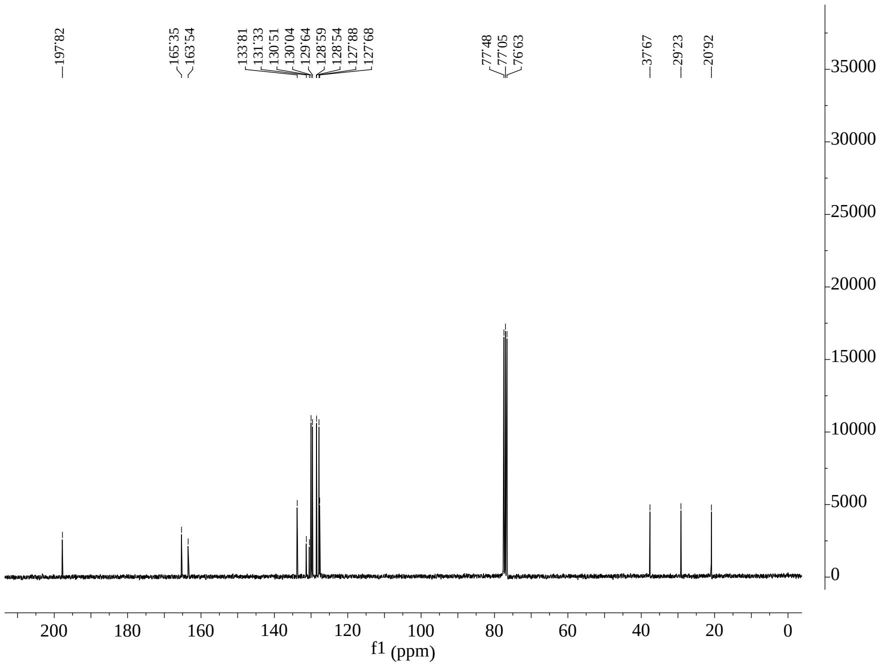
197.82
165.35
163.54
133.81
131.33
130.51
130.04
129.64
128.59
128.54
127.88
127.68
77.48
77.05
76.63
37.67
29.23
20.92
f1 (ppm)

HRMS

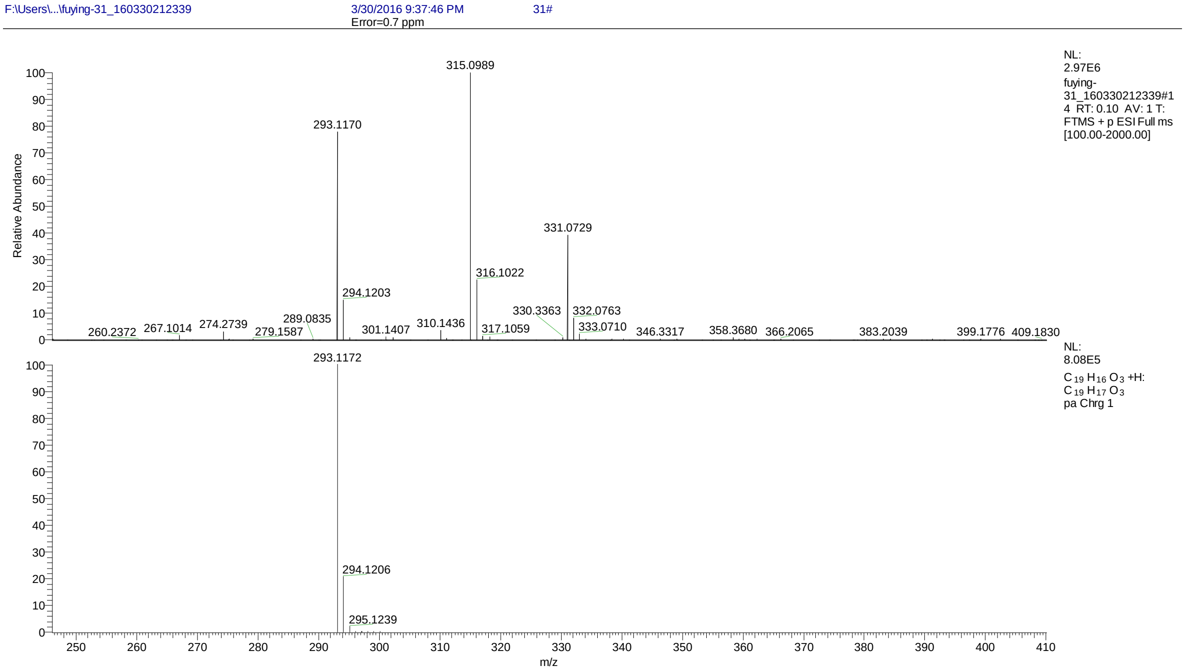
F:\Users\...\fuying-31_160330212339
3/30/2016 9:37:46 PM
31#
Error=0.7 ppm
NL: 2.97E6
fuying-31_160330212339#14 RT: 0.10 AV: 1 T: FTMS + p ESI Full ms [100.00-2000.00]
315.0989
293.1170
331.0729
316.1022
294.1203
NL: 8.08E5
C 19 H16 O3 +H: C 19 H17 O3 pa Chrg 1
293.1172
294.1206
295.1239
Relative Abundance
m/z

3-(Benzoyloxy)-2-phenyl-5-methyl-2-cyclohexen-1-one (S2)

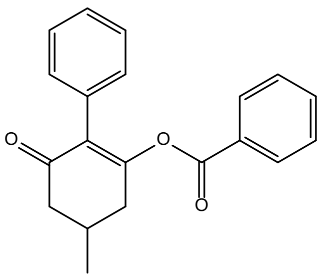

$C_{20}H_{18}O_3$

IR

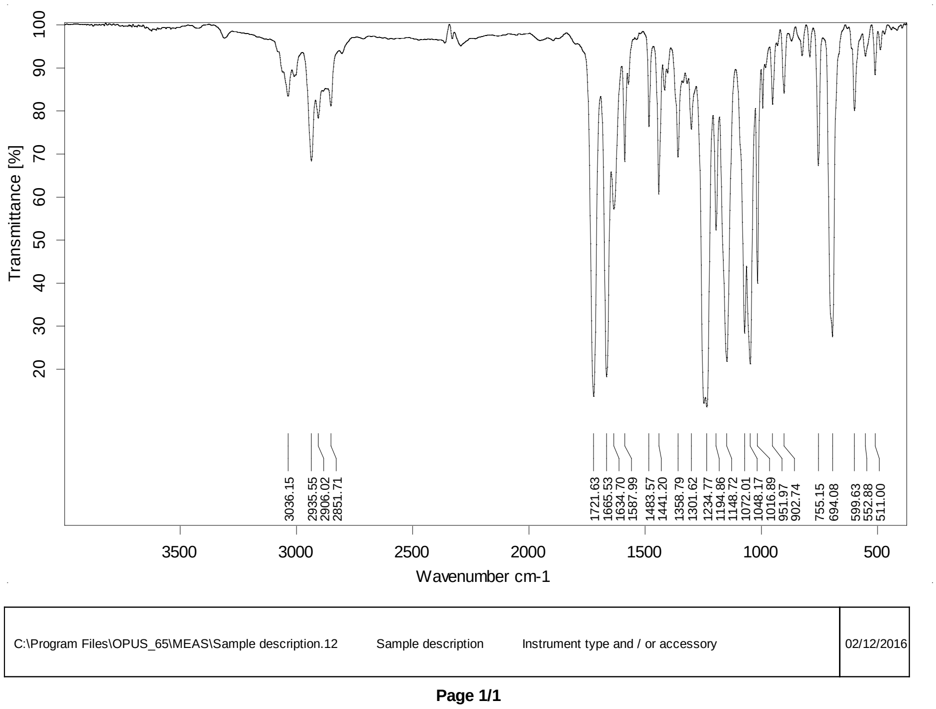

^{1}H NMR

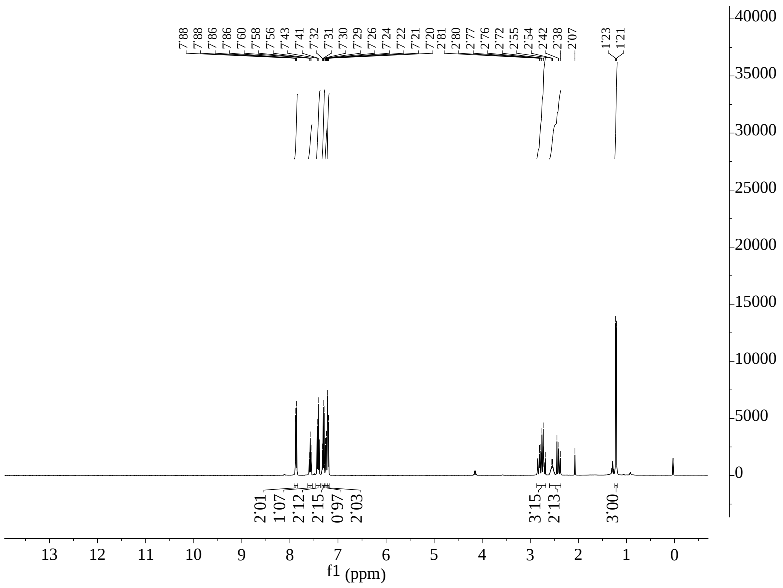

^{13}C NMR

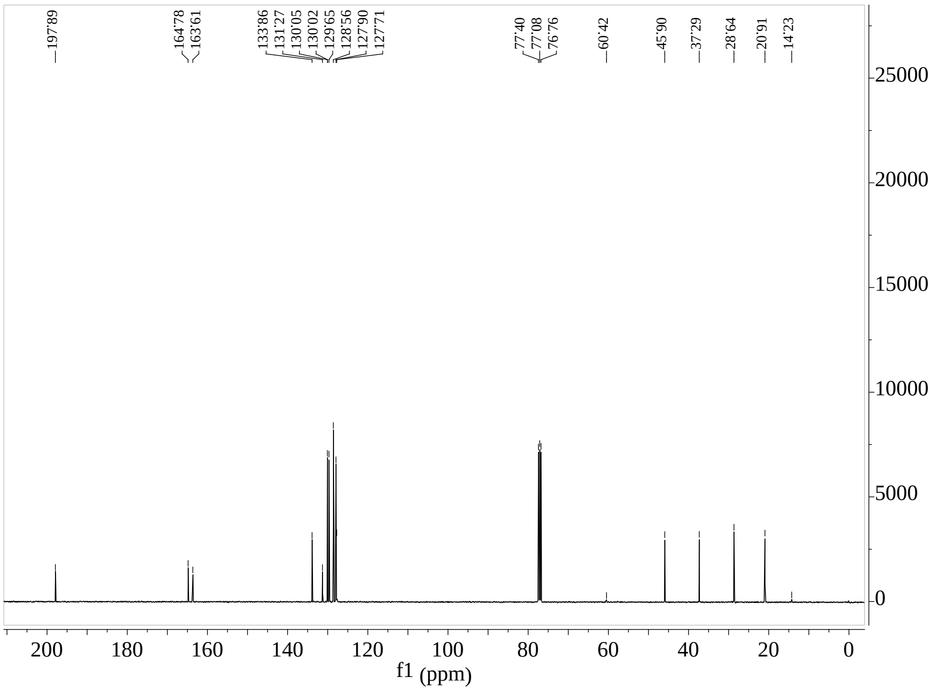

197.89
164.78
163.61
133.86
131.27
130.05
130.02
129.65
128.56
127.90
127.71
77.40
77.08
76.76
60.42
45.90
37.29
28.64
20.91
14.23
f1 (ppm)

HRMS

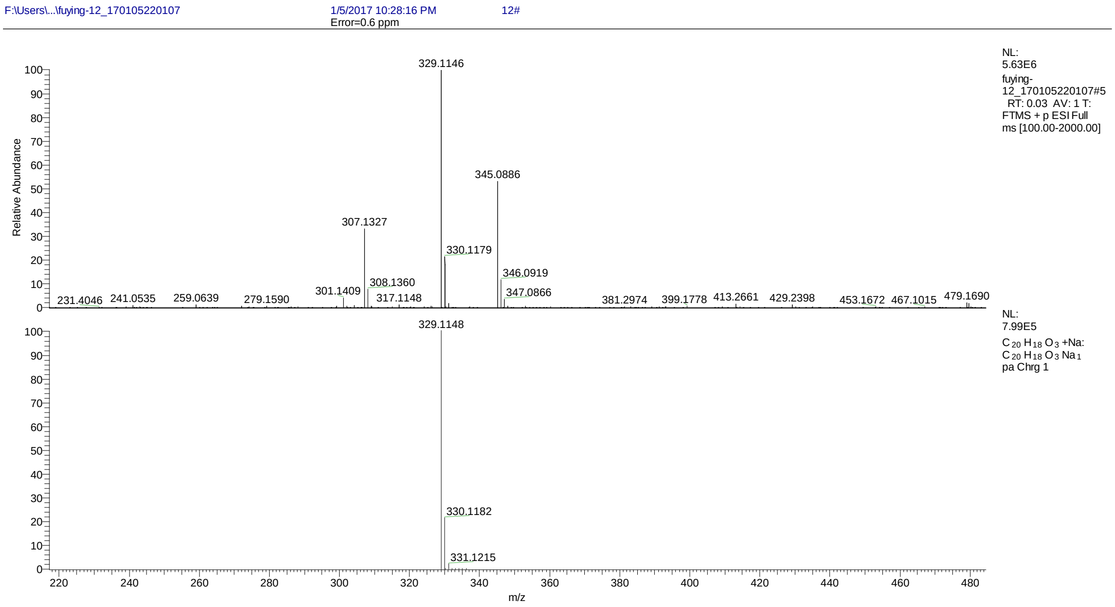

F:\Users\...\fuying-12_170105220107
1/5/2017 10:28:16 PM
12#
Error=0.6 ppm
NL: 5.63E6
fuying-12_170105220107#5 RT: 0.03 AV: 1 T: FTMS + p ESI Full ms [100.00-2000.00]
329.1146
345.0886
307.1327
330.1179
346.0919
308.1360
347.0866
NL: 7.99E5
C 20 H18 O3 +Na: C 20 H18 O3 Na1 pa Chrg 1
329.1148
330.1182
331.1215
Relative Abundance
m/z

3-(Benzoyloxy)-2-phenyl-5,5-dimethyl-2-cyclohexen-1-one (S3)

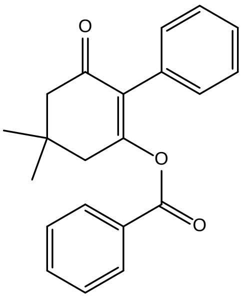

$C_{21}H_{20}O_3$

IR

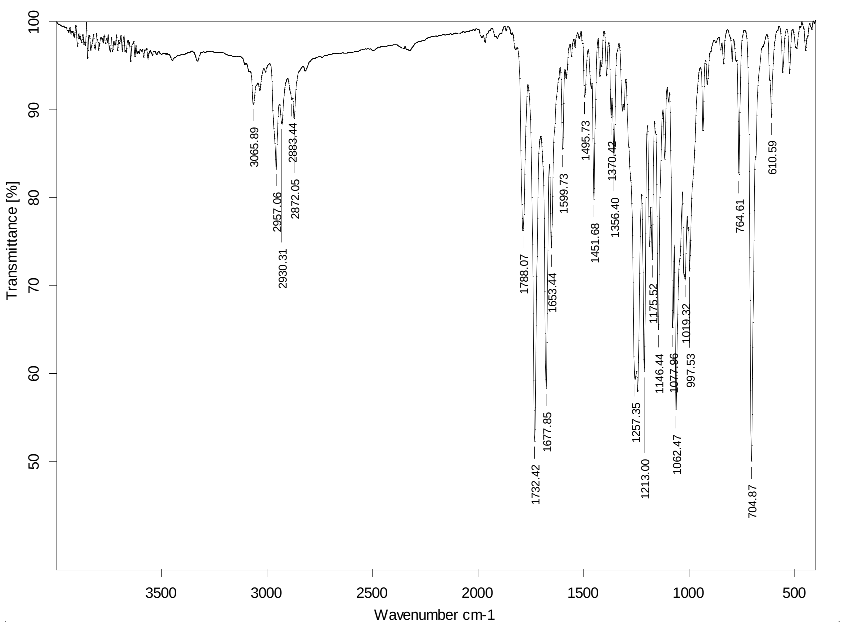

^{1}H NMR

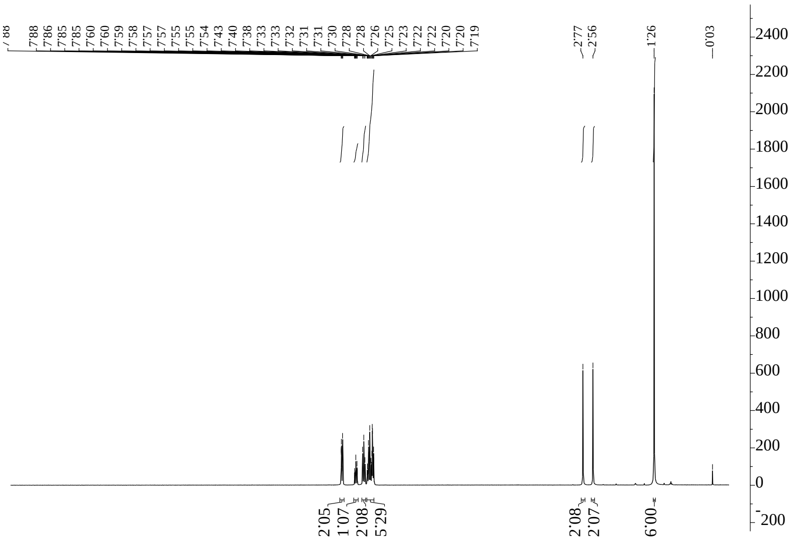

^{13}C NMR

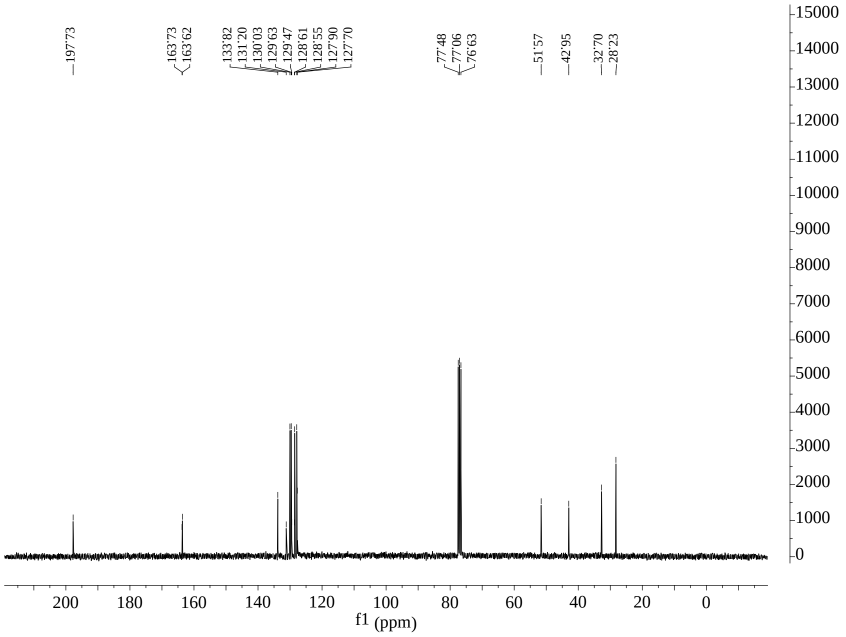
197.73
163.73
163.62
133.82
131.20
130.03
129.63
129.47
128.61
128.55
127.90
127.70
77.48
77.06
76.63
51.57
42.95
32.70
28.23
f1 (ppm)

HRMS

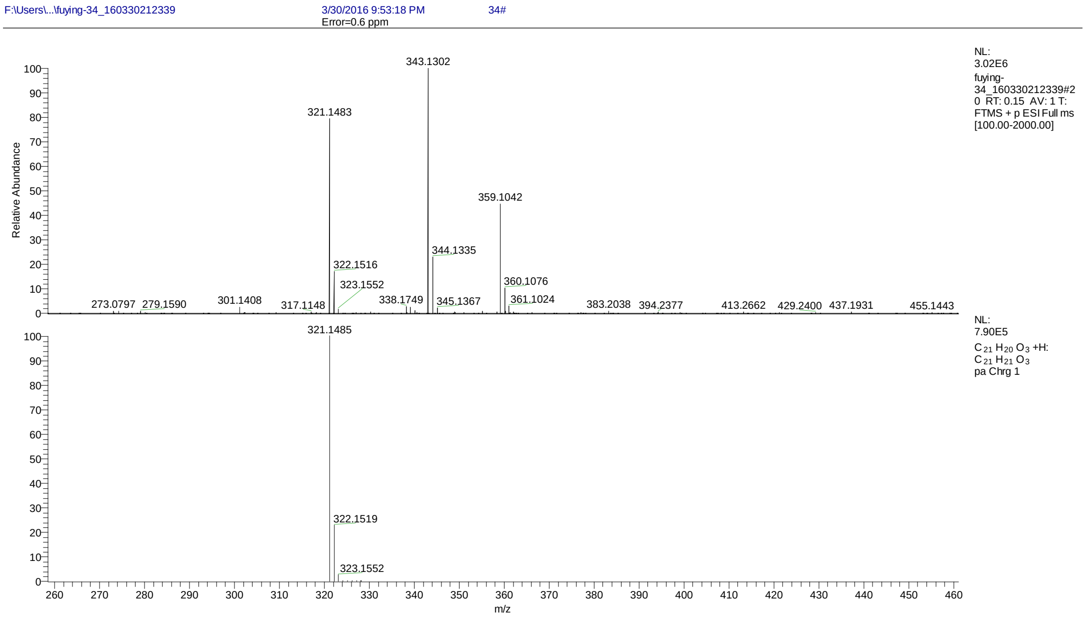
F:\Users\...\fuying-34_160330212339
3/30/2016 9:53:18 PM
34#
Error=0.6 ppm
NL: 3.02E6
fuying-34_160330212339#20 RT: 0.15 AV: 1 T: FTMS + p ESI Full ms [100.00-2000.00]
343.1302
321.1483
359.1042
344.1335
322.1516
323.1552
360.1076
361.1024
273.0797
279.1590
301.1408
317.1148
338.1749
345.1367
383.2038
394.2377
413.2662
429.2400
437.1931
455.1443
NL: 7.90E5
C 21 H 20 O 3 +H: C 21 H 21 O 3 pa Chrg 1
321.1485
322.1519
323.1552
Relative Abundance
m/z

3-(2,4-Dichlorobenzoyloxy)-2-phenyl-2-cyclohexen-1-one (S4)

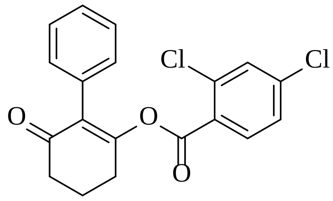

$C_{19}H_{14}Cl_2O_3$

IR

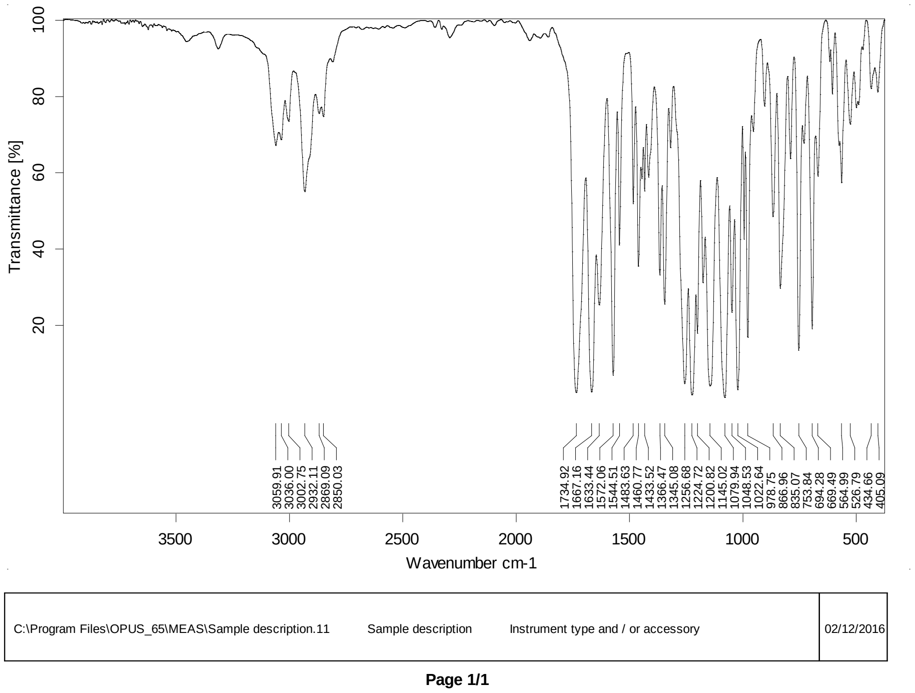

^{1}H NMR

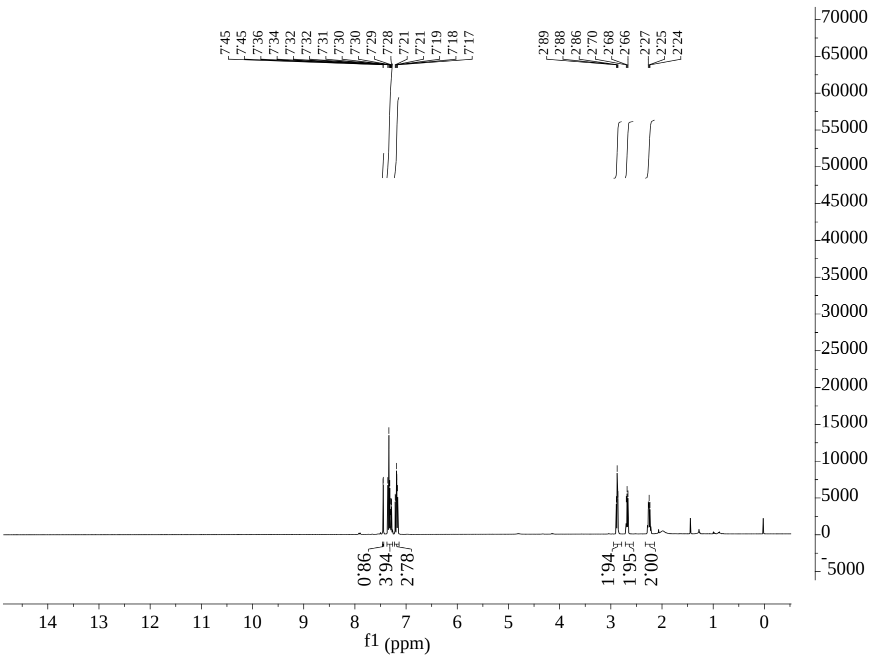

^{13}C NMR

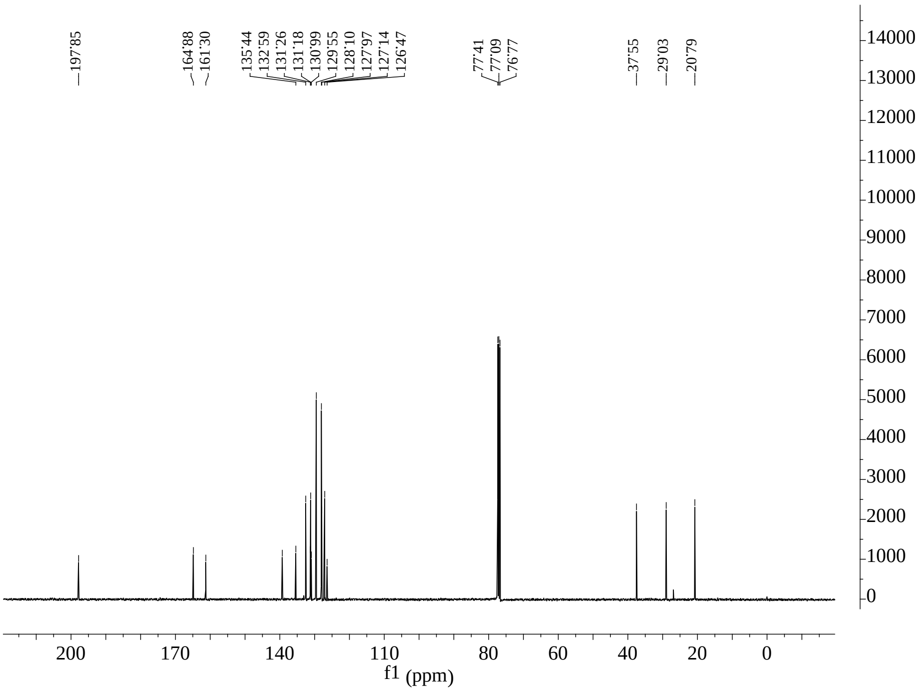

197.85
164.88
161.30
135.44
132.59
131.26
131.18
130.99
129.55
128.10
127.97
127.14
126.47
77.41
77.09
76.77
37.55
29.03
20.79
f1 (ppm)

HRMS

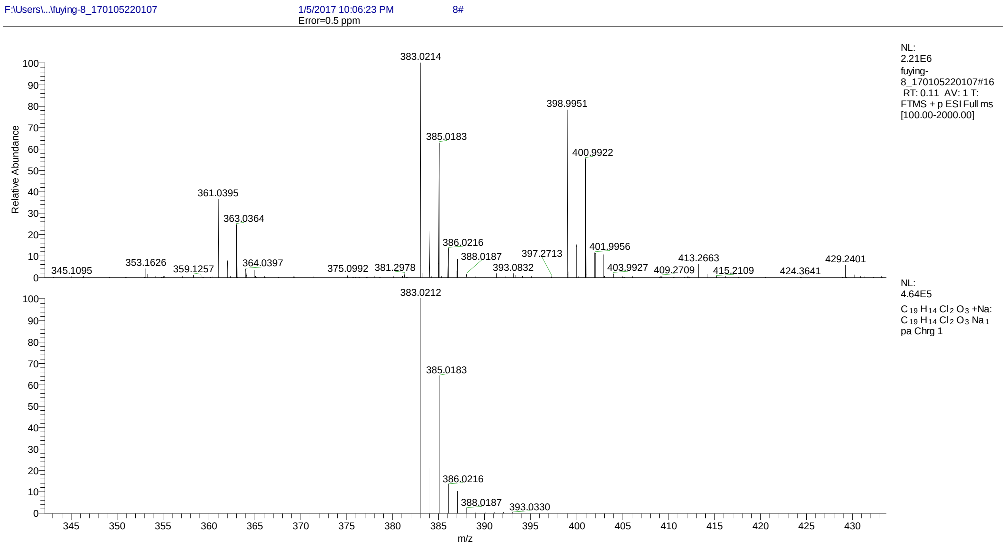

F:\Users\...\fuying-8_170105220107
1/5/2017 10:06:23 PM
Error=0.5 ppm
8#
NL: 2.21E6 fuying-8_170105220107#16 RT: 0.11 AV: 1 T: FTMS + p ESI Full ms [100.00-2000.00]
NL: 4.64E5 C 19 H 14 Cl 2 O 3 +Na: C 19 H 14 Cl 2 O 3 Na 1 pa Chrg 1
Relative Abundance
m/z
383.0214
398.9951
385.0183
400.9922
361.0395
363.0364
386.0216
401.9956
397.2713
388.0187
413.2663
429.2401
383.0212
393.0330

3-(2,4-Dichlorobenzoyloxy)-2-phenyl-5-methyl-2-cyclohexen-1-one (S5)

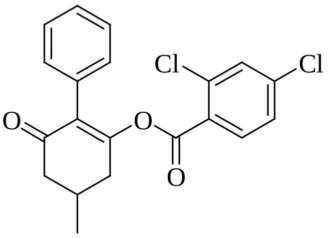

$C_{20}H_{16}Cl_2O_3$

IR

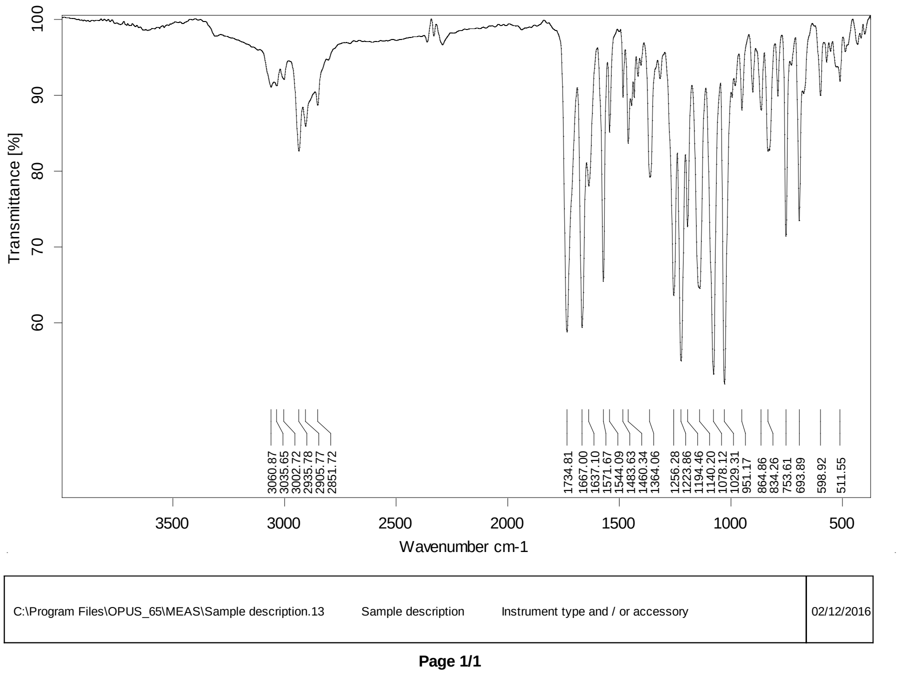

^{1}H NMR

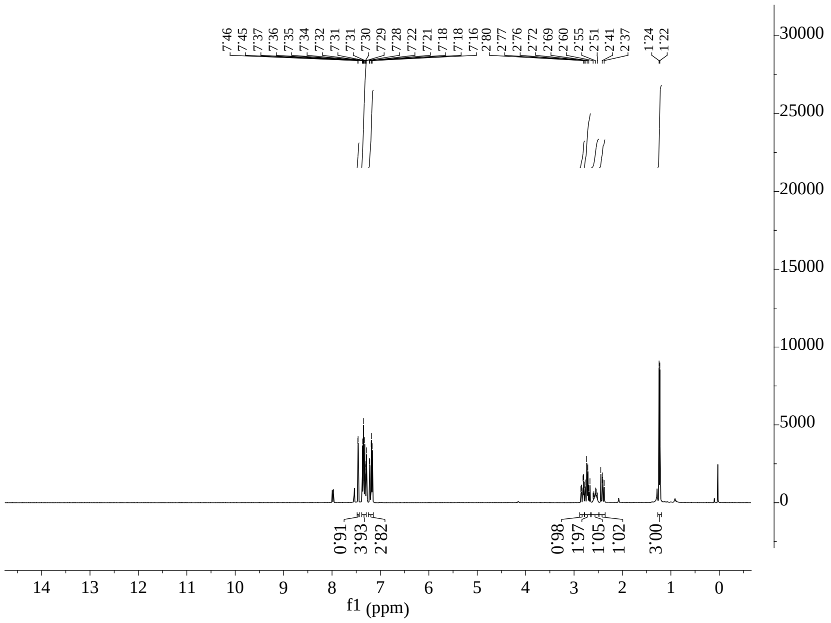

^{13}C NMR

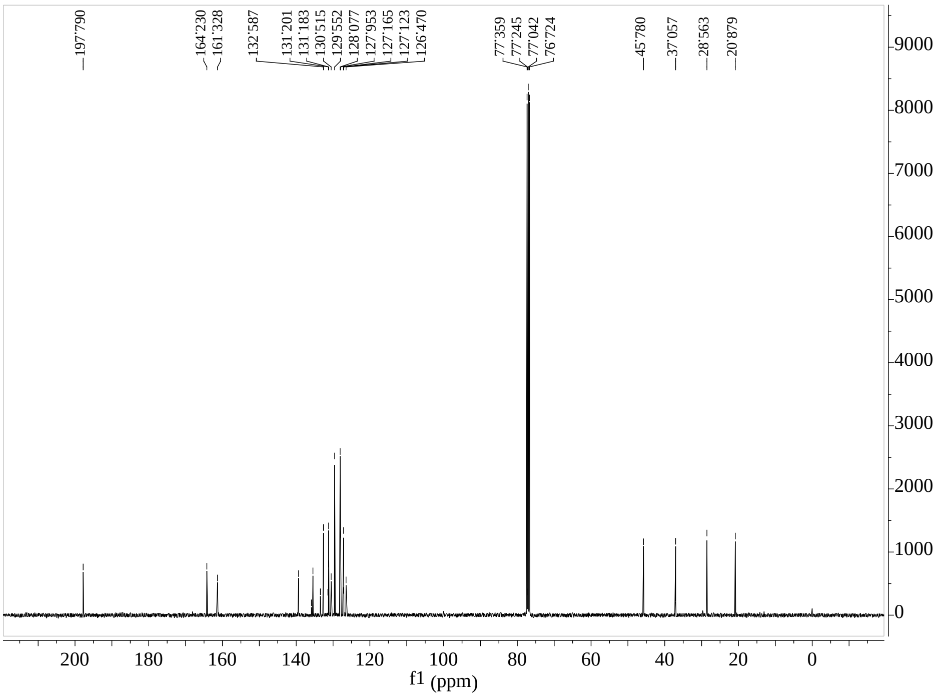
197.790
164.230
161.328
132.587
131.201
131.183
130.515
129.552
128.077
127.953
127.165
127.123
126.470
77.359
77.245
77.042
76.724
45.780
37.057
28.563
20.879
f1 (ppm)

HRMS

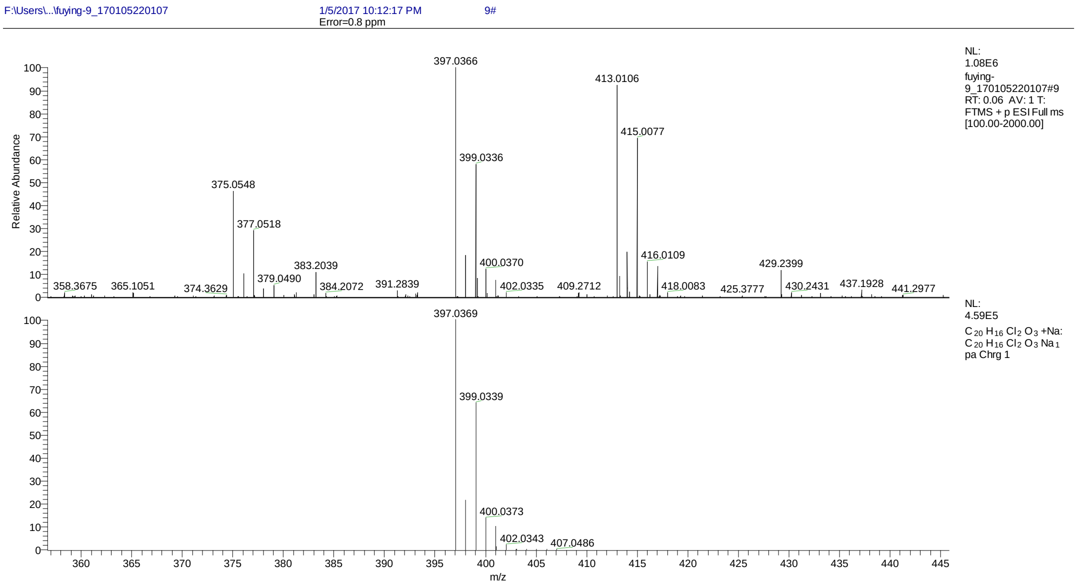
F:\Users\...\fuying-9_170105220107
1/5/2017 10:12:17 PM
Error=0.8 ppm
9#
NL: 1.08E6
fuying-9_170105220107#9 RT: 0.06 AV: 1 T: FTMS + p ESI Full ms [100.00-2000.00]
397.0366
413.0106
415.0077
399.0336
375.0548
377.0518
383.2039
400.0370
416.0109
429.2399
NL: 4.59E5
C 20 H 16 Cl 2 O 3 +Na: C 20 H 16 Cl 2 O 3 Na 1 pa Chrg 1
397.0369
399.0339
400.0373
402.0343
407.0486
Relative Abundance
m/z

3-(2,4-Dichlorobenzoyloxy)-2-phenyl-5,5-dimethyl-2-cyclohexen-1-one (S6):

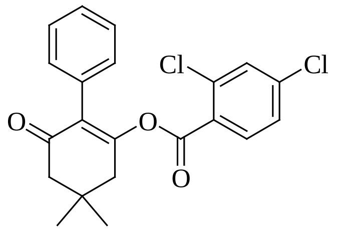

$C_{21}H_{18}Cl_2O_3$

IR

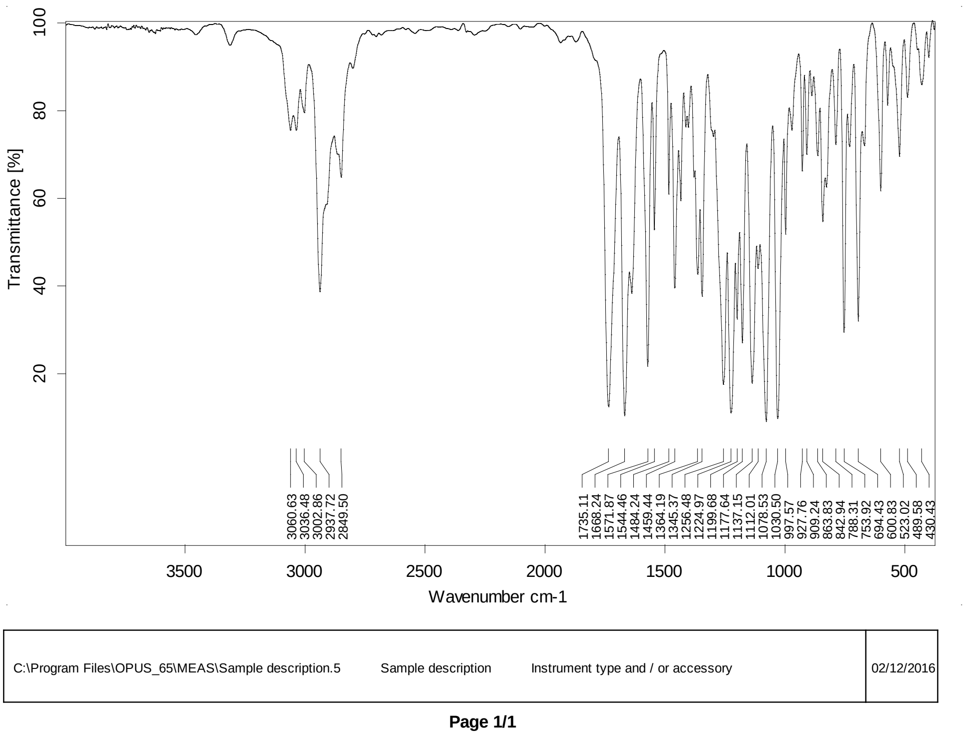

^{1}H NMR

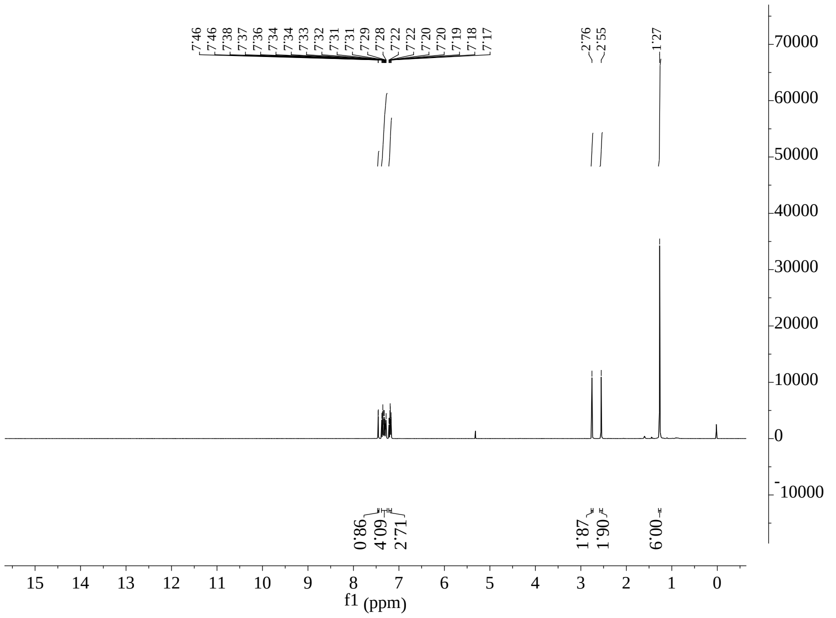

^{13}C NMR

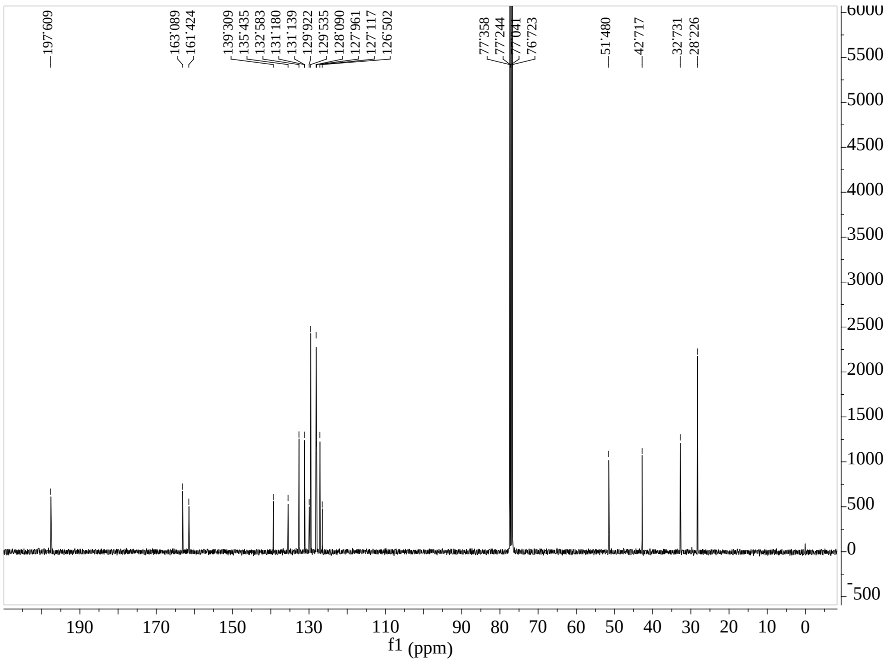
197.609
163.089
161.424
139.309
135.435
132.583
131.180
131.139
129.922
129.535
128.090
127.961
127.117
126.502
77.358
77.244
77.041
76.723
51.480
42.717
32.731
28.226
f1 (ppm)

HRMS

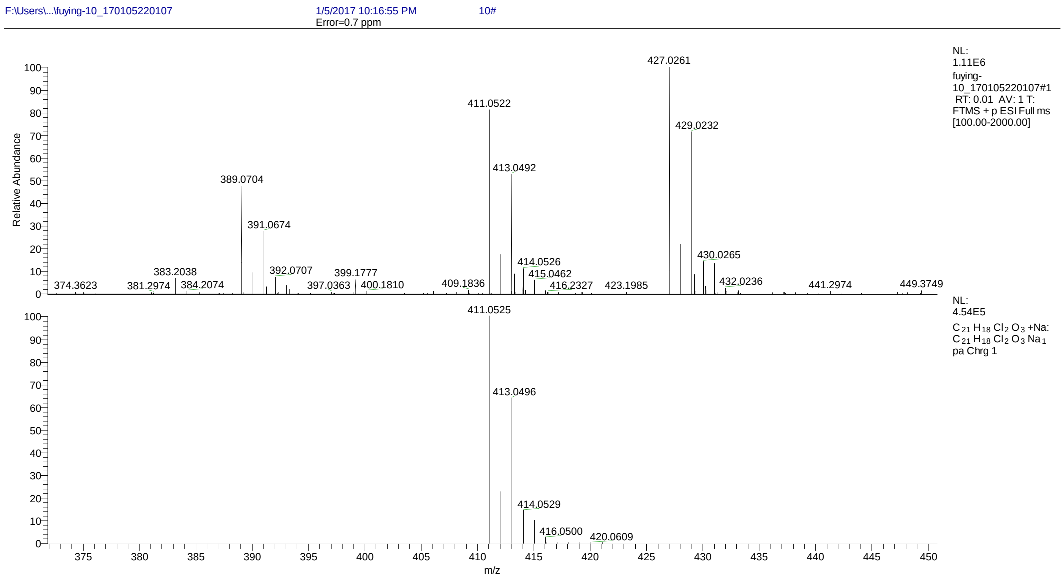
F:\Users\...\fuying-10_170105220107
1/5/2017 10:16:55 PM
10#
Error=0.7 ppm
NL: 1.11E6
fuying-10_170105220107#1 RT: 0.01 AV: 1 T: FTMS + p ESI Full ms [100.00-2000.00]
427.0261
411.0522
429.0232
413.0492
389.0704
391.0674
430.0265
414.0526
383.2038
392.0707
399.1777
415.0462
432.0236
374.3623
381.2974
384.2074
397.0363
400.1810
409.1836
416.2327
423.1985
441.2974
449.3749
Relative Abundance
NL: 4.54E5
C21 H18 Cl2 O3 +Na: C21 H18 Cl2 O3 Na1 pa Chrg 1
411.0525
413.0496
414.0529
416.0500
420.0609
m/z

3-(Chloroacetyloxy)-2-phenyl-5-methyl-2-cyclohexen-1-one (S7)

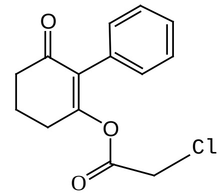

$C_{14}H_{13}ClO_3$

IR

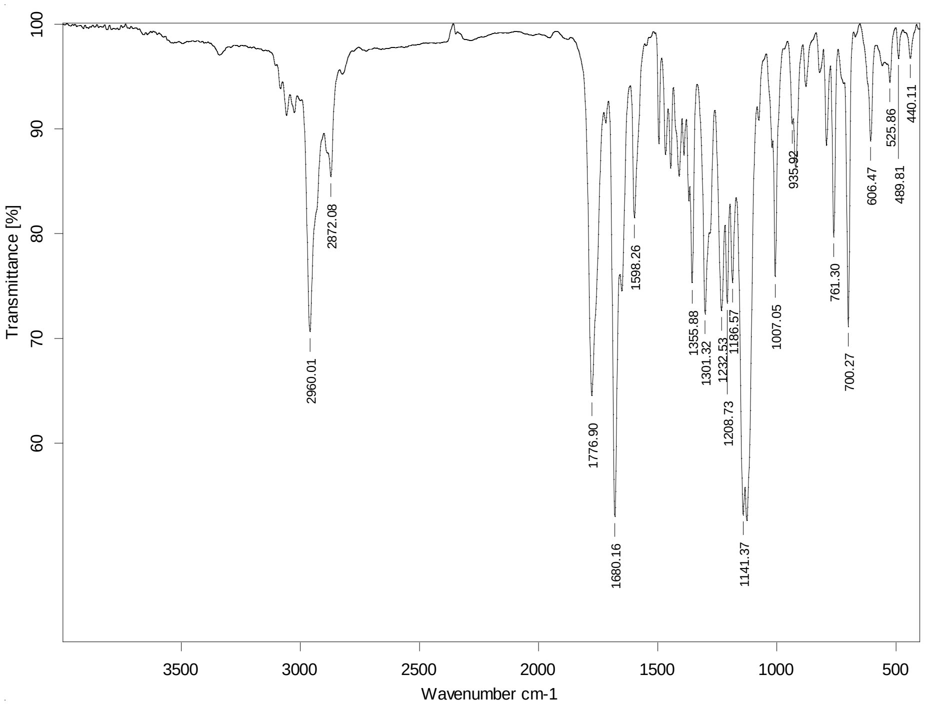

^{1}H NMR

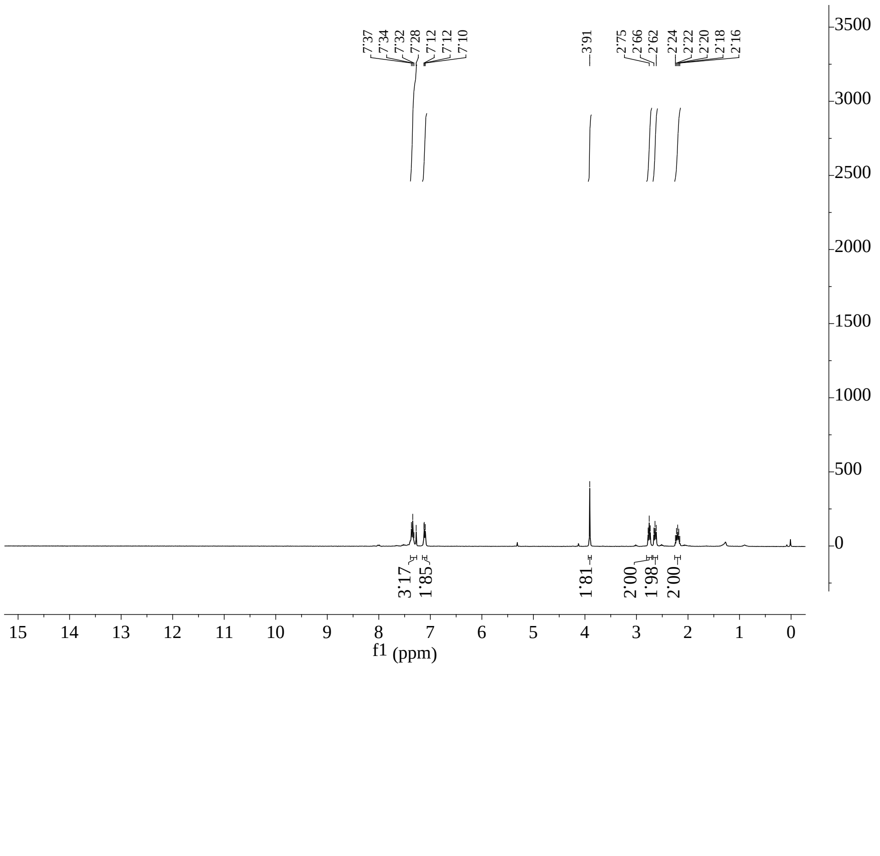

^{13}C NMR

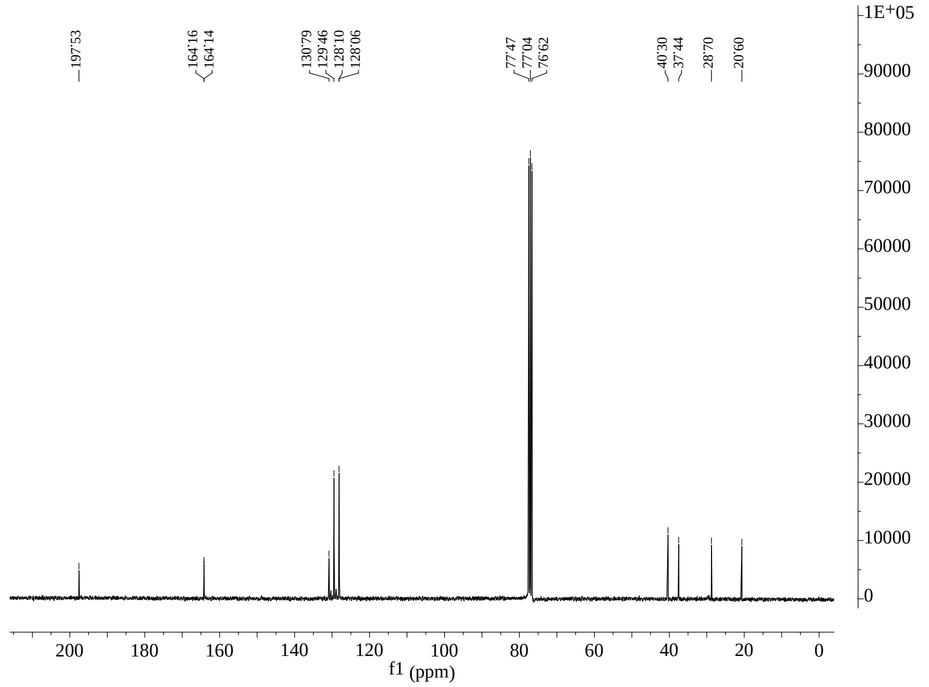
197.53
164.16
164.14
130.79
129.46
128.10
128.06
77.47
77.04
76.62
40.30
37.44
28.70
20.60
1E+05
90000
80000
70000
60000
50000
40000
30000
20000
10000
0
200
180
160
140
120
100
80
60
40
20
0
f1 (ppm)

HRMS

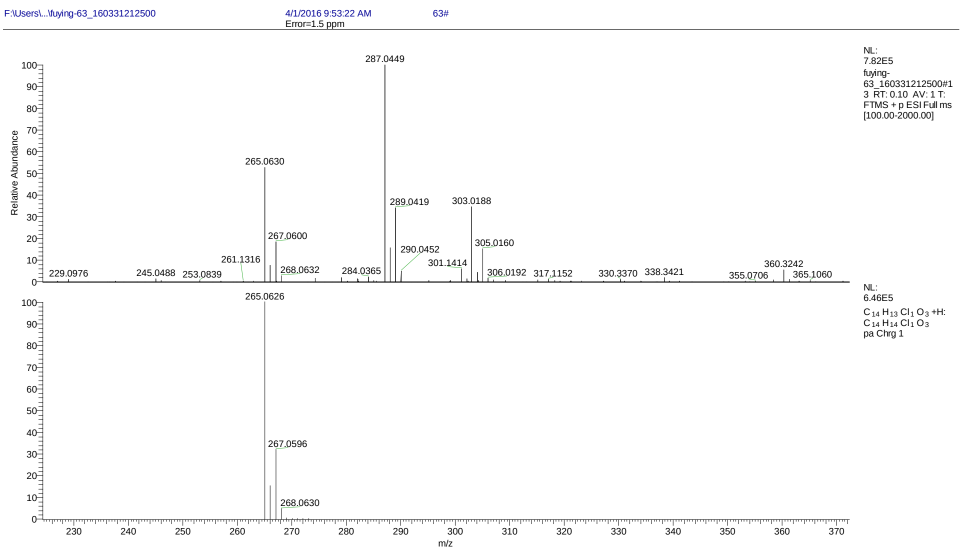
F:\Users\...\fuying-63_160331212500
4/1/2016 9:53:22 AM
Error=1.5 ppm
63#
NL: 7.82E5 fuying-63_160331212500#13 RT: 0.10 AV: 1 T: FTMS + p ESI Full ms [100.00-2000.00]
287.0449
265.0630
289.0419
303.0188
267.0600
305.0160
290.0452
261.1316
301.1414
360.3242
229.0976
245.0488
253.0839
268.0632
284.0365
306.0192
317.1152
330.3370
338.3421
355.0706
365.1060
Relative Abundance
NL: 6.46E5
C 14 H 13 Cl 1 O 3 +H: C 14 H 14 Cl 1 O 3 pa Chrg 1
265.0626
267.0596
268.0630
m/z

3-(Chloroacetyloxy)-2-phenyl-5-methyl-2-cyclohexen-1-one (S8)

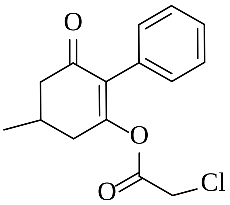

$C_{15}H_{15}ClO_3$

IR

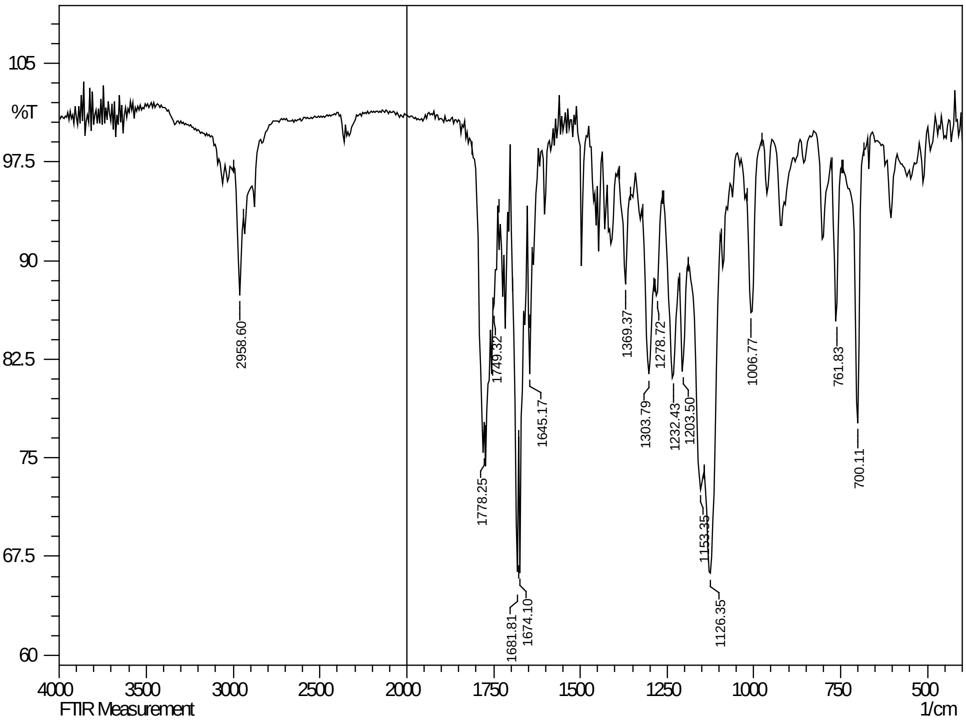

^{1}H NMR

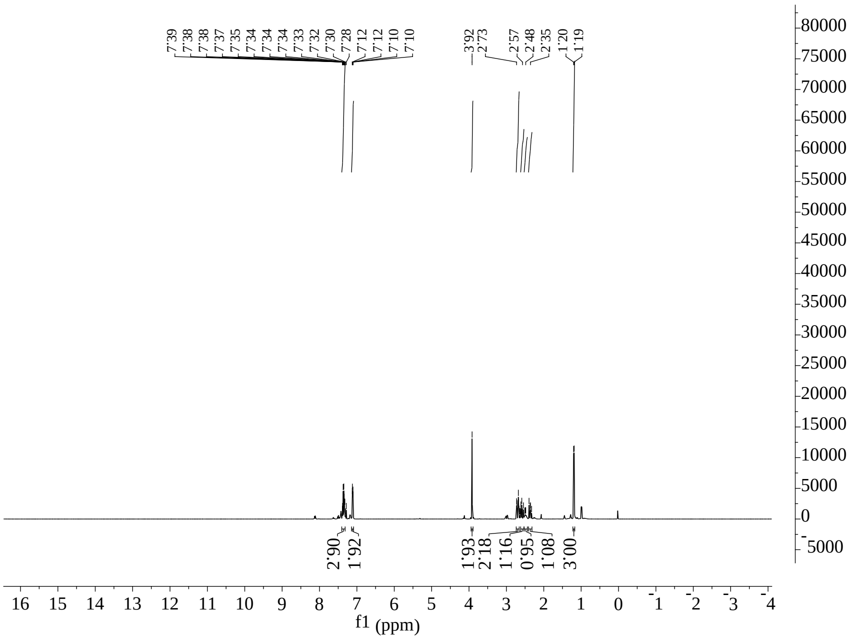

^{13}C NMR

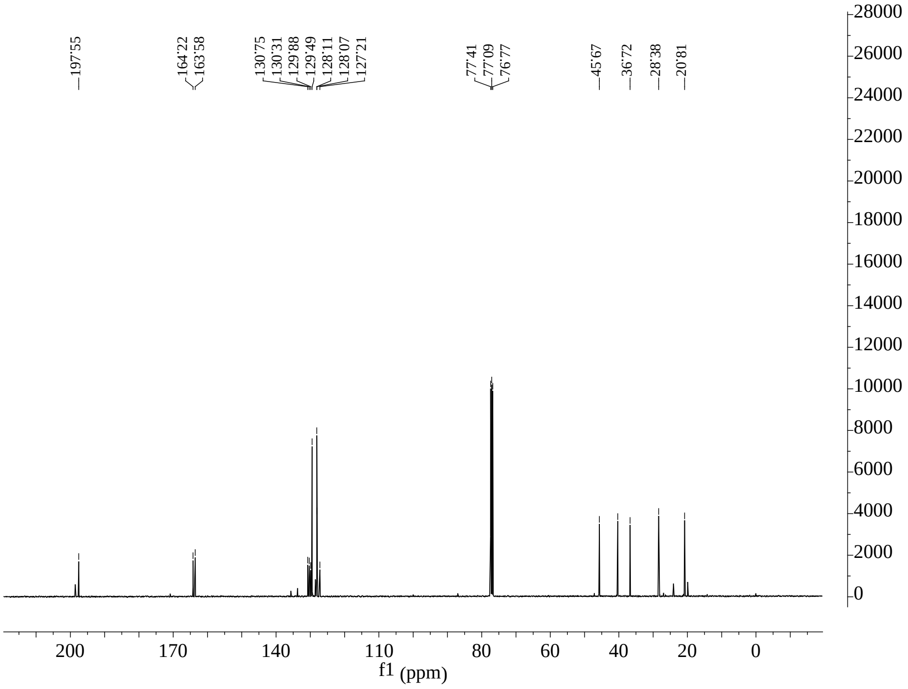

197.55
164.22
163.58
130.75
130.31
129.88
129.49
128.11
128.07
127.21
77.41
77.09
76.77
45.67
36.72
28.38
20.81
f1 (ppm)

HRMS

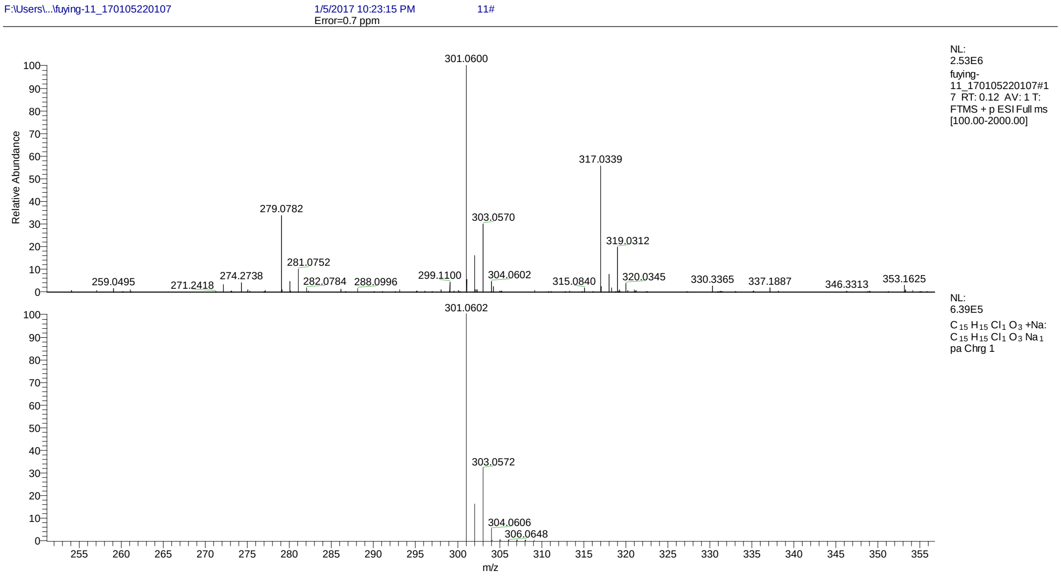

F:\Users\...\fuying-11_170105220107
1/5/2017 10:23:15 PM
11#
Error=0.7 ppm
NL: 2.53E6
fuying-11_170105220107#17 RT: 0.12 AV: 1 T: FTMS + p ESI Full ms [100.00-2000.00]
301.0600
317.0339
279.0782
303.0570
319.0312
281.0752
274.2738
304.0602
320.0345
259.0495
271.2418
282.0784
288.0996
299.1100
315.0840
330.3365
337.1887
346.3313
353.1625
NL: 6.39E5
C 15 H 15 Cl 1 O 3 +Na: C 15 H 15 Cl 1 O 3 Na 1 pa Chrg 1
301.0602
303.0572
304.0606
306.0648
Relative Abundance
m/z

3-(Chloroacetyloxy)-2-phenyl-5,5-dimethyl-2-cyclohexen-1-one (S9)

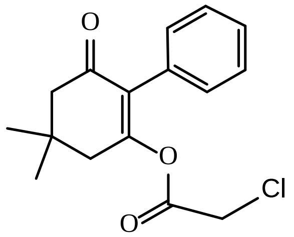

$C_{16}H_{17}ClO_3$

IR

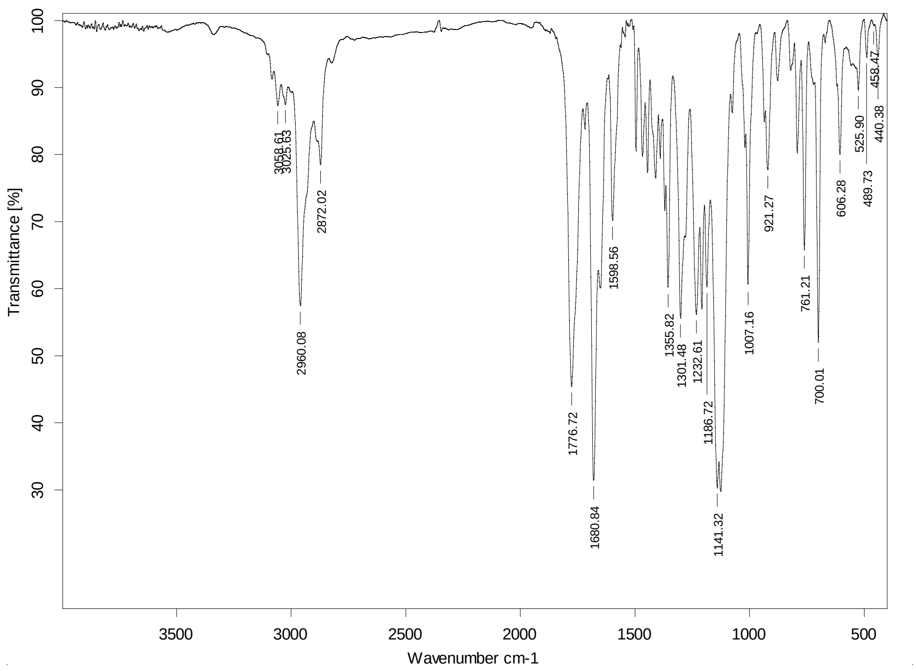

^{1}H NMR

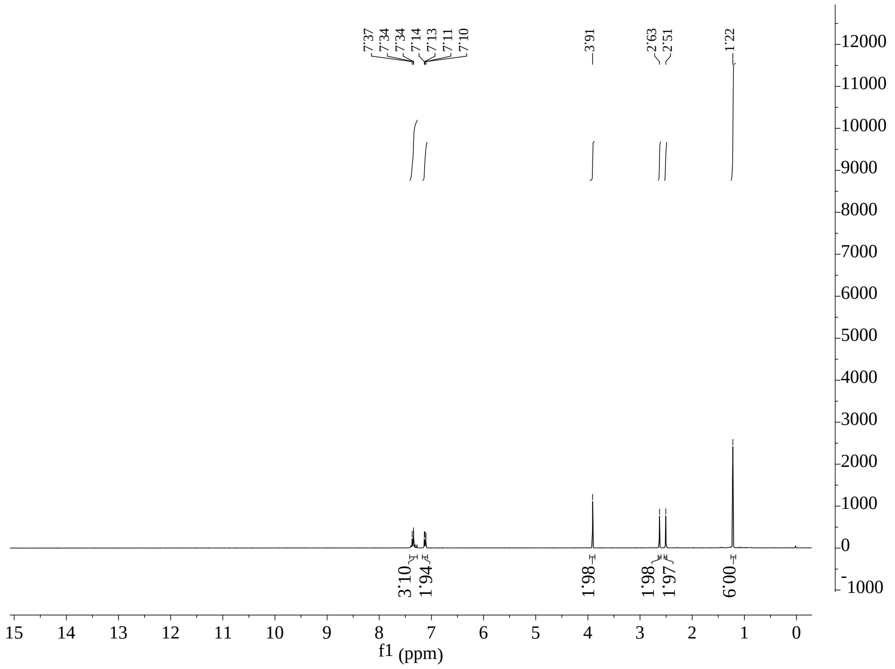

^{13}C NMR

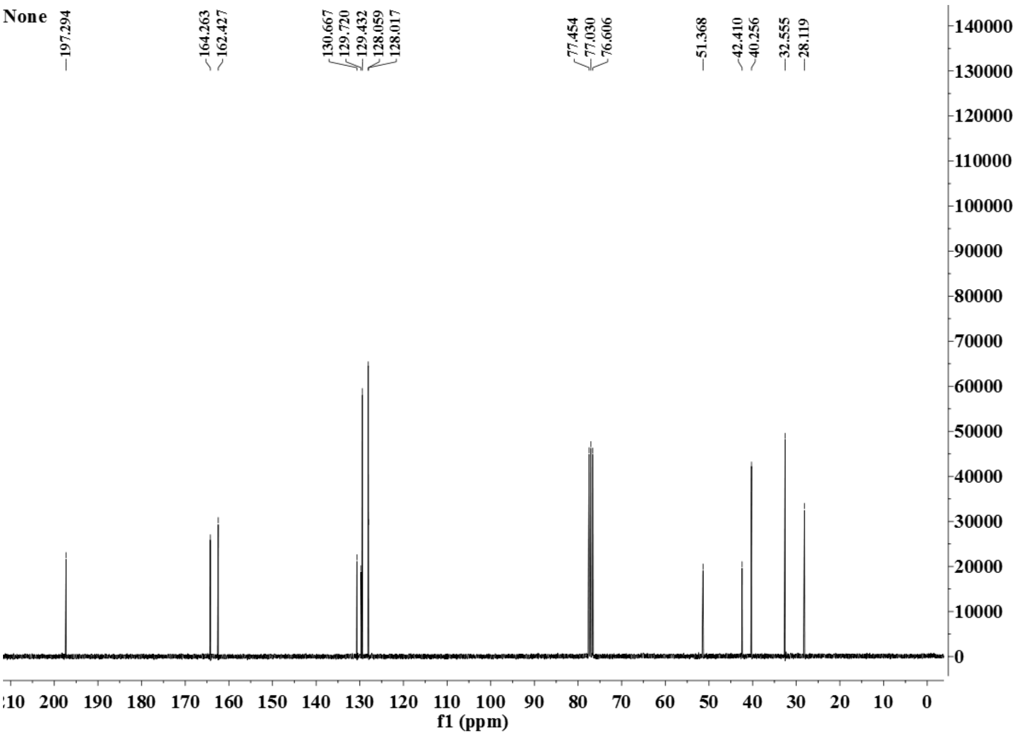
None
197.294
164.263
162.427
130.667
129.720
129.432
128.059
128.017
77.454
77.030
76.606
51.368
42.410
40.256
32.555
28.119
f1 (ppm)

HRMS

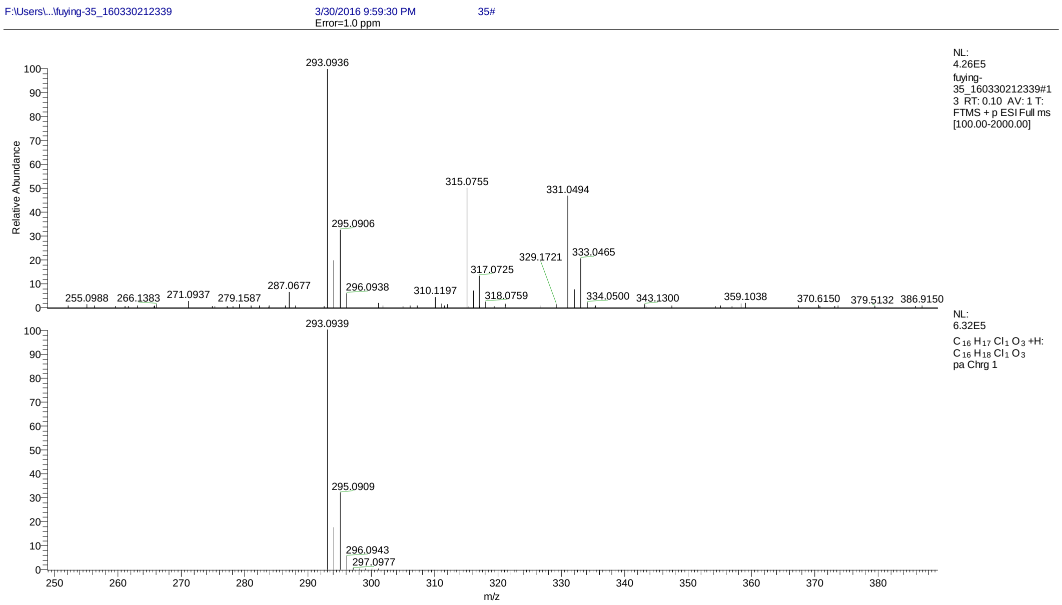
F:\Users\...\fuying-35_160330212339
3/30/2016 9:59:30 PM
Error=1.0 ppm
35#
NL: 4.26E5
fuying-35_160330212339#13 RT: 0.10 AV: 1 T: FTMS + p ESI Full ms [100.00-2000.00]
293.0936
315.0755
331.0494
295.0906
333.0465
329.1721
317.0725
NL: 6.32E5
C 16 H 17 Cl 1 O 3 +H: C 16 H 18 Cl 1 O 3 pa Chrg 1
293.0939
295.0909
296.0943
297.0977
Relative Abundance
m/z

3-(Dichloroacetyloxy)-2-phenyl-5,5-dimethyl-2-cyclohexen-1-one (S10)

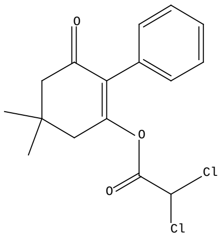

$C_{16}H_{16}Cl_2O_3$

IR

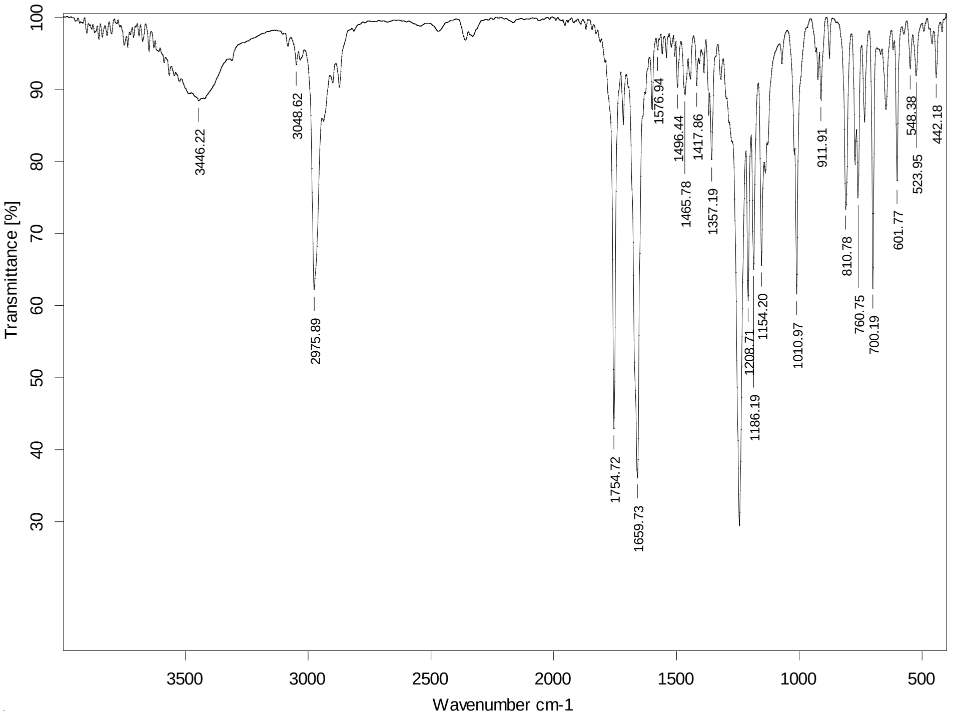

^{1}H NMR

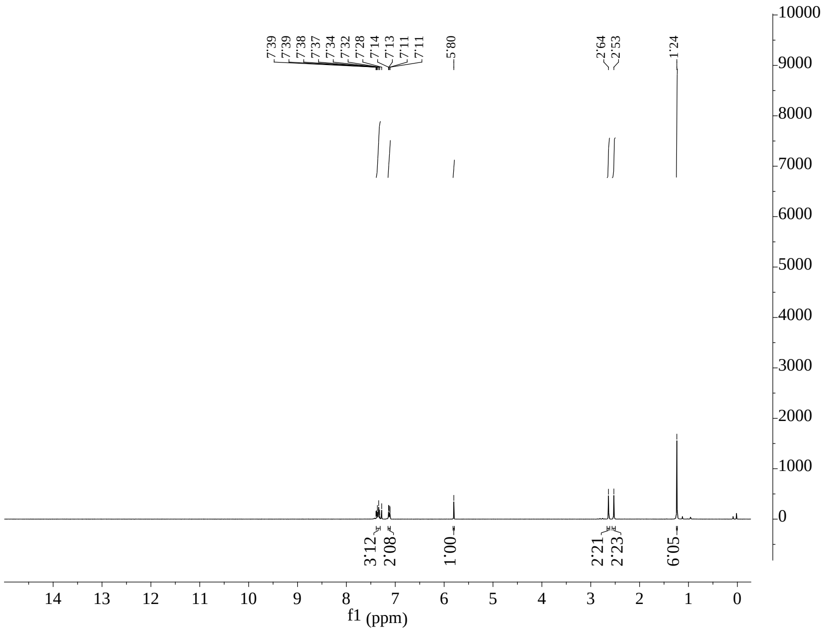

^{13}C NMR

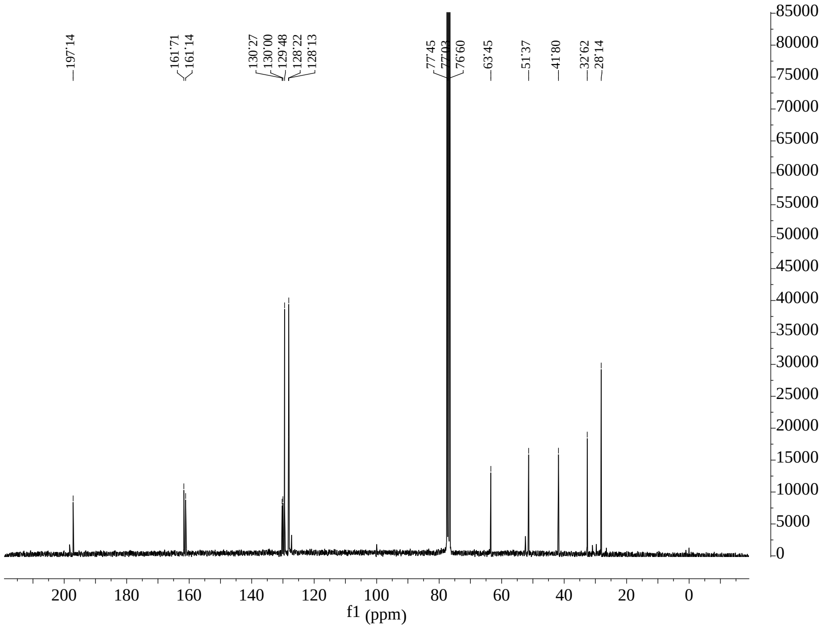
197.14
161.71
161.14
130.27
130.00
129.48
128.22
128.13
77.45
77.03
76.60
63.45
51.37
41.80
32.62
28.14
f1 (ppm)

HRMS

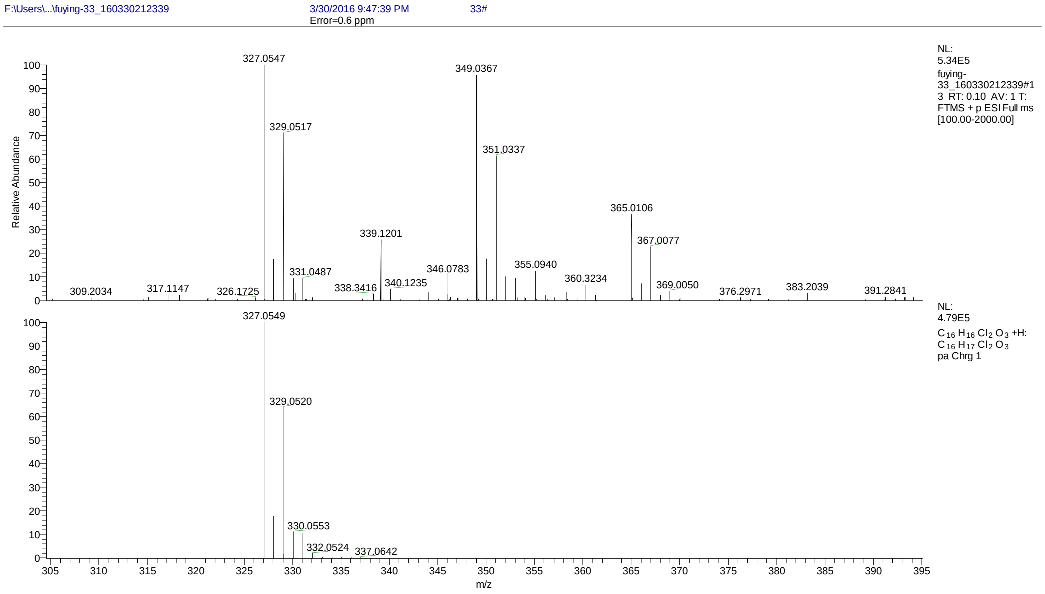
F:\Users\...\fuying-33_160330212339
3/30/2016 9:47:39 PM
Error=0.6 ppm
33#
NL: 5.34E5
fuying-33_160330212339#13 RT: 0.10 AV: 1 T: FTMS + p ESI Full ms [100.00-2000.00]
327.0547
349.0367
329.0517
351.0337
365.0106
367.0077
339.1201
331.0487
346.0783
355.0940
360.3234
340.1235
338.3416
369.0050
376.2971
383.2039
391.2841
309.2034
317.1147
326.1725
NL: 4.79E5
C 16 H 16 Cl 2 O 3 +H: C 16 H 17 Cl 2 O 3 pa Chrg 1
327.0549
329.0520
330.0553
332.0524
337.0642
Relative Abundance
m/z

3-(Phenoxyacetyloxy)-2-phenyl-2-cyclohexen-1-one (S11)

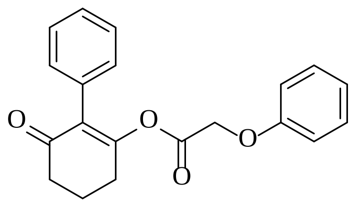

$C_{20}H_{18}O_4$

IR

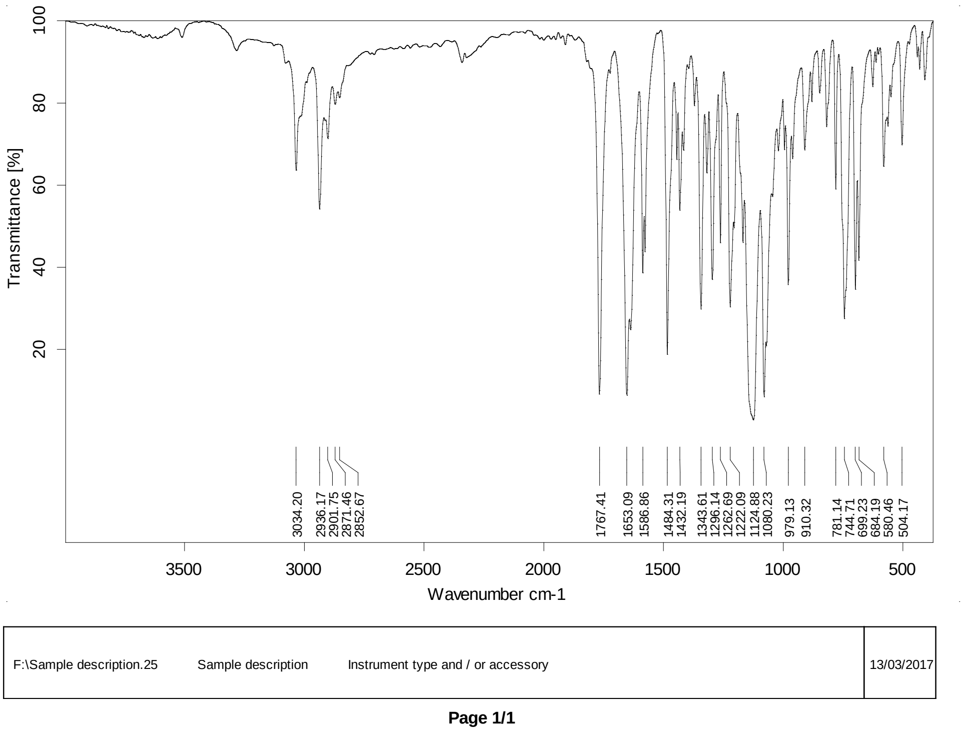

^{1}H NMR

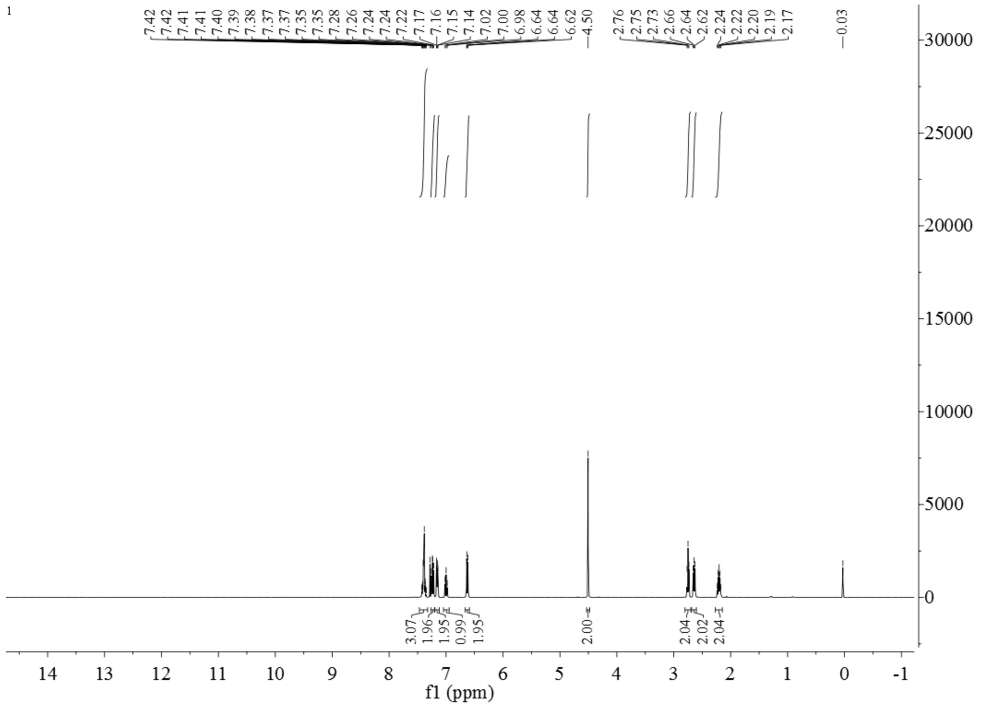

^{13}C NMR

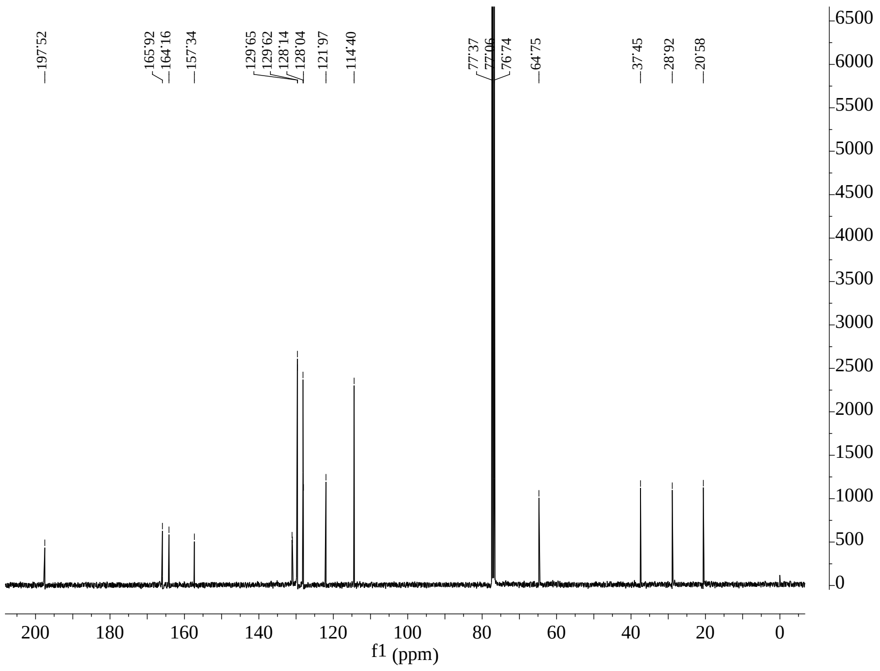
197.52
165.92
164.16
157.34
129.65
129.62
128.14
128.04
121.97
114.40
77.37
77.05
76.74
64.75
37.45
28.92
20.58
f1 (ppm)

HRMS

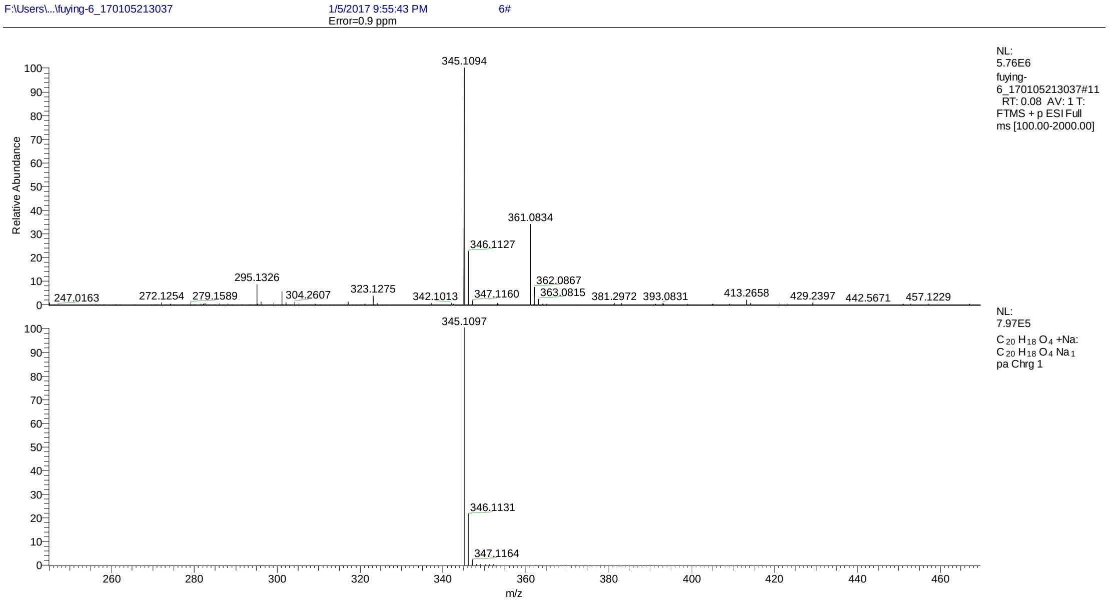
F:\Users\...\fuying-6_170105213037
1/5/2017 9:55:43 PM
6#
Error=0.9 ppm
NL: 5.76E6
fuying-6_170105213037#11 RT: 0.08 AV: 1 T: FTMS + p ESI Full ms [100.00-2000.00]
NL: 7.97E5
C 20 H 18 O 4 +Na: C 20 H 18 O 4 Na 1 pa Chrg 1
Relative Abundance
m/z
345.1094
361.0834
346.1127
295.1326
323.1275
362.0867
363.0815
345.1097
346.1131
347.1164

3-(Phenoxyacetyloxy)-2-phenyl-5-methyl-2-cyclohexen-1-one (S12)

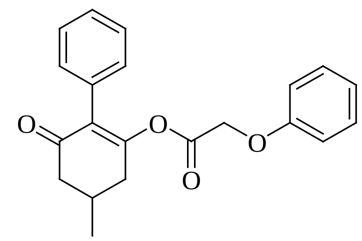

$C_{21}H_{20}O_4$

IR

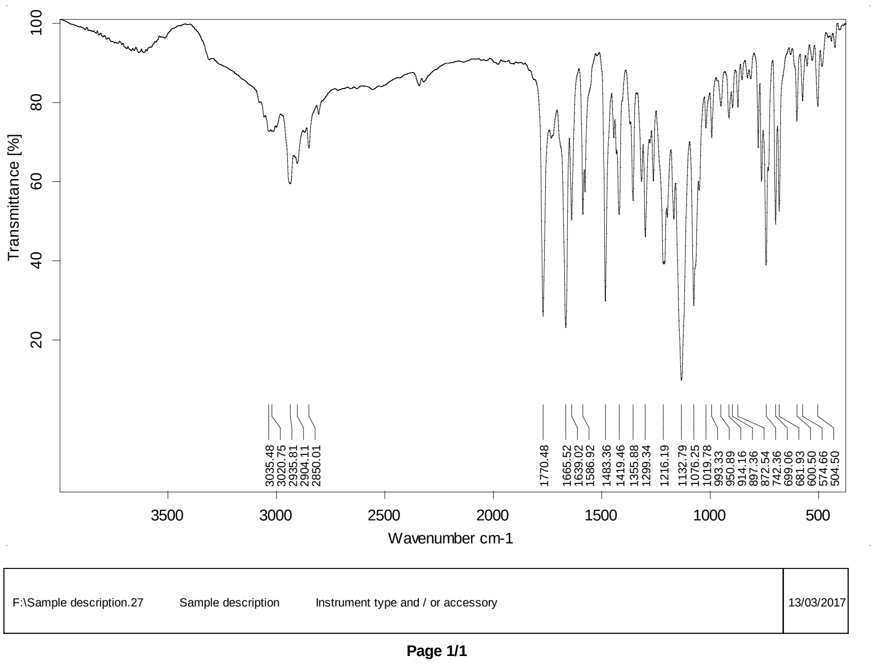

^{1}H NMR

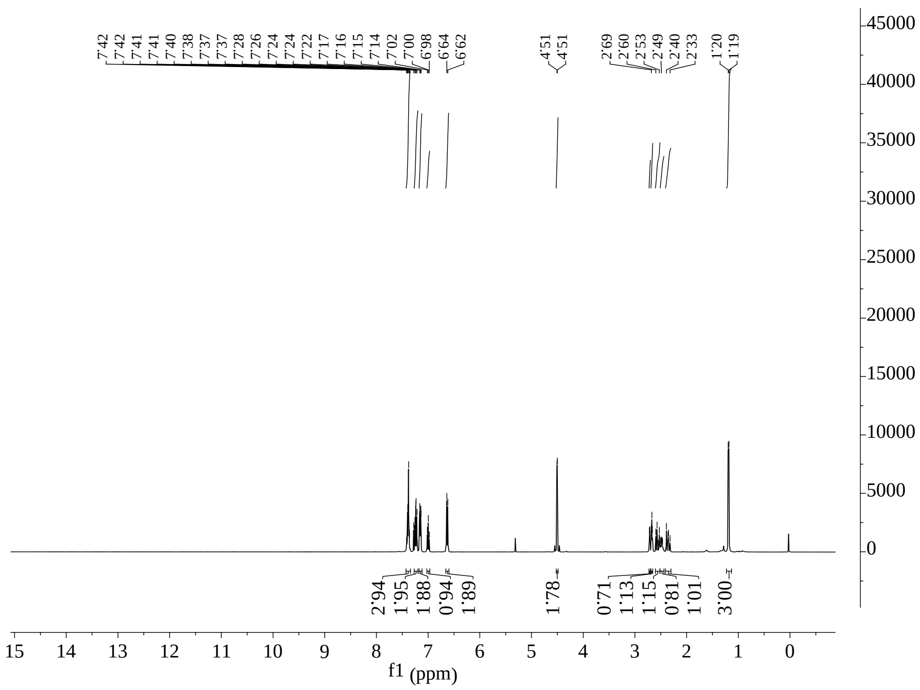

^{13}C NMR

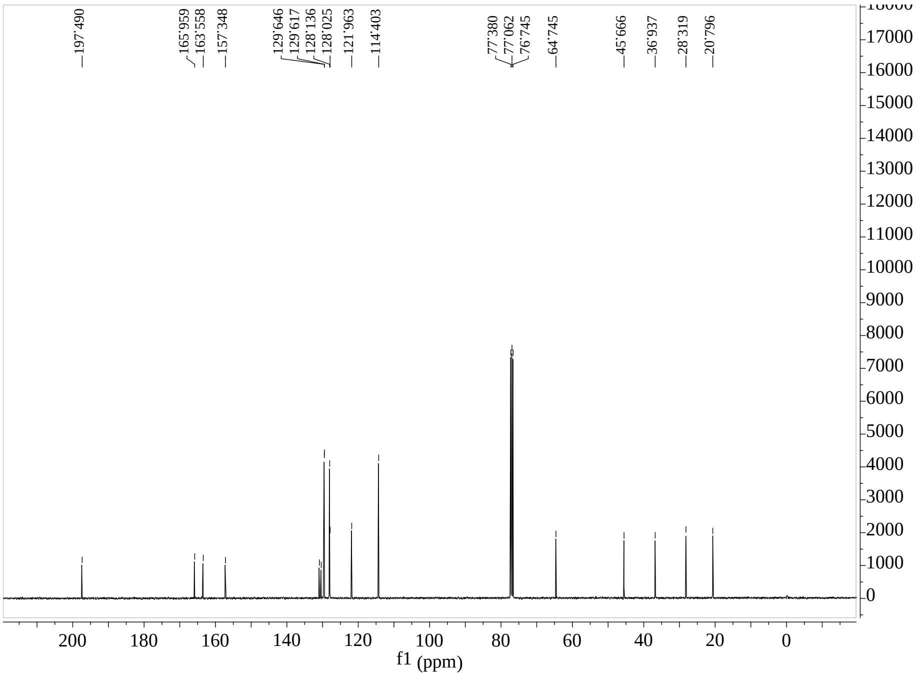
197.490
165.959
163.558
157.348
129.646
129.617
128.136
128.025
121.963
114.403
77.380
77.062
76.745
64.745
45.666
36.937
28.319
20.796
f1 (ppm)

HRMS

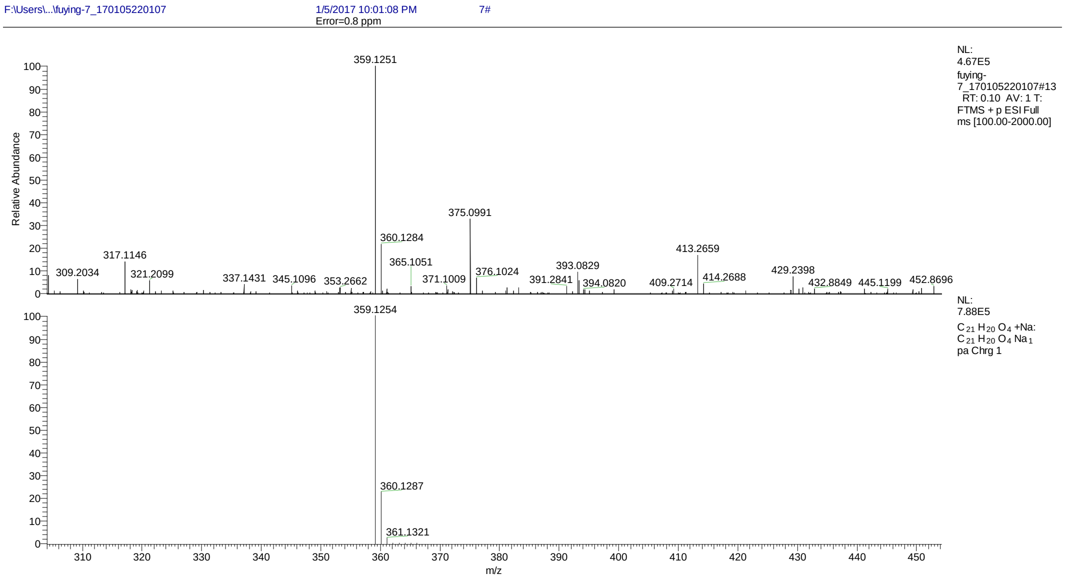
F:\Users\...\fuying-7_170105220107
1/5/2017 10:01:08 PM
Error=0.8 ppm
7#
NL: 4.67E5
fuying-7_170105220107#13 RT: 0.10 AV: 1 T: FTMS + p ESI Full ms [100.00-2000.00]
359.1251
375.0991
360.1284
413.2659
317.1146
365.1051
393.0829
429.2398
NL: 7.88E5
C 21 H 20 O 4 +Na: C 21 H 20 O 4 Na 1 pa Chrg 1
359.1254
360.1287
361.1321
Relative Abundance
m/z

3-(Phenoxyacetyloxy)-2-phenyl-5,5-dimethyl-2-cyclohexen-1-one (S13)

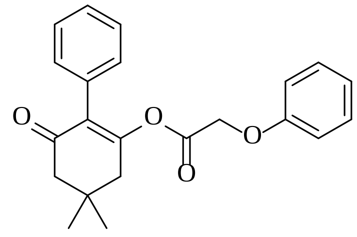

$C_{22}H_{22}O_4$

IR

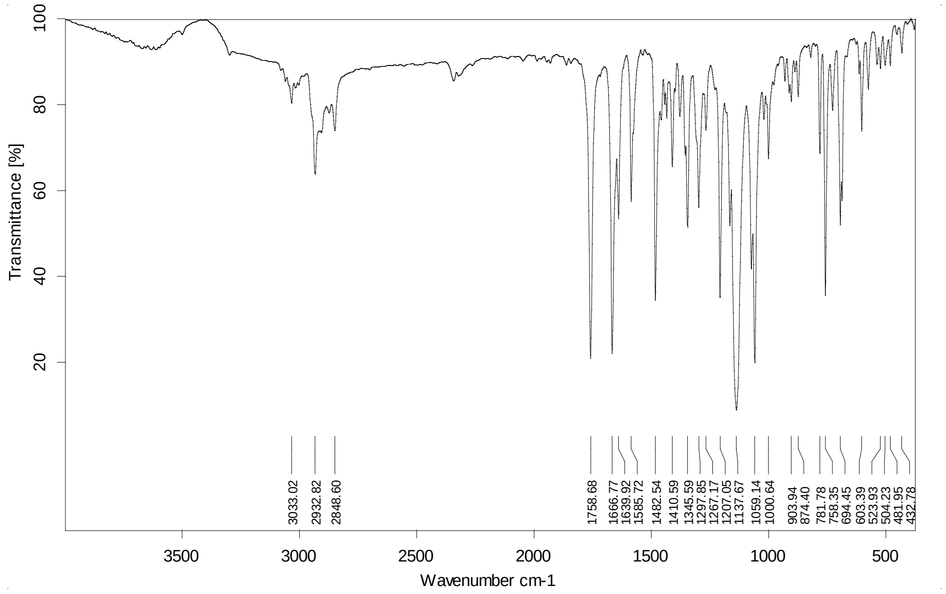

F:\Sample description.26	Sample description	Instrument type and / or accessory	13/03/2017

Page 1/1

^{1}H NMR

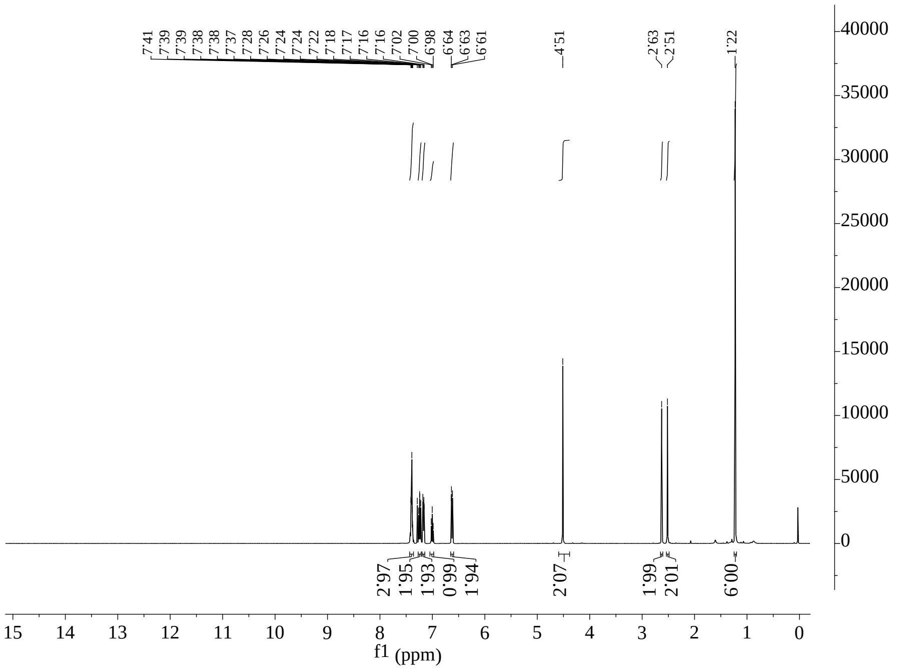

^{13}C NMR

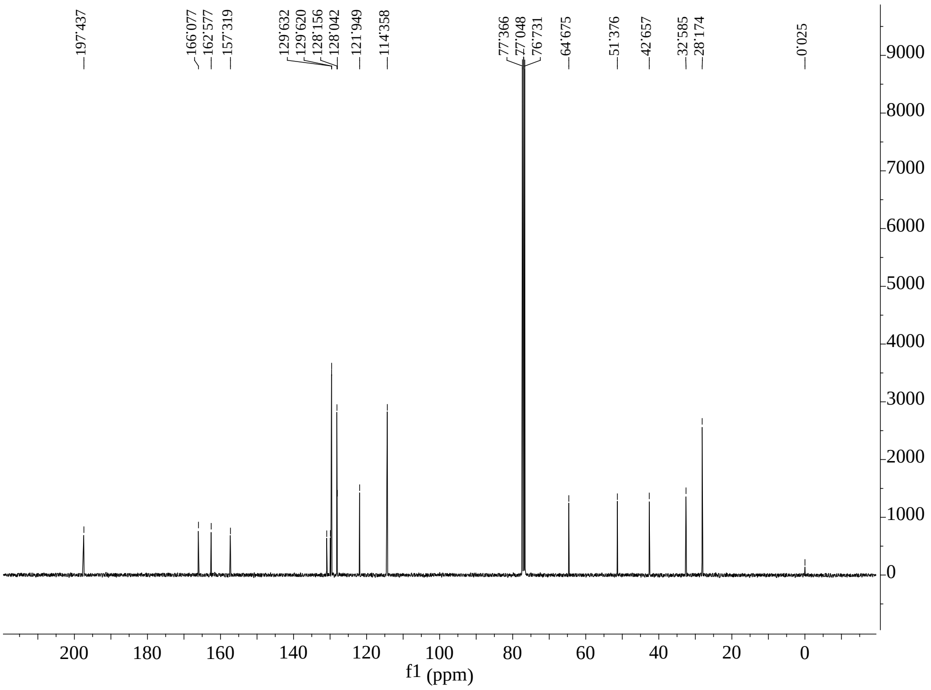

197.437
166.077
162.577
157.319
129.632
129.620
128.156
128.042
121.949
114.358
77.366
77.048
76.731
64.675
51.376
42.657
32.585
28.174
0.025
f1 (ppm)

HRMS

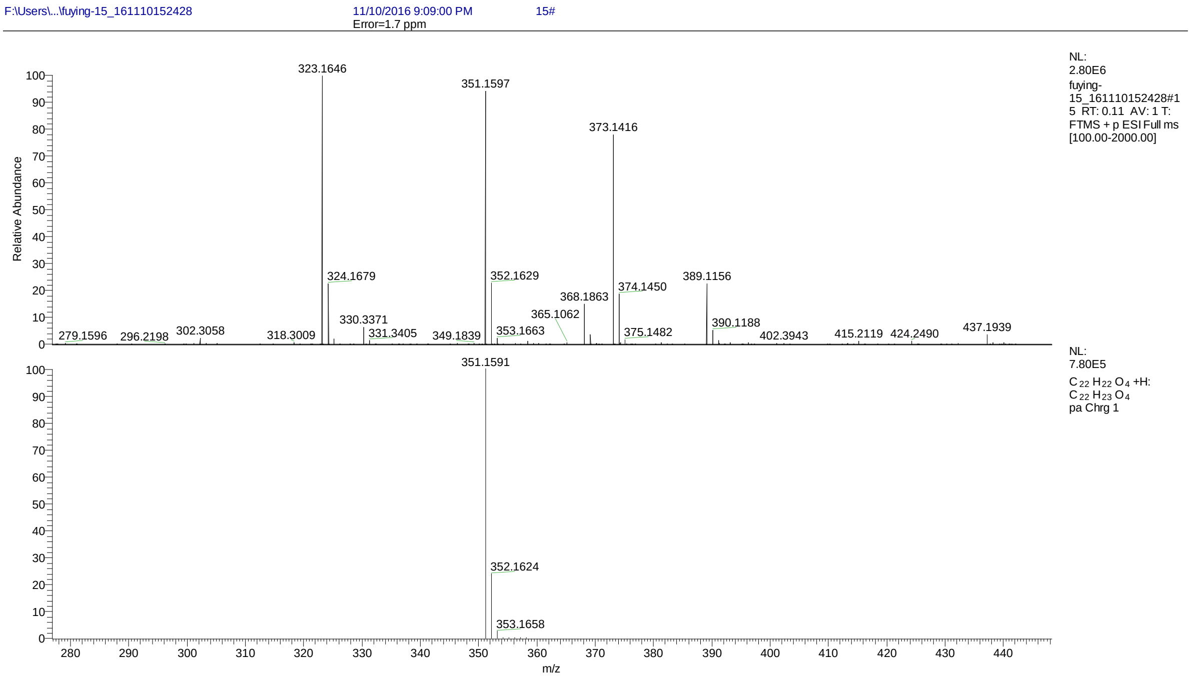

F:\Users\...\fuying-15_161110152428
11/10/2016 9:09:00 PM
15#
Error=1.7 ppm
NL: 2.80E6
fuying-15_161110152428#15 RT: 0.11 AV: 1 T: FTMS + p ESI Full ms [100.00-2000.00]
Relative Abundance
323.1646
351.1597
373.1416
324.1679
352.1629
374.1450
389.1156
368.1863
365.1062
330.3371
331.3405
349.1839
353.1663
375.1482
390.1188
402.3943
415.2119
424.2490
437.1939
279.1596
296.2198
302.3058
318.3009
NL: 7.80E5
C 22 H 22 O 4 +H:
C 22 H 23 O 4
pa Chrg 1
351.1591
352.1624
353.1658
m/z

3-(3-Acetylpropionoxy)-2-phenyl-2-cyclohexen-1-one (S14)

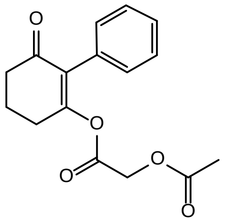

$C_{16}H_{16}O_5$

IR

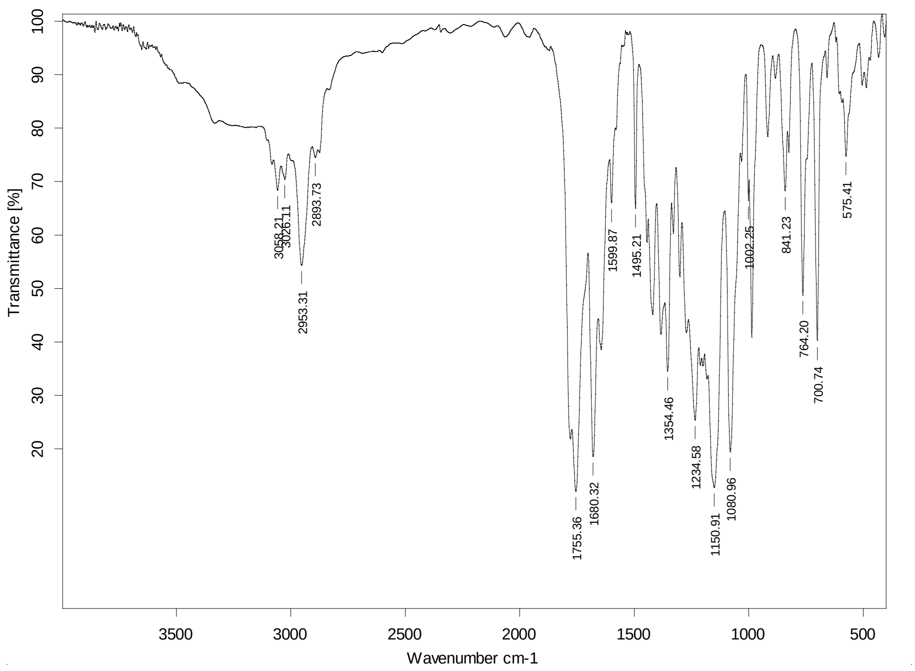

^{1}H NMR

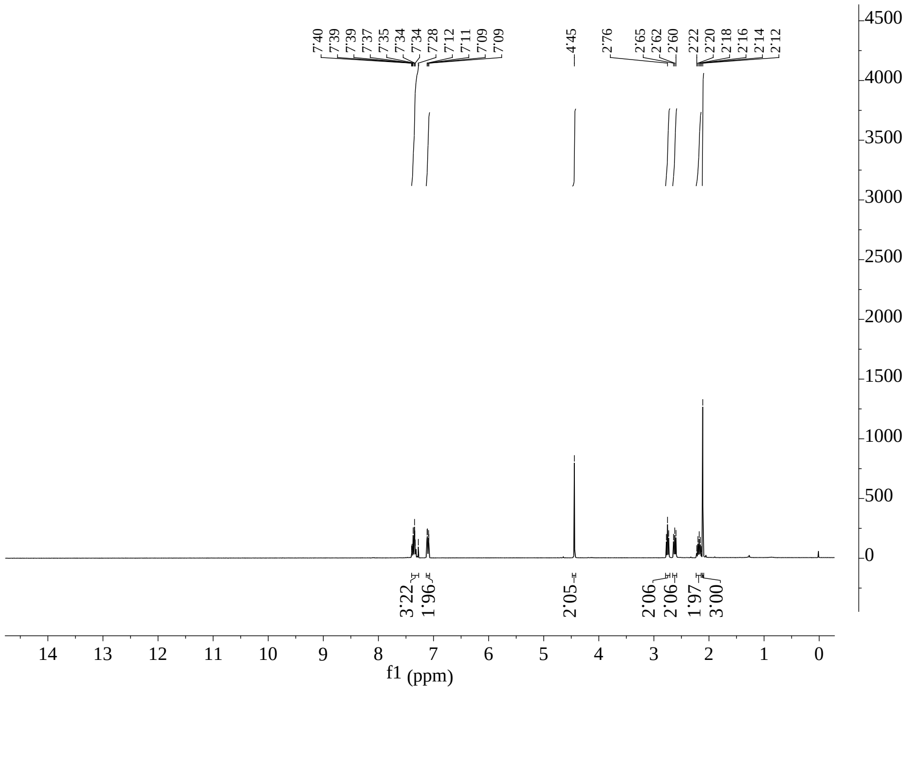

^{13}C NMR

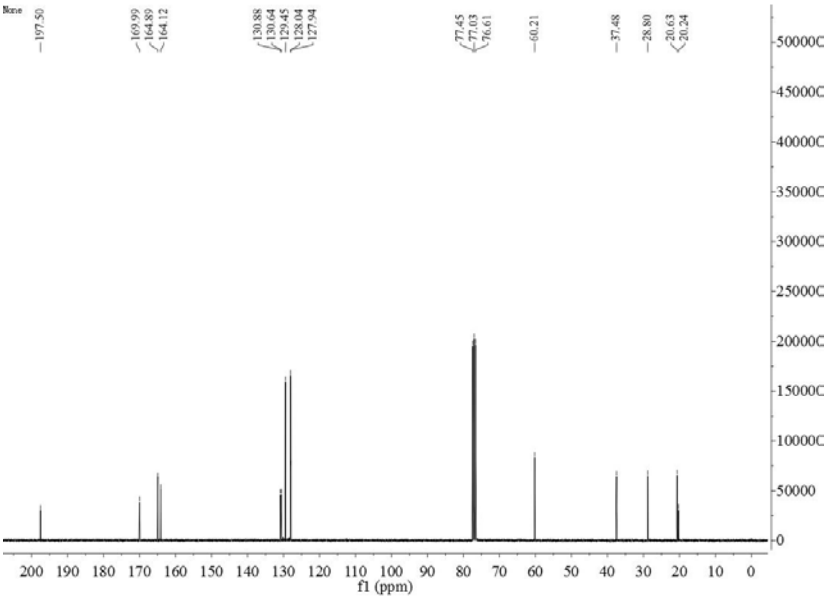
None
197.50
169.99
164.89
164.12
130.88
130.64
129.45
128.04
127.94
77.45
77.03
76.61
60.21
37.48
28.80
20.63
20.24
f1 (ppm)

HRMS

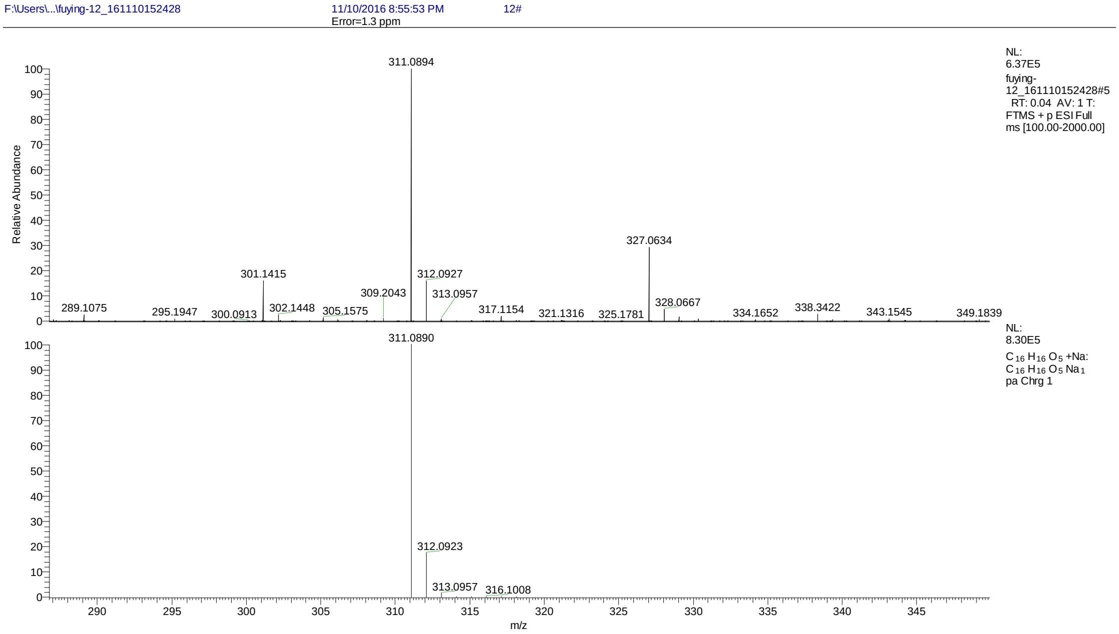
F:\Users\...\fuying-12_161110152428
11/10/2016 8:55:53 PM
12#
Error=1.3 ppm
NL: 6.37E5
fuying-12_161110152428#5 RT: 0.04 AV: 1 T: FTMS + p ESI Full ms [100.00-2000.00]
311.0894
327.0634
301.1415
312.0927
309.2043
313.0957
289.1075
295.1947
300.0913
302.1448
305.1575
317.1154
321.1316
325.1781
328.0667
334.1652
338.3422
343.1545
349.1839
NL: 8.30E5
C 16 H 16 O 5 +Na: C 16 H 16 O 5 Na 1 pa Chrg 1
311.0890
312.0923
313.0957
316.1008
Relative Abundance
m/z

3-(3-Acetylpropionoxy)-2-phenyl-5-methyl-2-cyclohexen-1-one (S15)

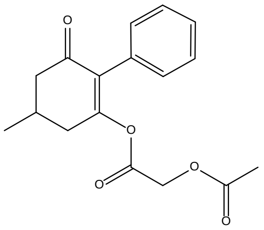

$C_{17}H_{18}O_5$

IR

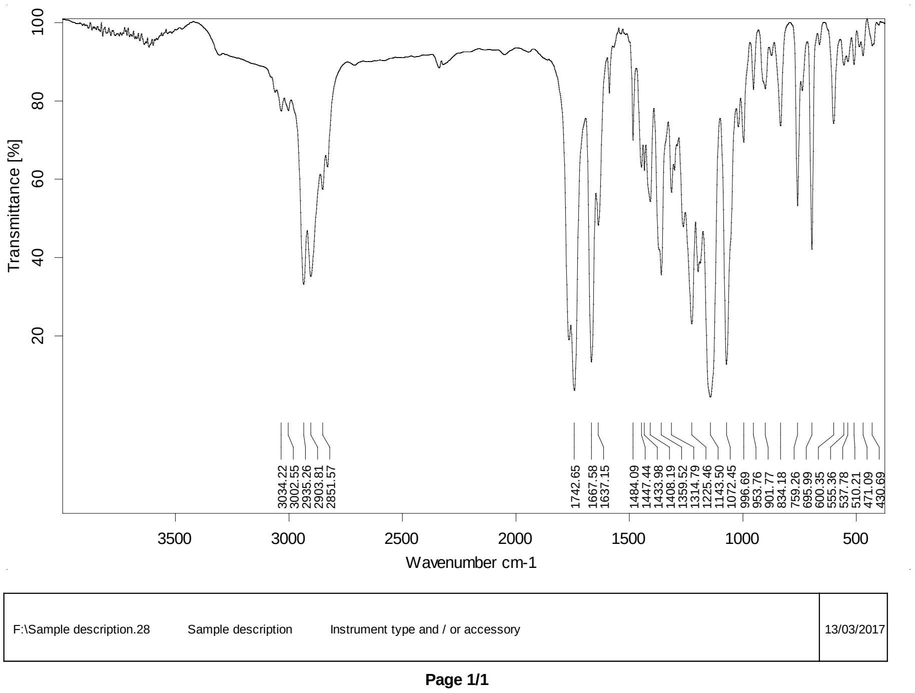

^{1}H NMR

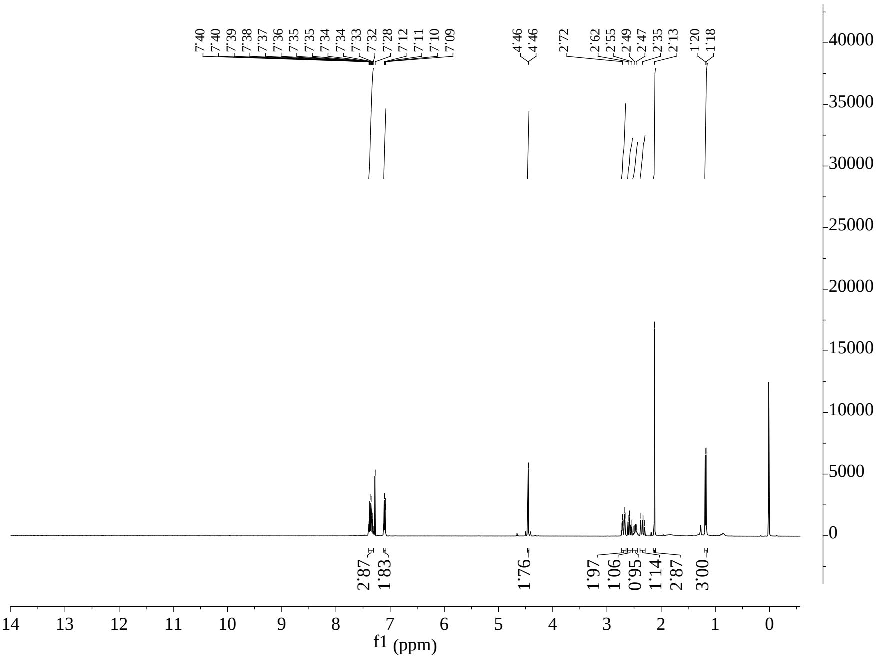

^{13}C NMR

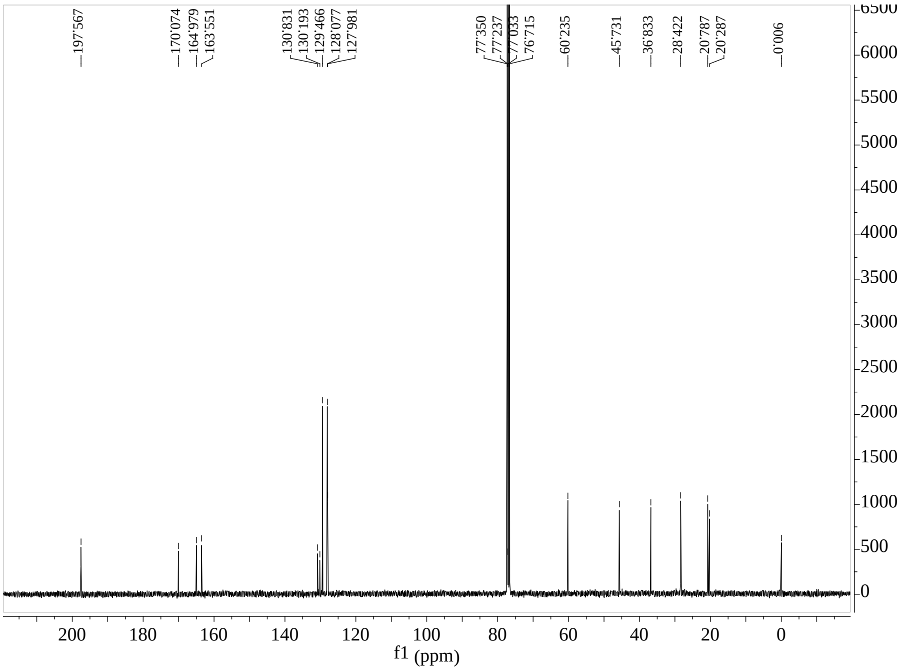
197.567
170.074
164.979
163.551
130.831
130.193
129.466
128.077
127.981
77.350
77.237
77.033
76.715
60.235
45.731
36.833
28.422
20.787
20.287
0.006
f1 (ppm)

HRMS

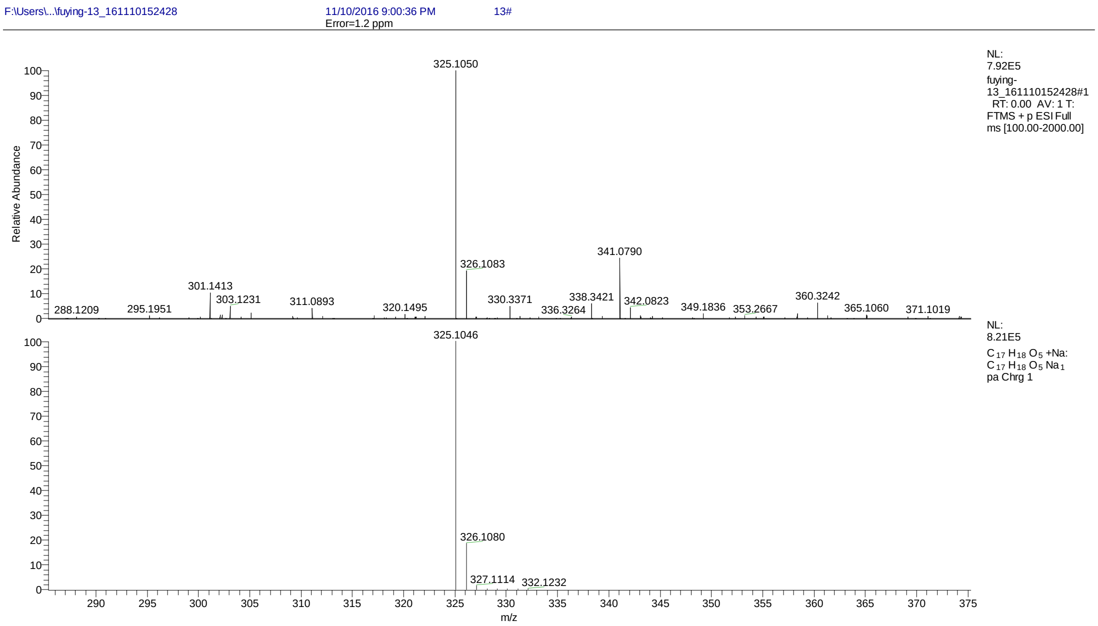
F:\Users\...\fuying-13_161110152428
11/10/2016 9:00:36 PM
13#
Error=1.2 ppm
NL: 7.92E5
fuying-13_161110152428#1 RT: 0.00 AV: 1 T: FTMS + p ESI Full ms [100.00-2000.00]
NL: 8.21E5
C 17 H 18 O 5 +Na:
C 17 H 18 O 5 Na 1
pa Chrg 1
Relative Abundance
m/z
325.1050
326.1083
341.0790
301.1413
303.1231
311.0893
320.1495
330.3371
338.3421
336.3264
342.0823
349.1836
353.2667
360.3242
365.1060
371.1019
288.1209
295.1951
325.1046
326.1080
327.1114
332.1232

3-(3-Acetylpropionoxy)-2-phenyl-5,5-dimethyl-2-cyclohexen-1-one (S16)

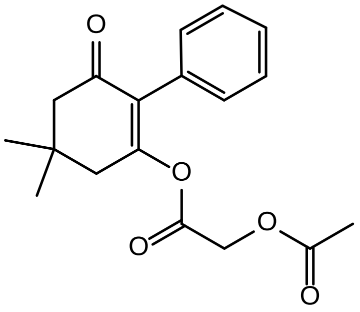

$C_{18}H_{20}O_5$

IR

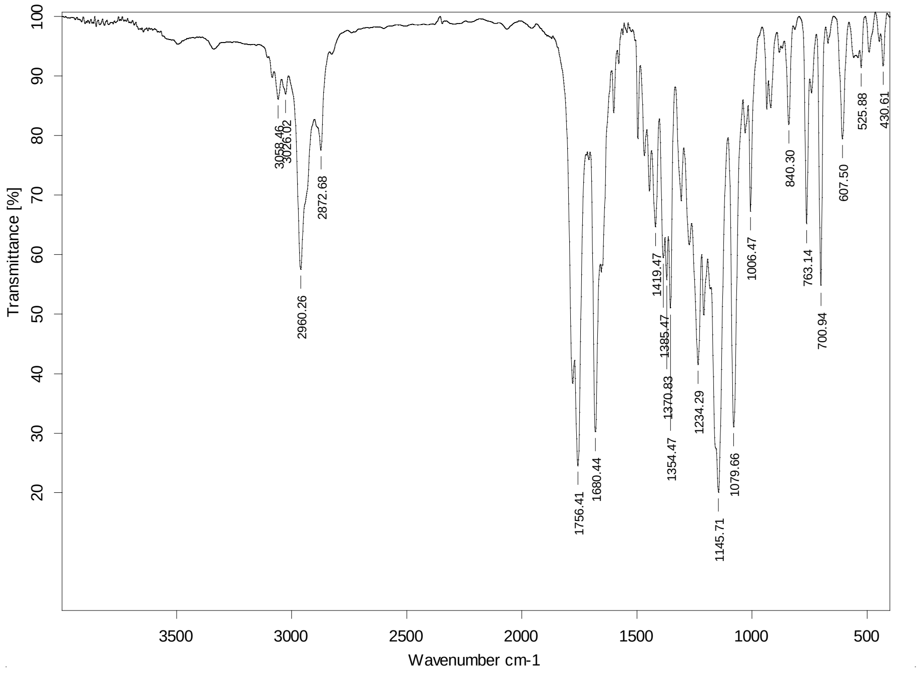

^{1}H NMR

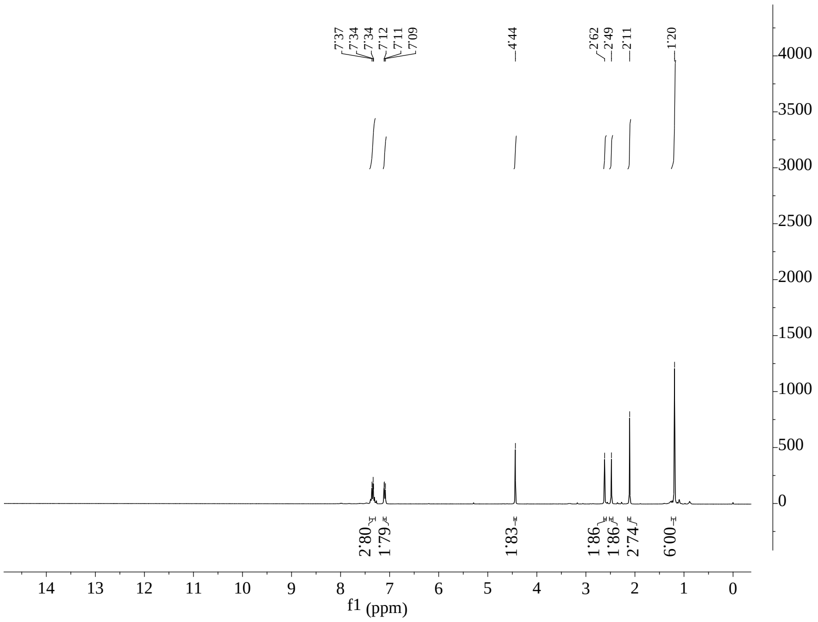

^{13}C NMR

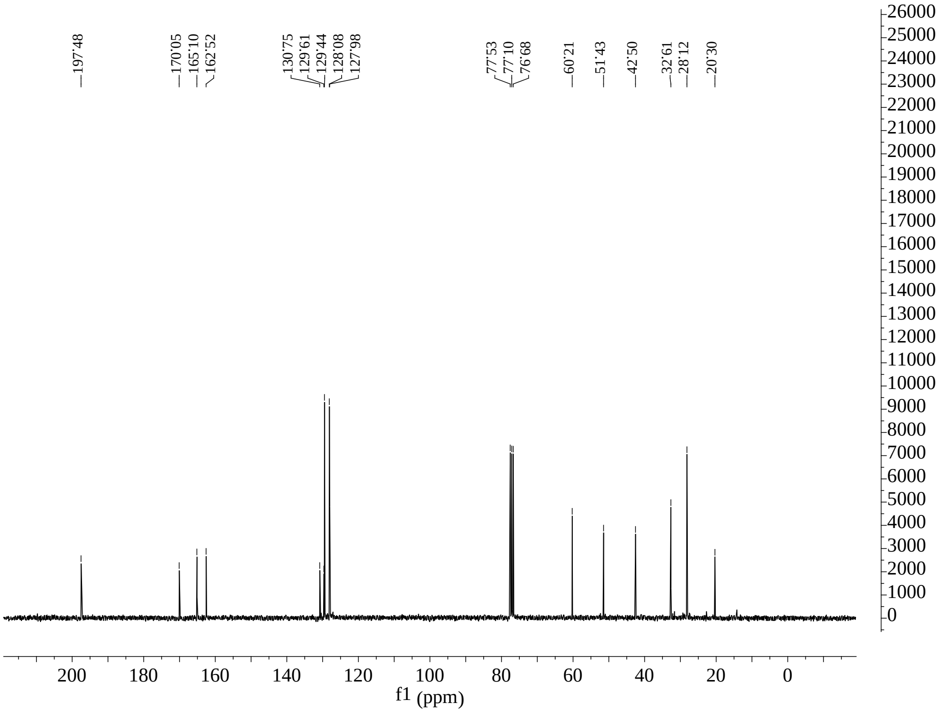

197.48
170.05
165.10
162.52
130.75
129.61
129.44
128.08
127.98
77.53
77.10
76.68
60.21
51.43
42.50
32.61
28.12
20.30
f1 (ppm)

HRMS

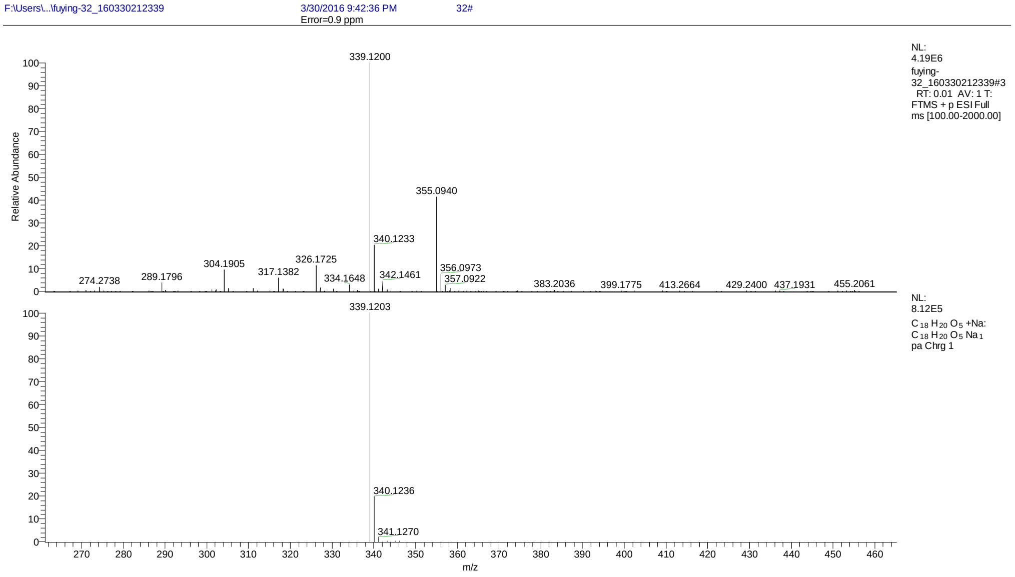

F:\Users\...\fuying-32_160330212339
3/30/2016 9:42:36 PM
Error=0.9 ppm
32#
Relative Abundance
339.1200
355.0940
340.1233
304.1905
326.1725
317.1382
334.1648
342.1461
356.0973
357.0922
274.2738
289.1796
383.2036
399.1775
413.2664
429.2400
437.1931
455.2061
NL:
4.19E6
fuying-
32_160330212339#3
RT: 0.01 AV: 1 T:
FTMS + p ESI Full
ms [100.00-2000.00]
339.1203
340.1236
341.1270
NL:
8.12E5
C 18 H 20 O 5 +Na:
C 18 H 20 O 5 Na 1
pa Chrg 1
m/z

3-[1-(2,4-Dichlorophenyl)-5-(trichloromethyl)-1H-1,2,4-triazol-3-yl]carbonyloxy-2-phenyl-2-cyclohexen-1-one (S17)

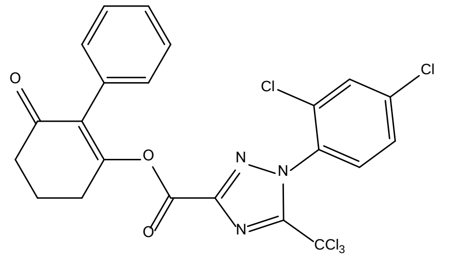

$C_{22}H_{14}Cl_5N_3O_3$

IR

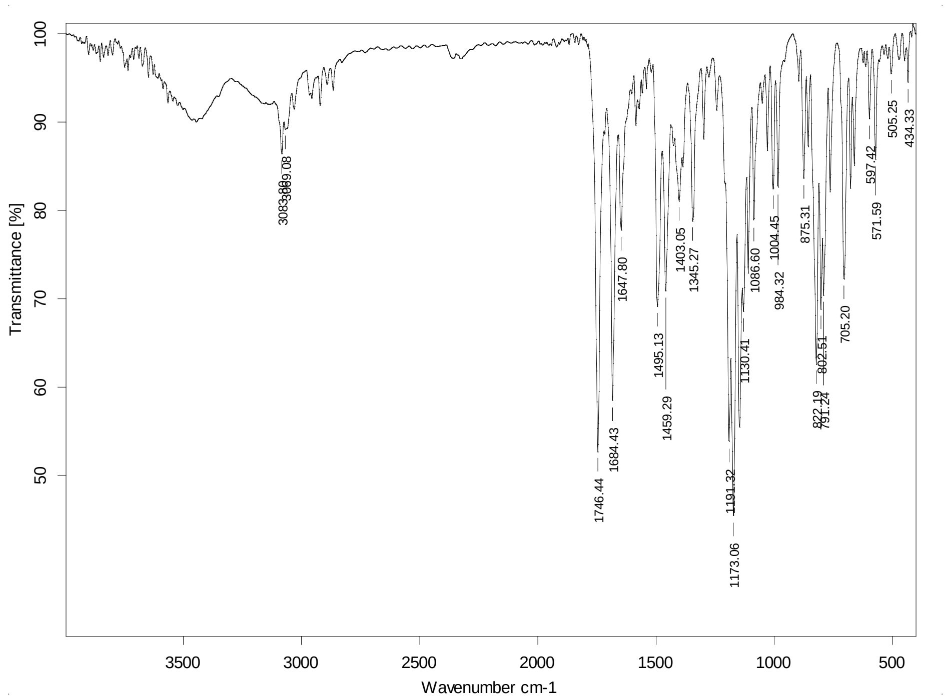

^{1}H NMR

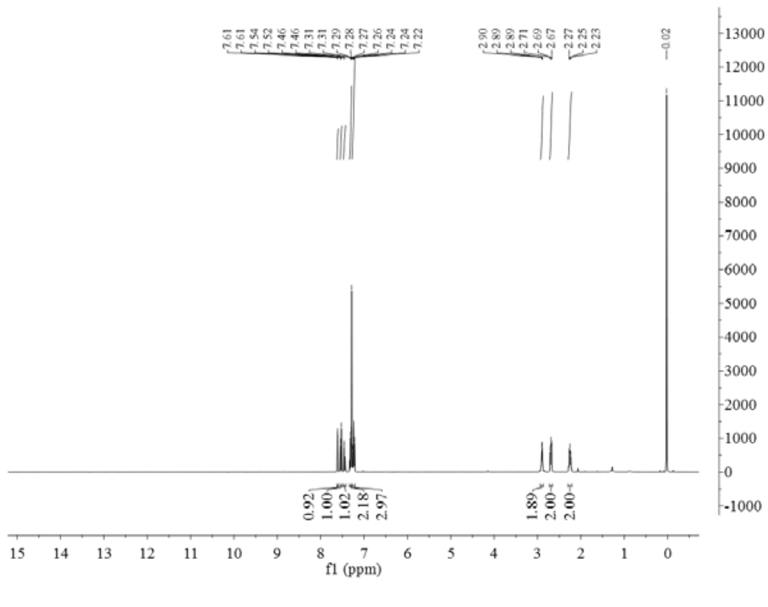

^{13}C NMR

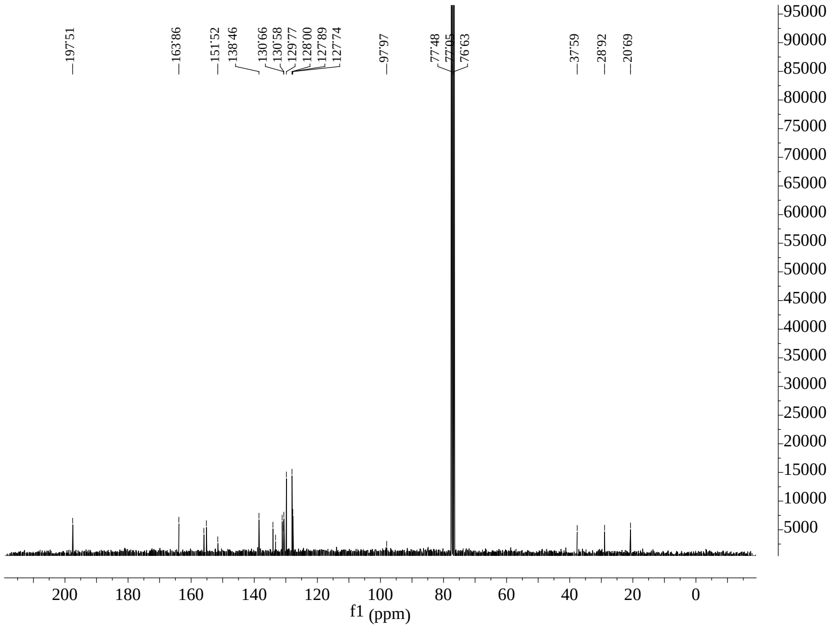
197.51
163.86
151.52
138.46
130.66
130.58
129.77
128.00
127.89
127.74
97.97
77.48
77.05
76.63
37.59
28.92
20.69
f1 (ppm)

HRMS

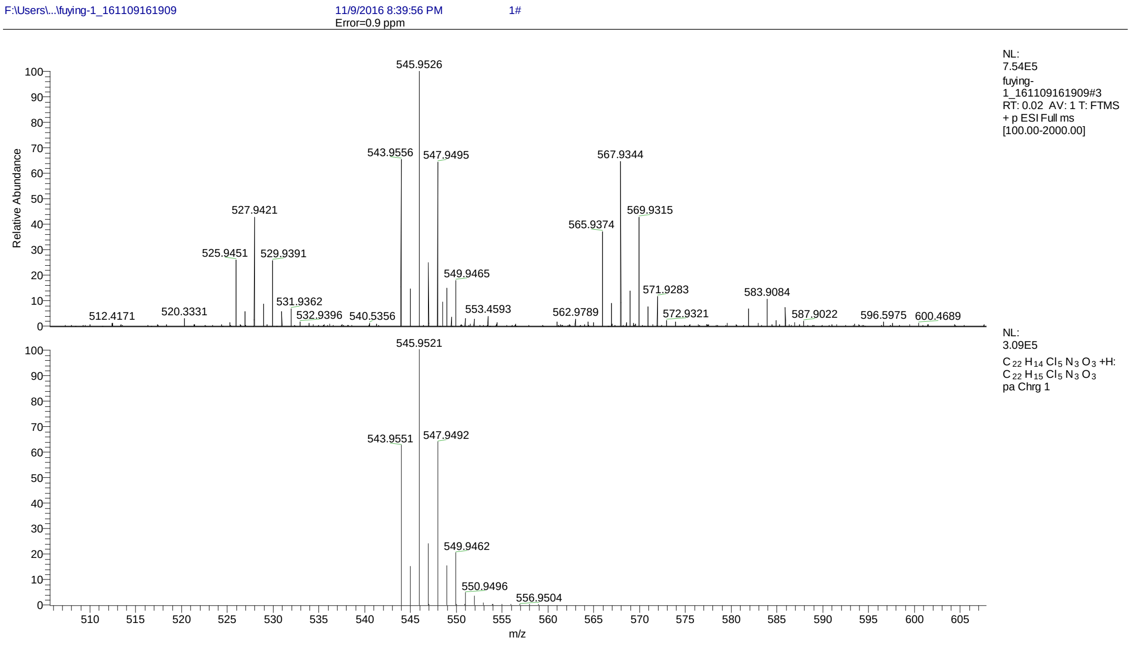
F:\Users\...\fuying-1_161109161909
11/9/2016 8:39:56 PM
1#
Error=0.9 ppm
NL: 7.54E5
fuying-1_161109161909#3
RT: 0.02 AV: 1 T: FTMS + p ESI Full ms [100.00-2000.00]
545.9526
543.9556
547.9495
567.9344
569.9315
565.9374
527.9421
525.9451
529.9391
549.9465
583.9084
571.9283
531.9362
553.4593
512.4171
520.3331
532.9396
540.5356
562.9789
572.9321
587.9022
596.5975
600.4689
NL: 3.09E5
C 22 H 14 Cl 5 N 3 O 3 +H:
C 22 H 15 Cl 5 N 3 O 3
pa Chrg 1
545.9521
543.9551
547.9492
549.9462
550.9496
556.9504
Relative Abundance
m/z

3-[1-(2,4-Dichlorophenyl)-5-(trichloromethyl)-1H-1,2,4-triazol-3-yl]carbonyloxy-2-phenyl-5-methyl-2-cyclohexen-1-one (S18)

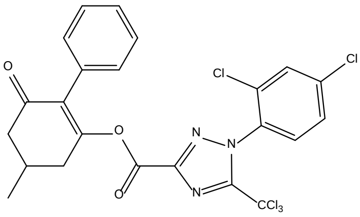

$C_{23}H_{16}Cl_5N_3O_3$

IR

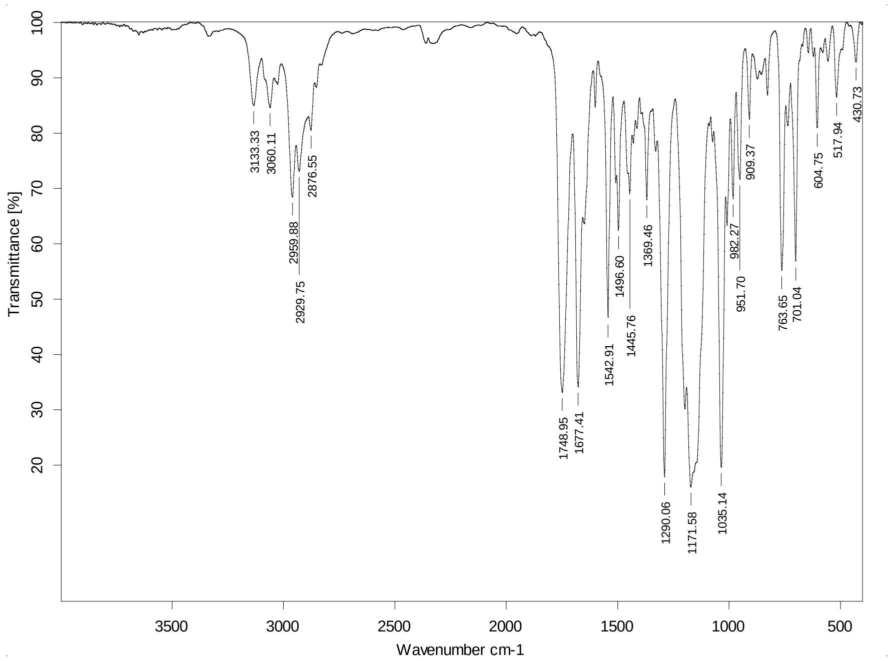

^{1}H NMR

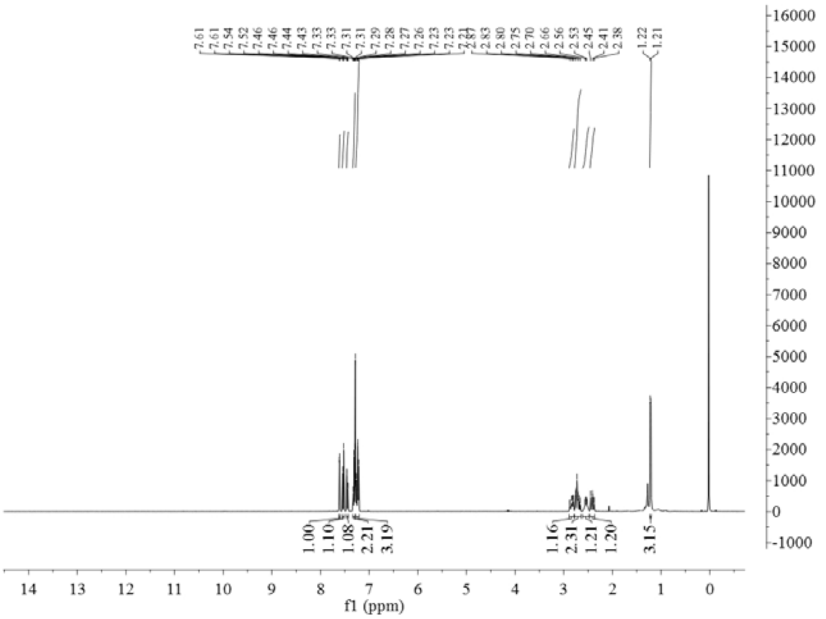

^{13}C NMR

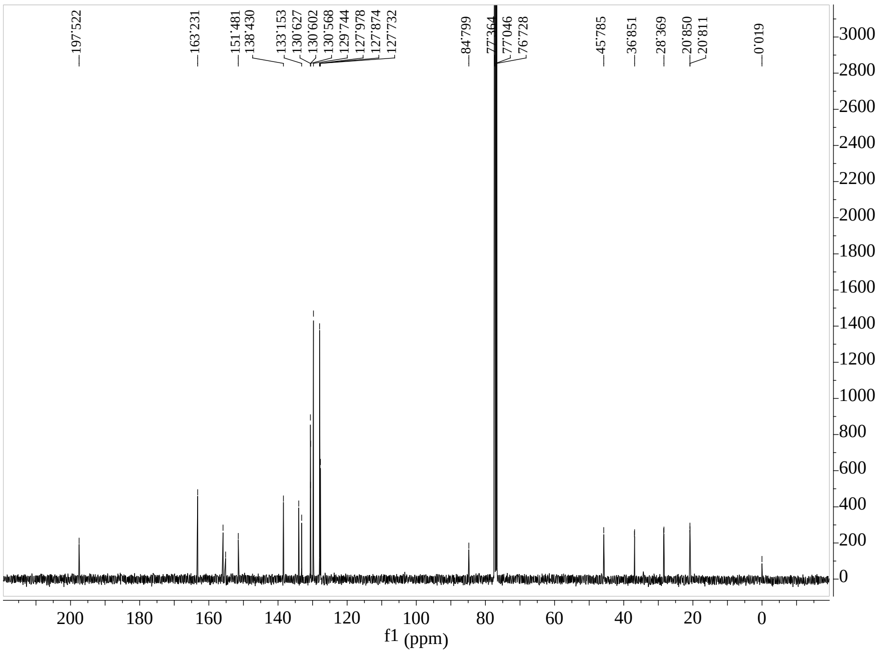
197.522
163.231
151.481
138.430
133.153
130.627
130.602
130.568
129.744
127.978
127.874
127.732
84.799
77.364
77.046
76.728
45.785
36.851
28.369
20.850
20.811
0.019
f1 (ppm)

HRMS

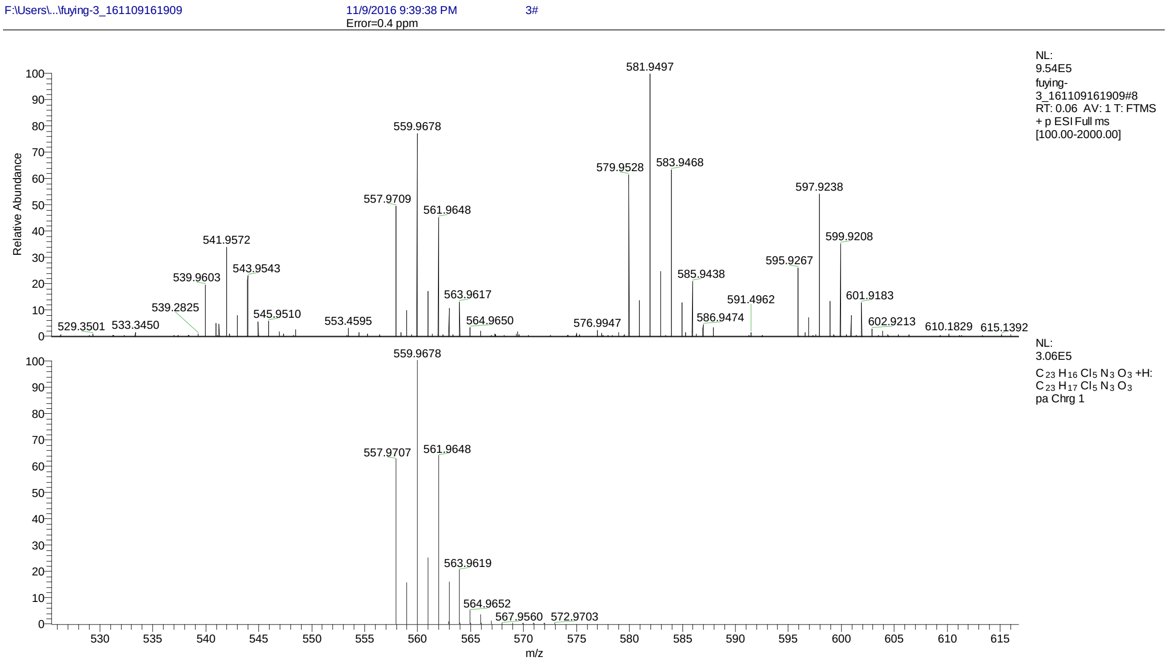
F:\Users\...\fuying-3_161109161909
11/9/2016 9:39:38 PM
3#
Error=0.4 ppm
NL: 9.54E5
fuying-3_161109161909#8
RT: 0.06 AV: 1 T: FTMS + p ESI Full ms [100.00-2000.00]
NL: 3.06E5
C 23 H 16 Cl 5 N 3 O 3 +H: C 23 H 17 Cl 5 N 3 O 3
pa Chrg 1
Relative Abundance
m/z
581.9497
559.9678
583.9468
579.9528
597.9238
557.9709
561.9648
541.9572
599.9208
543.9543
539.9603
595.9267
585.9438
563.9617
591.4962
601.9183
539.2825
545.9510
586.9474
553.4595
564.9650
576.9947
602.9213
529.3501
533.3450
610.1829
615.1392
557.9707
561.9648
563.9619
564.9652
567.9560
572.9703

3-[1-(2,4-Dichlorophenyl)-5-(trichloromethyl)-1H-1,2,4-triazol-3-yl]carbonyloxy-2-phenyl-5,5-dimethyl-2-cyclohexen-1-one (S19)

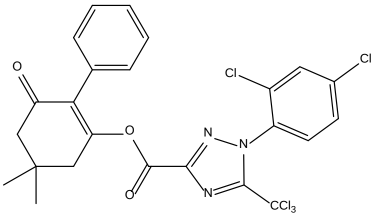

$C_{24}H_{18}N_3O_3Cl_5$

IR

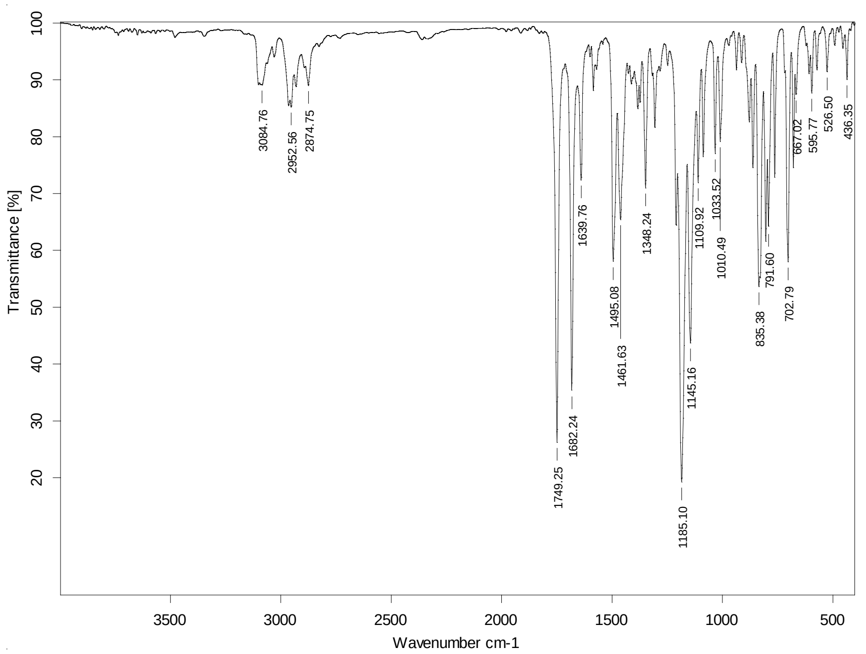

^{1}H NMR

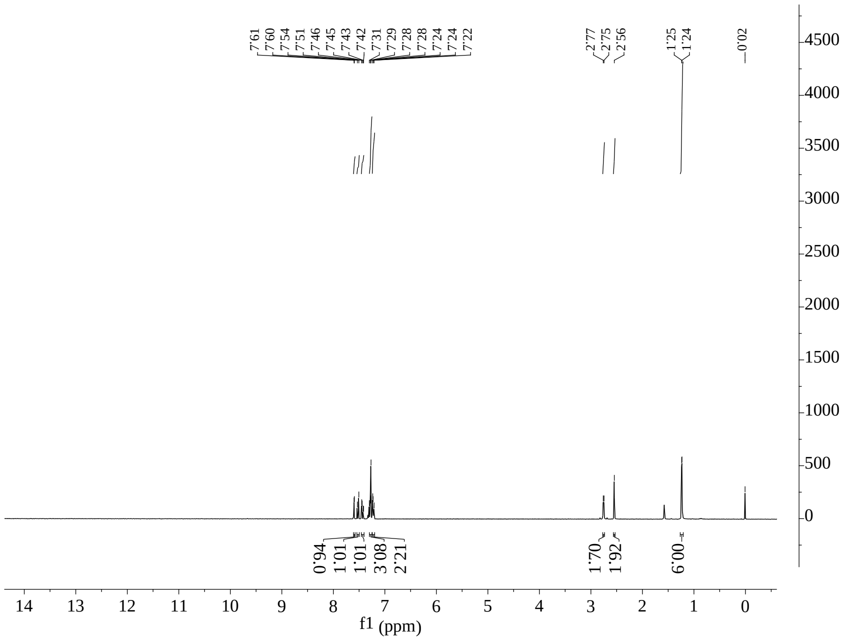

^{13}C NMR

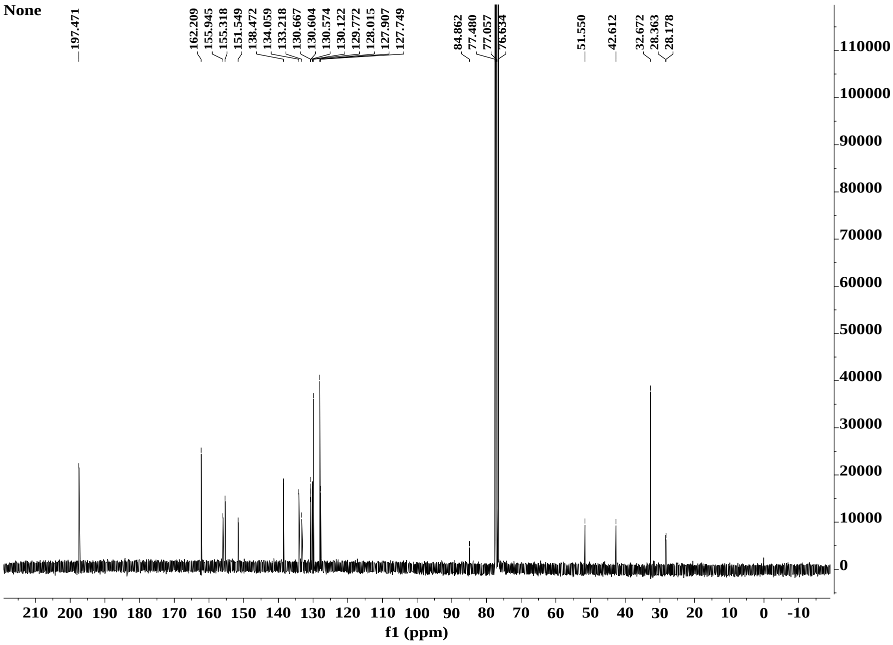
None
197.471
162.209
155.945
155.318
151.549
138.472
134.059
133.218
130.667
130.604
130.574
130.122
129.772
128.015
127.907
127.749
84.862
77.480
77.057
76.634
51.550
42.612
32.672
28.363
28.178
f1 (ppm)

HRMS

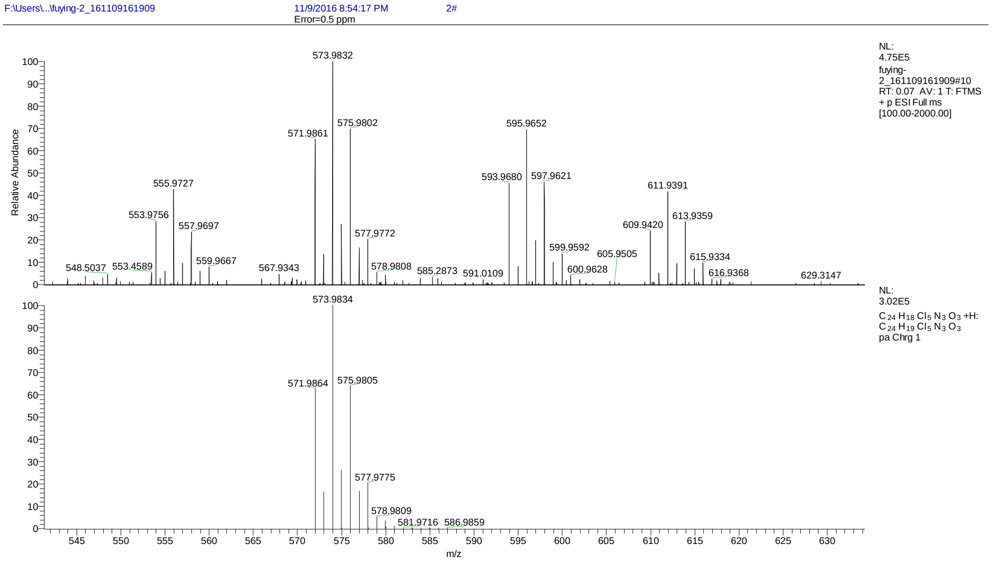
F:\Users\...\fuying-2_161109161909
11/9/2016 8:54:17 PM
2#
Error=0.5 ppm
NL:
4.75E5
fuying-2_161109161909#10
RT: 0.07 AV: 1 T: FTMS + p ESI Full ms [100.00-2000.00]
Relative Abundance
573.9832
575.9802
571.9861
595.9652
593.9680
597.9621
555.9727
611.9391
553.9756
613.9359
557.9697
609.9420
577.9772
599.9592
605.9505
615.9334
548.5037
553.4589
559.9667
567.9343
578.9808
585.2873
591.0109
600.9628
616.9368
629.3147
NL:
3.02E5
C 24 H18 Cl5 N3 O3 +H:
C 24 H19 Cl5 N3 O3
pa Chrg 1
573.9834
571.9864
575.9805
577.9775
578.9809
581.9716
586.9859
m/z

3-[5-Methyl-3-phenylisoxazole-4-carbonyloxy]-2-phenyl-2-cyclohexen-1-one (S20)

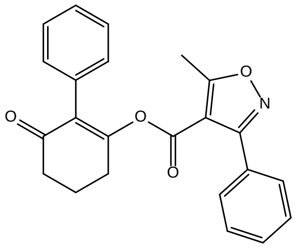

$C_{23}H_{19}NO_4$

IR

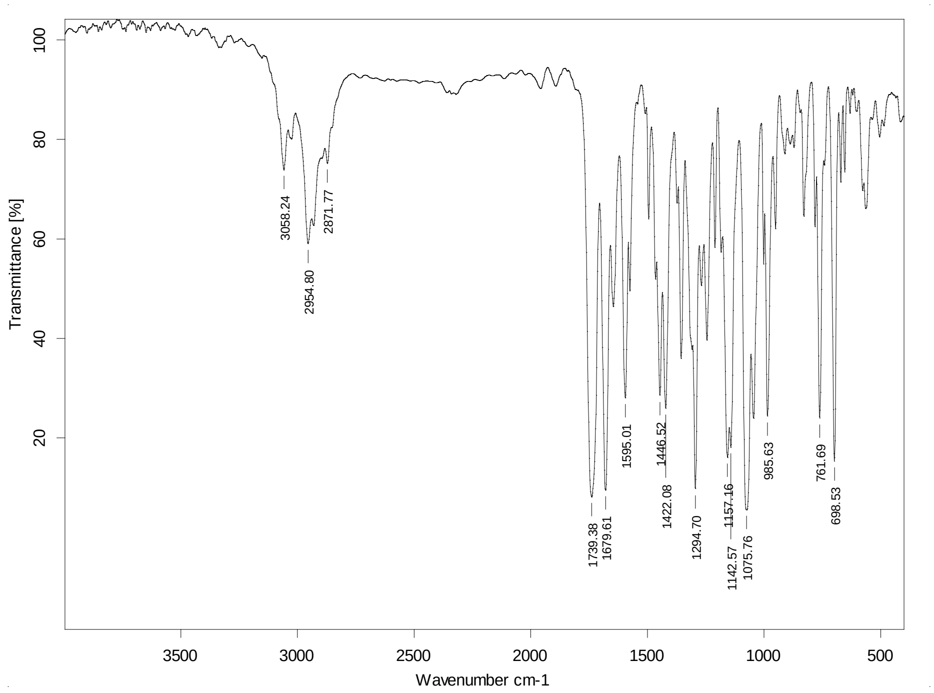

^1H NMR

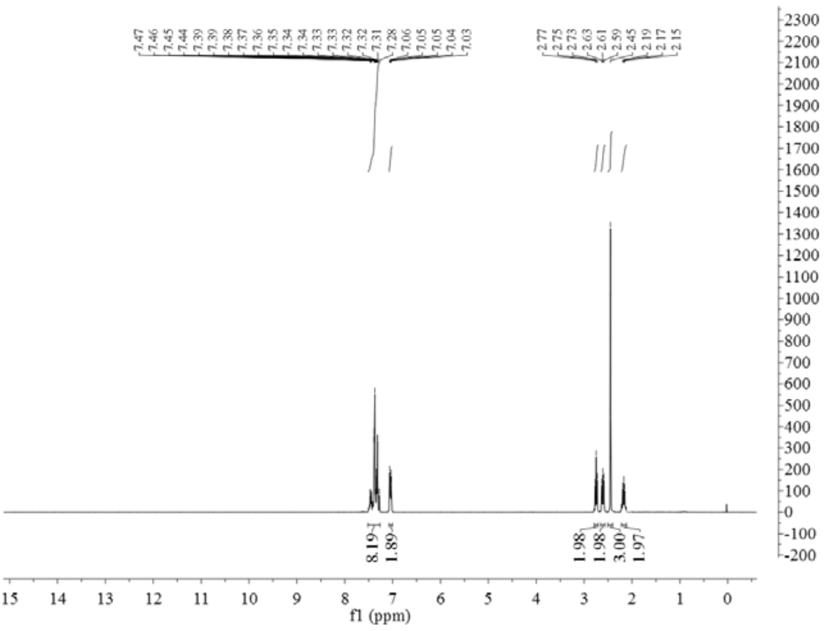

^{13}C NMR

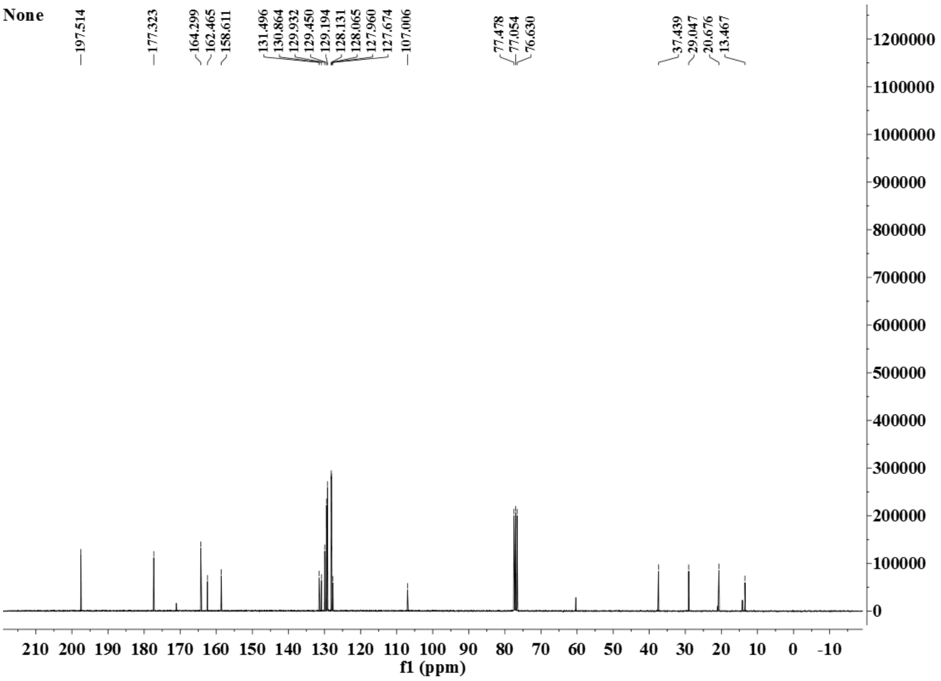

None
197.514
177.323
164.299
162.465
158.611
131.496
130.864
129.932
129.450
129.194
128.131
128.065
127.960
127.674
107.006
77.478
77.054
76.630
37.439
29.047
20.676
13.467
f1 (ppm)

HRMS

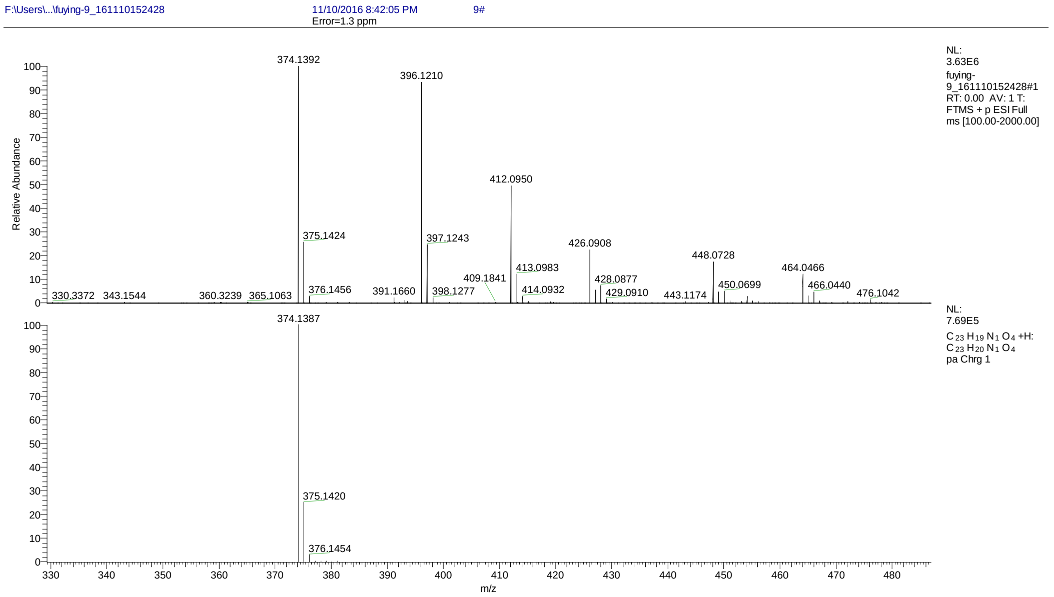

F:\Users\...\fuying-9_161110152428
11/10/2016 8:42:05 PM
9#
Error=1.3 ppm
NL: 3.63E6
fuying-9_161110152428#1 RT: 0.00 AV: 1 T: FTMS + p ESI Full ms [100.00-2000.00]
374.1392
396.1210
412.0950
375.1424
397.1243
426.0908
448.0728
464.0466
NL: 7.69E5
C 23 H 19 N 1 O 4 +H: C 23 H 20 N 1 O 4 pa Chrg 1
374.1387
375.1420
376.1454
Relative Abundance
m/z

3-[5-Methyl-3-phenylisoxazole-4-carbonyloxy]-2-phenyl-5-methyl-2-cyclohexen-1-on (S21)

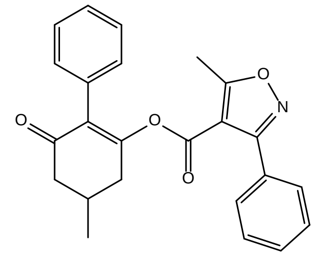

$C_{24}H_{21}NO_4$

IR

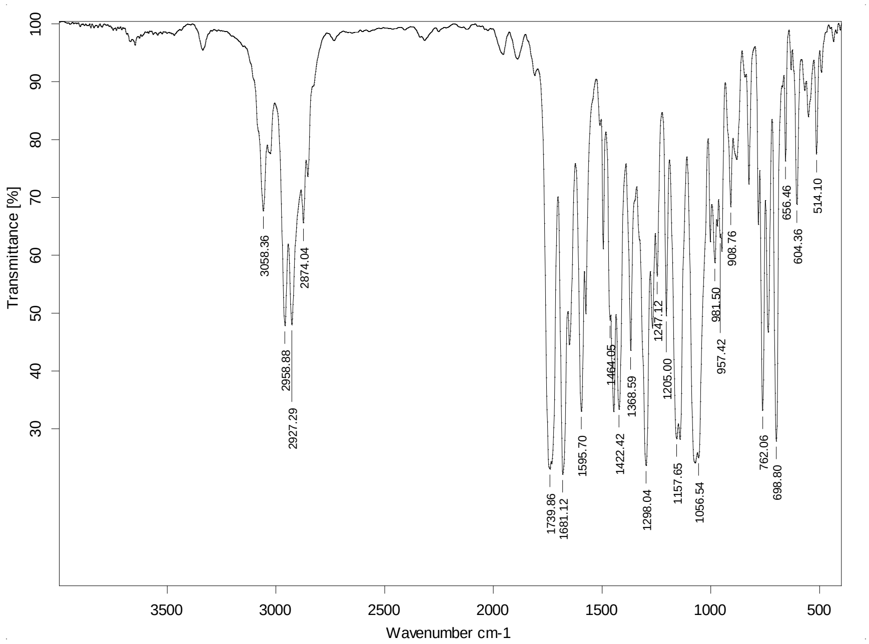

^{1}H NMR

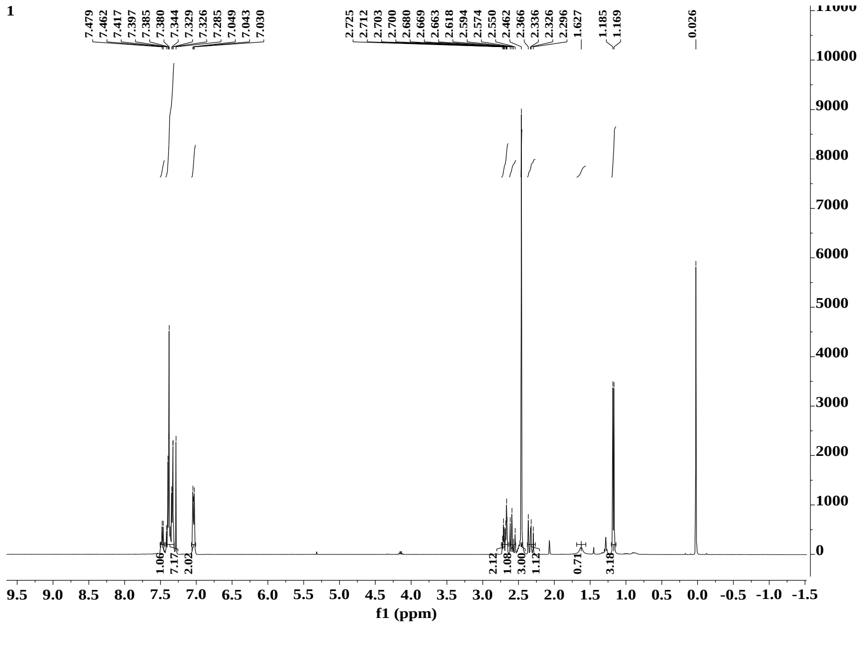

^{13}C NMR

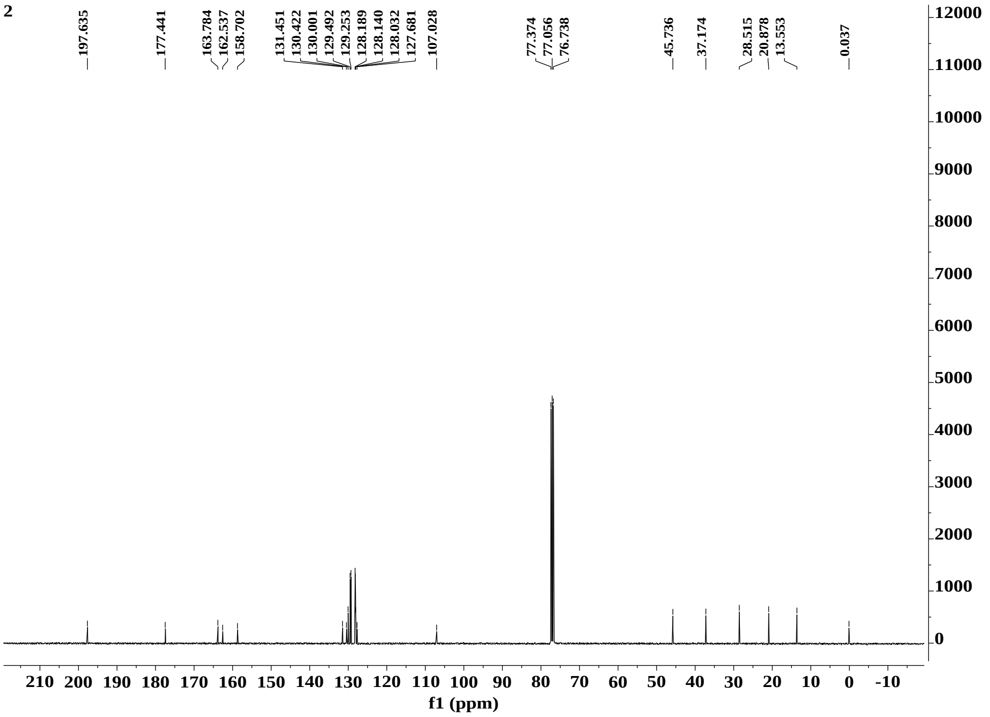
2
197.635
177.441
163.784
162.537
158.702
131.451
130.422
130.001
129.492
129.253
128.189
128.140
128.032
127.681
107.028
77.374
77.056
76.738
45.736
37.174
28.515
20.878
13.553
0.037
f1 (ppm)

HRMS

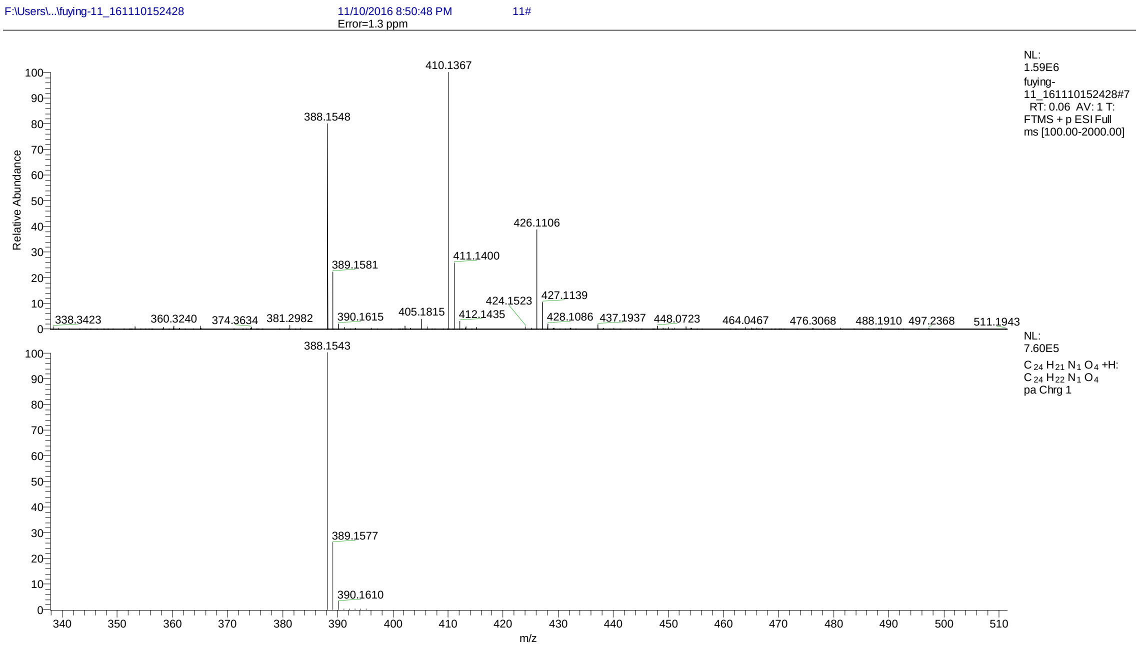
F:\Users\...\fuying-11_161110152428
11/10/2016 8:50:48 PM
Error=1.3 ppm
11#
NL: 1.59E6
fuying-11_161110152428#7 RT: 0.06 AV: 1 T: FTMS + p ESI Full ms [100.00-2000.00]
410.1367
388.1548
426.1106
411.1400
389.1581
424.1523
427.1139
NL: 7.60E5
C 24 H 21 N 1 O 4 +H: C 24 H 22 N 1 O 4 pa Chrg 1
388.1543
389.1577
390.1610
Relative Abundance
m/z

3-[5-Methyl-3-phenylisoxazole-4-carbonyloxy]-2-phenyl-5,5-dimethyl-2-cyclohexen-1-one (S22)

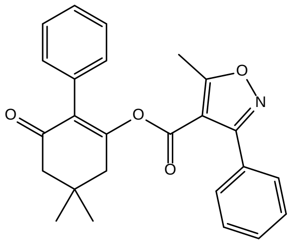

$C_{25}H_{23}NO_4$

IR

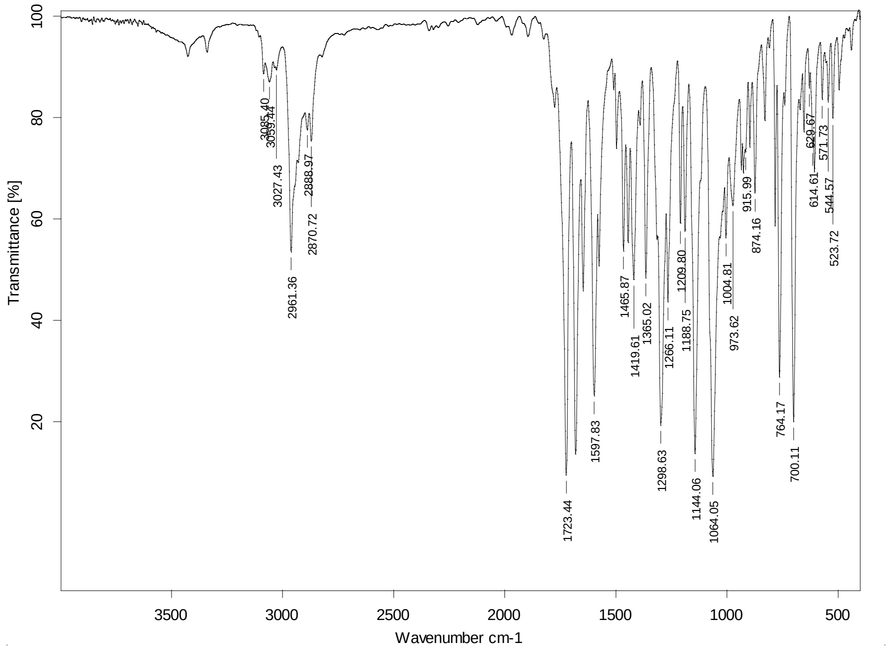

^{1}H NMR

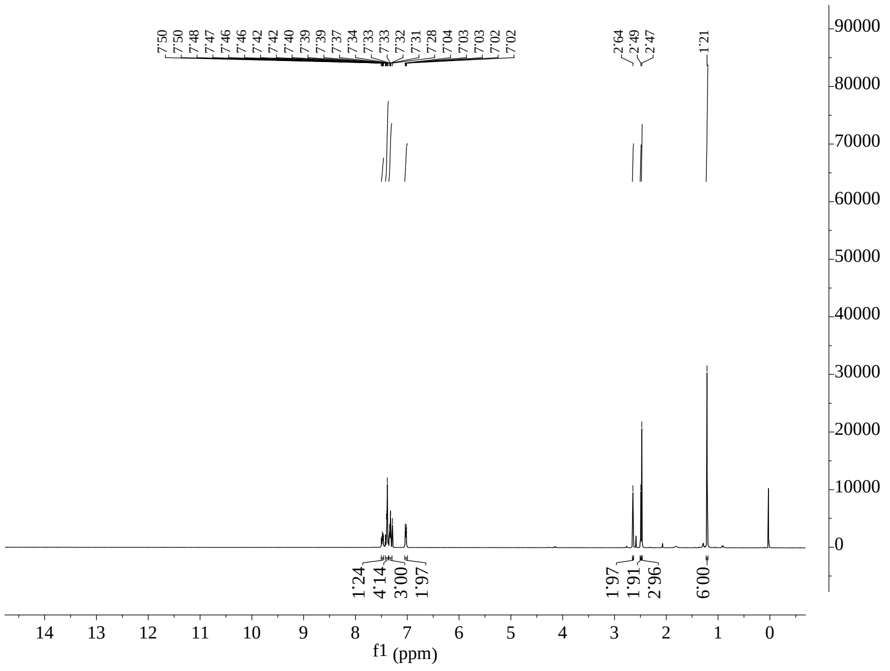

^{13}C NMR

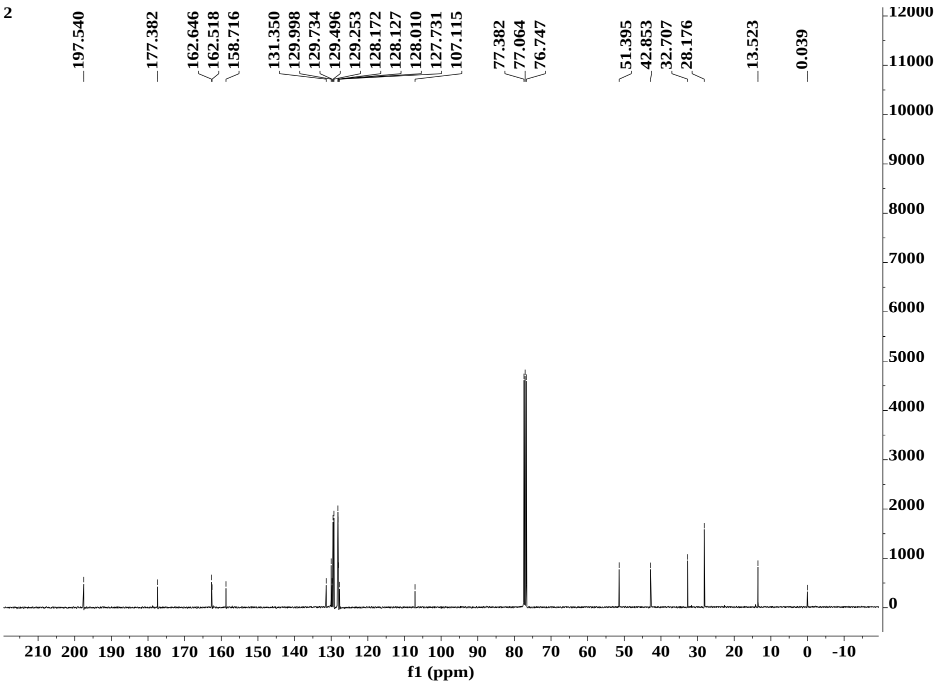
2
197.540
177.382
162.646
162.518
158.716
131.350
129.998
129.734
129.496
129.253
128.172
128.127
128.010
127.731
107.115
77.382
77.064
76.747
51.395
42.853
32.707
28.176
13.523
0.039
f1 (ppm)

HRMS

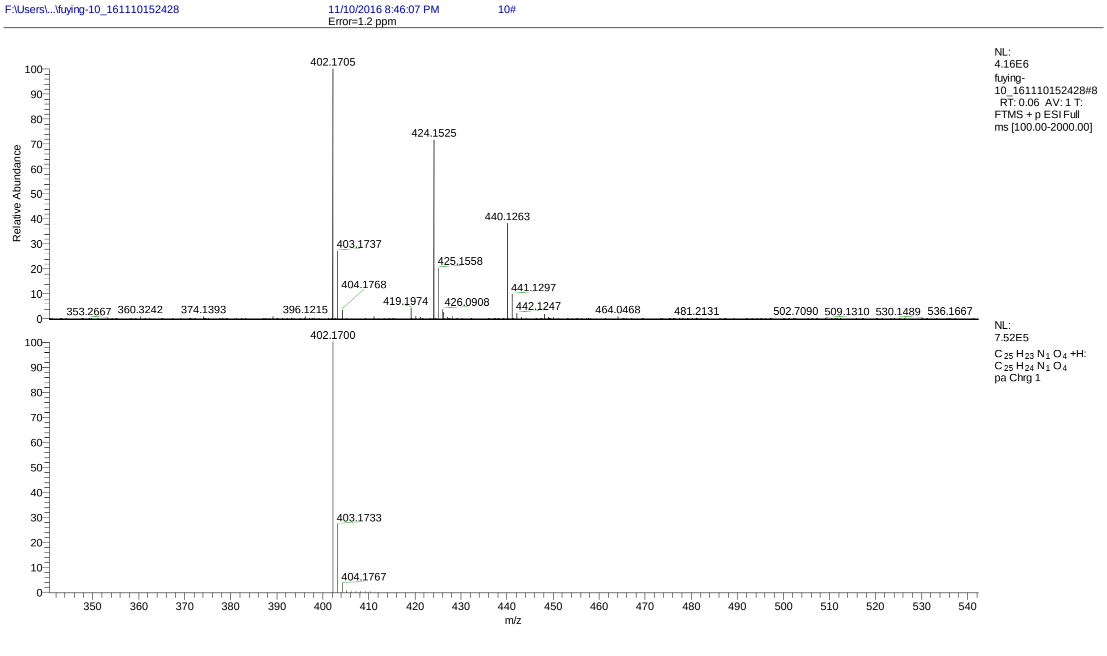
F:\Users\...\fuying-10_161110152428
11/10/2016 8:46:07 PM
Error=1.2 ppm
10#
NL:
4.16E6
fuying-10_161110152428#8 RT: 0.06 AV: 1 T: FTMS + p ESI Full ms [100.00-2000.00]
402.1705
424.1525
440.1263
403.1737
425.1558
404.1768
441.1297
419.1974
426.0908
442.1247
353.2667
360.3242
374.1393
396.1215
464.0468
481.2131
502.7090
509.1310
530.1489
536.1667
NL:
7.52E5
C25 H23 N1 O4 +H:
C25 H24 N1 O4
pa Chrg 1
402.1700
403.1733
404.1767
Relative Abundance
m/z

3-[5-Methyl-3-(2-fluoro-6-chlorophenyl)isoxazole-4-carbonyloxy]-2-phenyl-2-cyclohexen-1-one (S23)

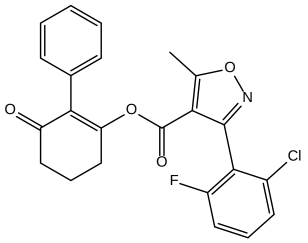

$C_{23}H_{17}ClFNO_4$

IR

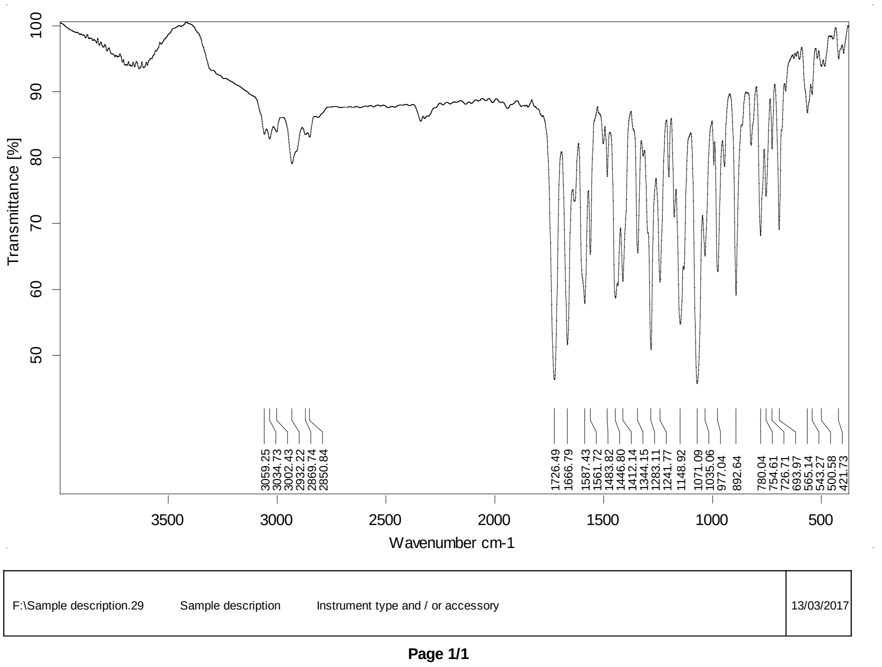

^{1}H NMR

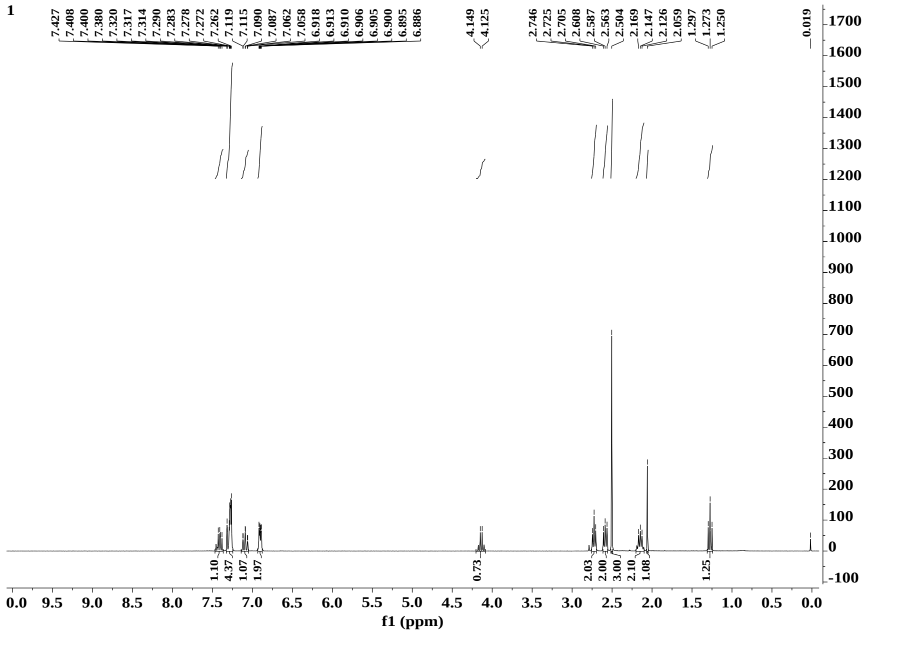

^{13}C NMR

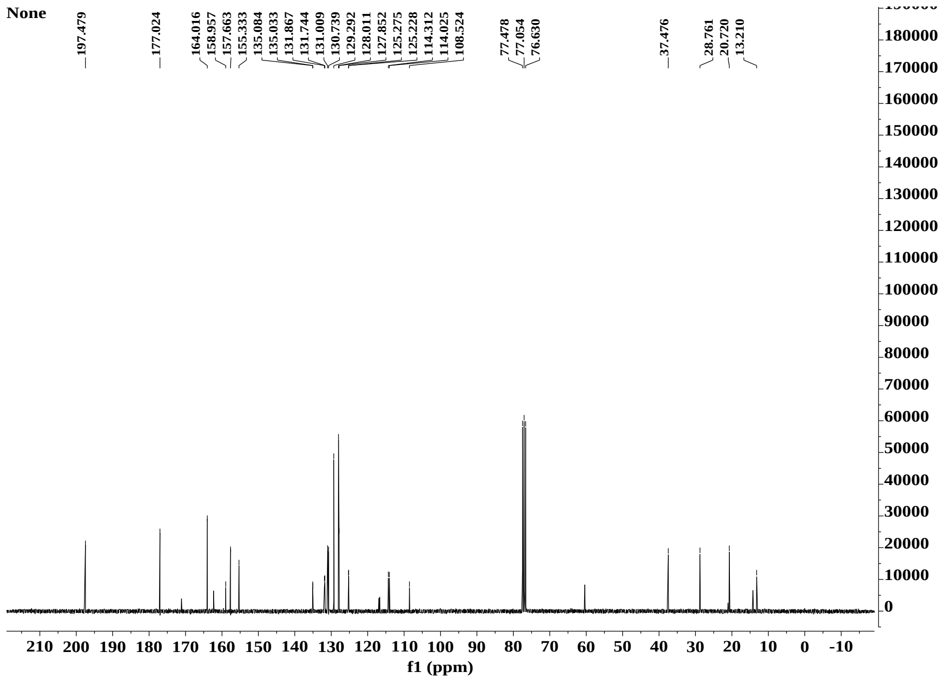
None
197.479
177.024
164.016
158.957
157.663
155.333
135.084
135.033
131.867
131.744
131.009
130.739
129.292
128.011
127.852
125.275
125.228
114.312
114.025
108.524
77.478
77.054
76.630
37.476
28.761
20.720
13.210
f1 (ppm)

HRMS

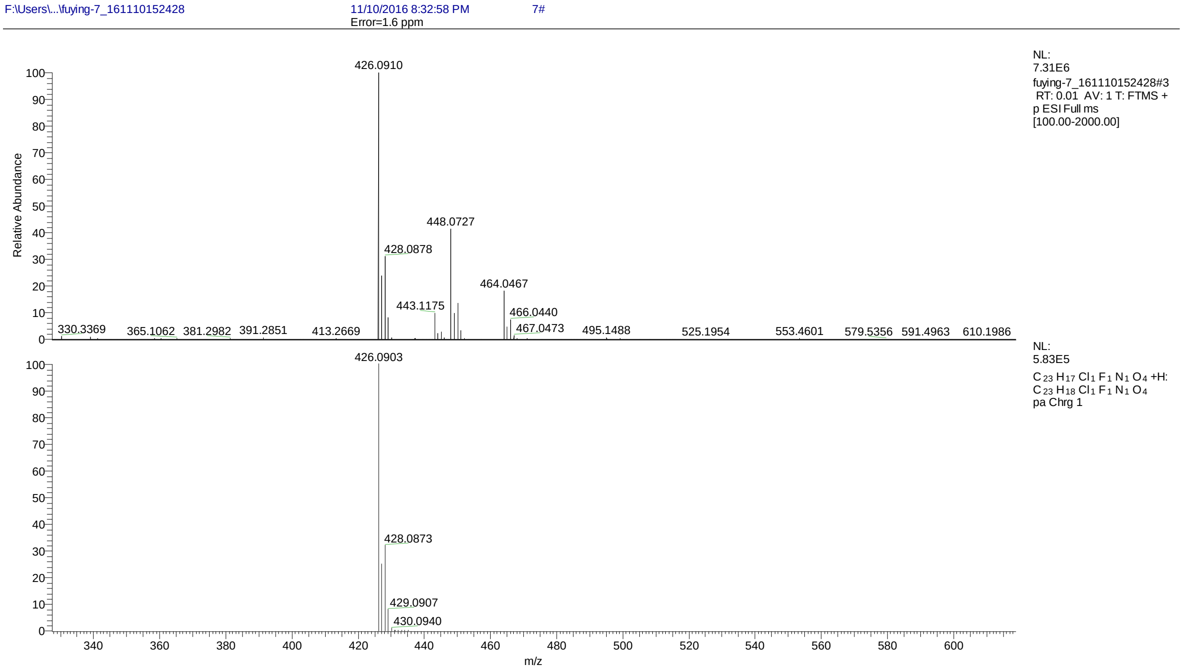
F:\Users\...\fuying-7_161110152428
11/10/2016 8:32:58 PM
7#
Error=1.6 ppm
NL: 7.31E6
fuying-7_161110152428#3 RT: 0.01 AV: 1 T: FTMS + p ESI Full ms [100.00-2000.00]
426.0910
448.0727
428.0878
464.0467
443.1175
466.0440
467.0473
330.3369
365.1062
381.2982
391.2851
413.2669
495.1488
525.1954
553.4601
579.5356
591.4963
610.1986
NL: 5.83E5
C 23 H 17 Cl 1 F 1 N 1 O 4 +H: C 23 H 18 Cl 1 F 1 N 1 O 4 pa Chrg 1
426.0903
428.0873
429.0907
430.0940
Relative Abundance
m/z

3-[5-Methyl-3-(2-fluoro-6-chlorophenyl)isoxazole-4-carbonyloxy]-2-phenyl-5-methyl-2-cyclohexen-1-one (S24)

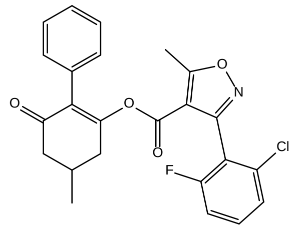

$C_{24}H_{19}ClFNO_4$

IR

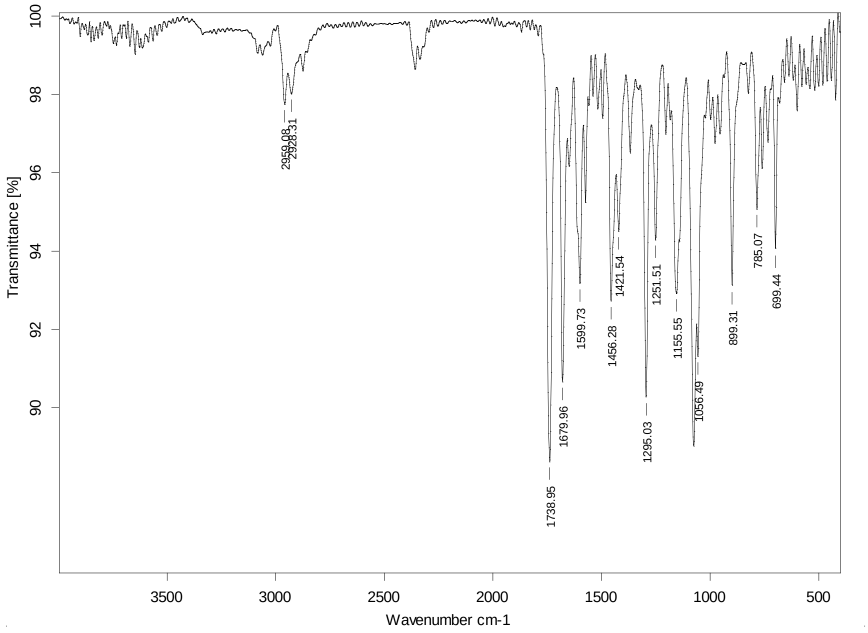

^{1}H NMR

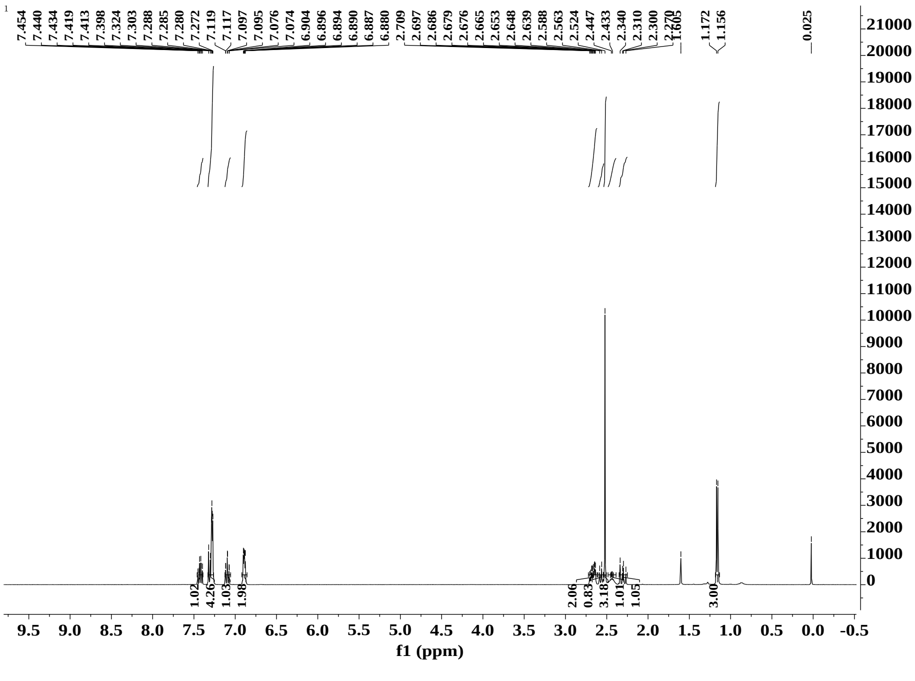

^{13}C NMR

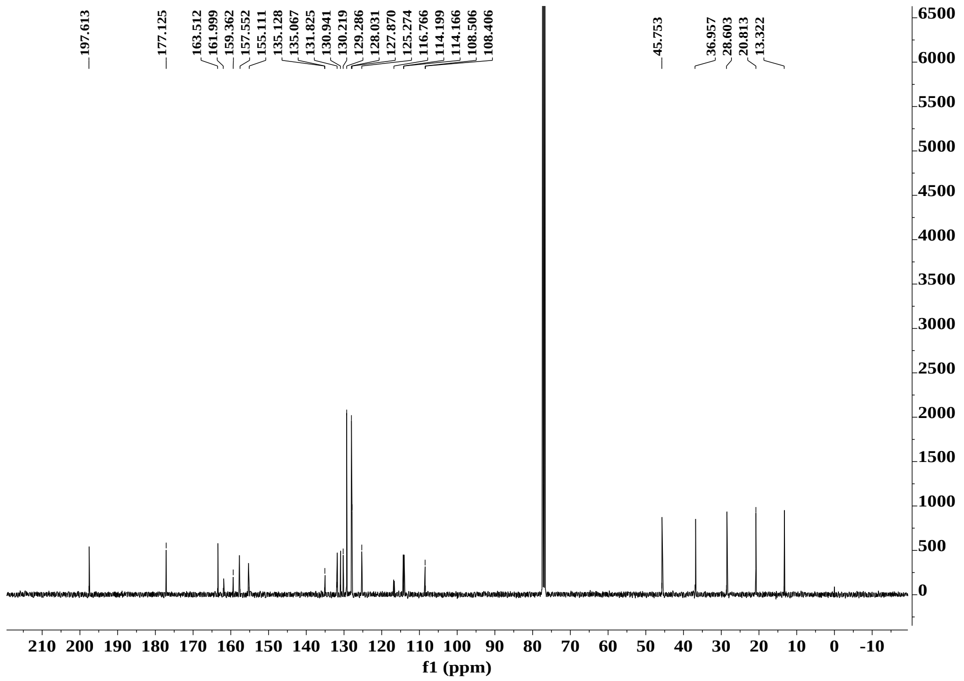

197.613
177.125
163.512
161.999
159.362
157.552
155.111
135.128
135.067
131.825
130.941
130.219
129.286
128.031
127.870
125.274
116.766
114.199
114.166
108.506
108.406
45.753
36.957
28.603
20.813
13.322
f1 (ppm)

HRMS

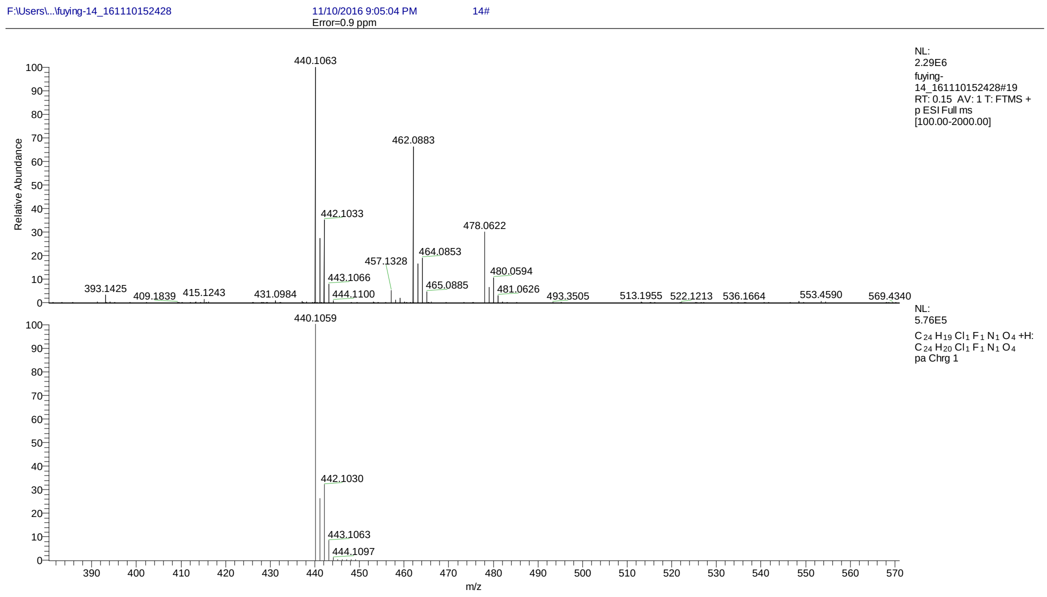

F:\Users\...\fuying-14_161110152428
11/10/2016 9:05:04 PM
14#
Error=0.9 ppm
NL: 2.29E6 fuying-14_161110152428#19 RT: 0.15 AV: 1 T: FTMS + p ESI Full ms [100.00-2000.00]
440.1063
462.0883
442.1033
478.0622
457.1328
464.0853
443.1066
480.0594
444.1100
465.0885
481.0626
393.1425
409.1839
415.1243
431.0984
493.3505
513.1955
522.1213
536.1664
553.4590
569.4340
NL: 5.76E5
C 24 H 19 Cl 1 F 1 N 1 O 4 +H: C 24 H 20 Cl 1 F 1 N 1 O 4 pa Chrg 1
440.1059
442.1030
443.1063
444.1097
Relative Abundance
m/z

3-[5-Methyl-3-(2-fluoro-6-chlorophenyl)isoxazole-4-carbonyloxy]-2-phenyl-5,5-dimethyl-2-cyclohexen-1-one (S25)

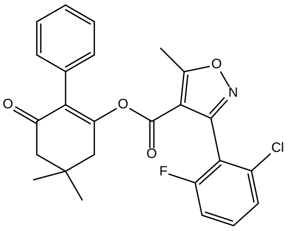

$C_{25}H_{21}ClFNO_4$

IR

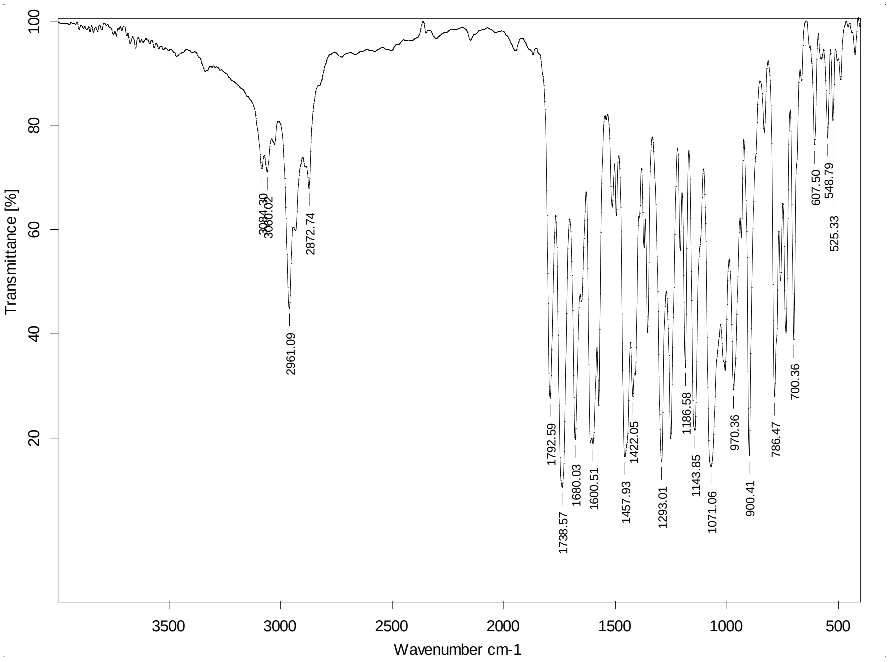

^{1}H NMR

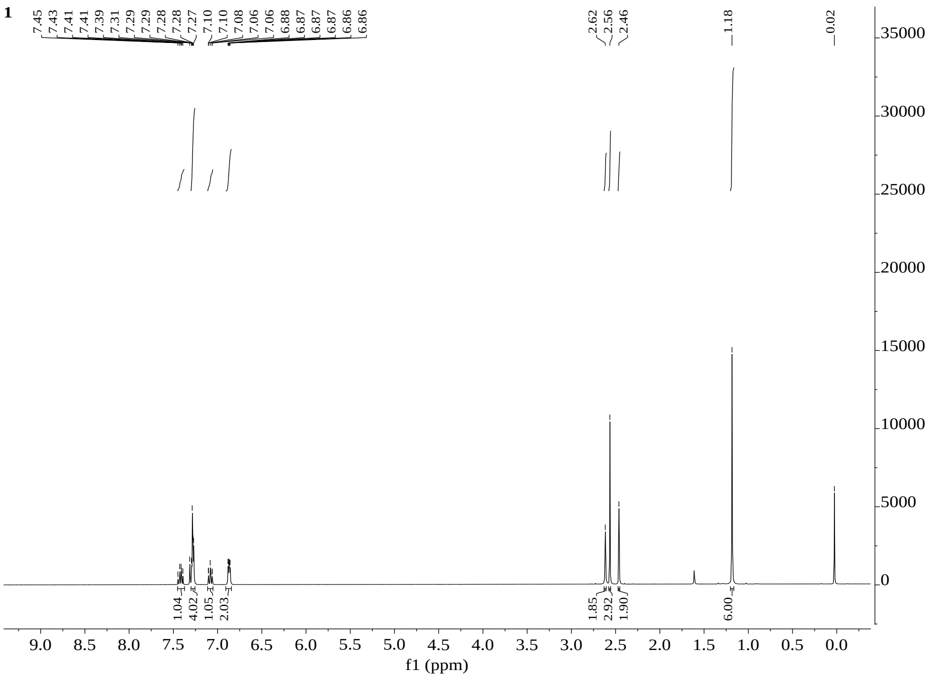

^{13}C NMR

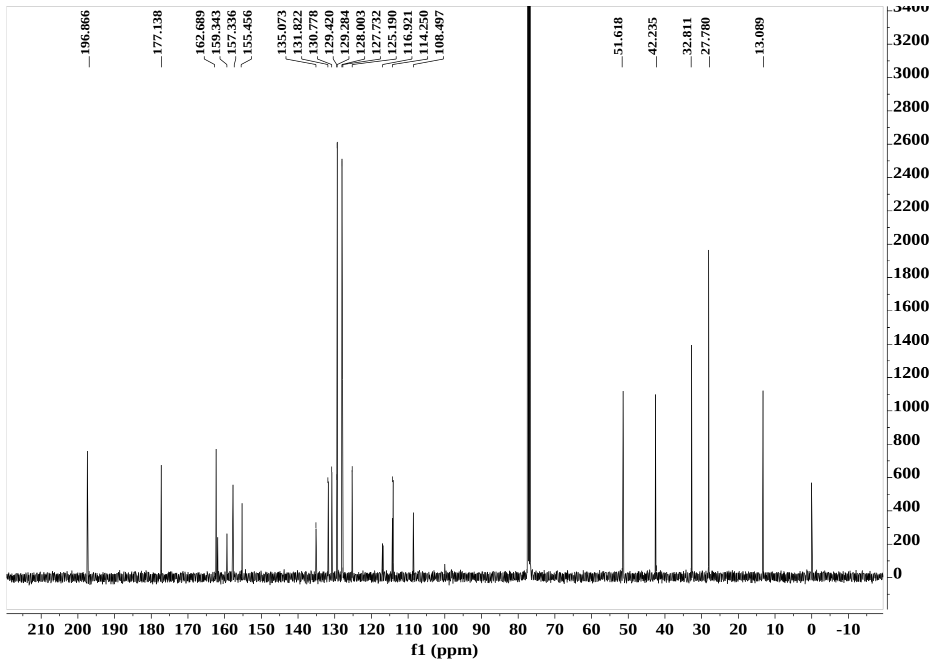
196.866
177.138
162.689
159.343
157.336
155.456
135.073
131.822
130.778
129.420
129.284
128.003
127.732
125.190
116.921
114.250
108.497
51.618
42.235
32.811
27.780
13.089
f1 (ppm)

HRMS

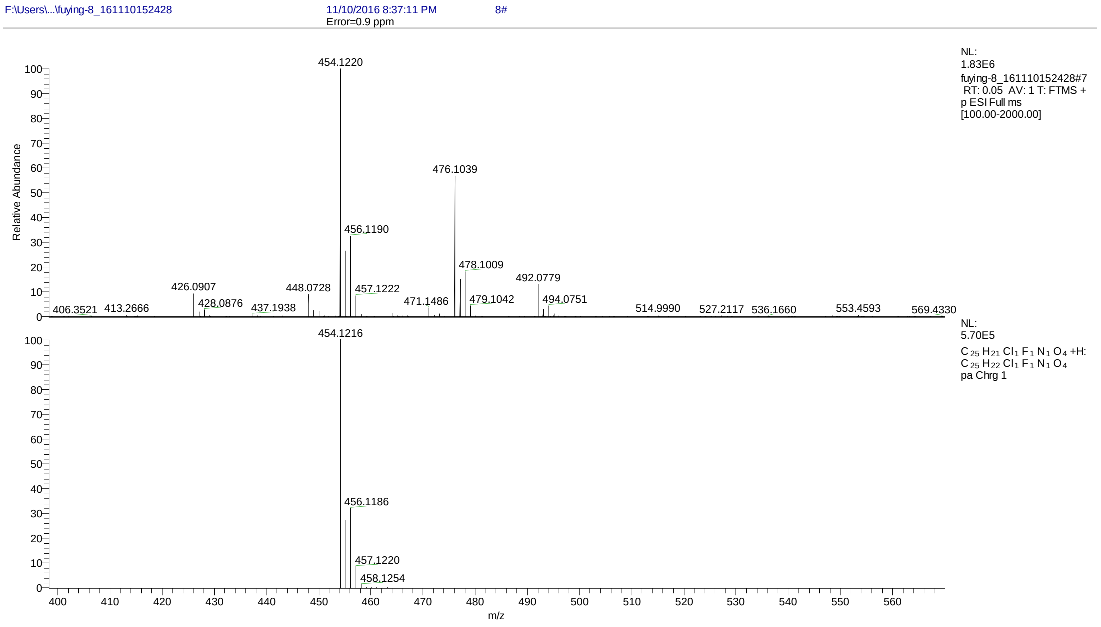
F:\Users\...\fuying-8_161110152428
11/10/2016 8:37:11 PM
8#
Error=0.9 ppm
NL: 1.83E6
fuying-8_161110152428#7 RT: 0.05 AV: 1 T: FTMS + p ESI Full ms [100.00-2000.00]
Relative Abundance
454.1220
476.1039
456.1190
478.1009
492.0779
426.0907
448.0728
457.1222
471.1486
479.1042
494.0751
428.0876
437.1938
406.3521
413.2666
514.9990
527.2117
536.1660
553.4593
569.4330
NL: 5.70E5
C 25 H 21 Cl 1 F 1 N 1 O 4 +H: C 25 H 22 Cl 1 F 1 N 1 O 4 pa Chrg 1
454.1216
456.1186
457.1220
458.1254
m/z

3-[(2-Trifluoromethyl-4-methyl)pyrazoloyloxy]-2-phenyl-2-cyclohexen-1-one (S26)

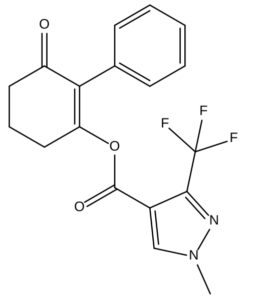

$C_{18}H_{15}F_3N_2O_3$

IR

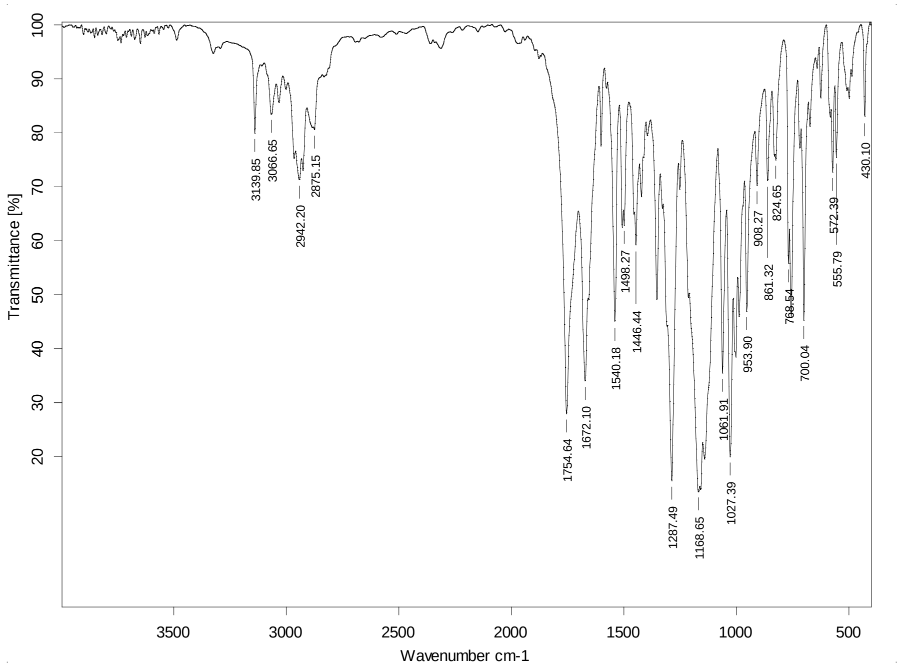

^{1}H NMR

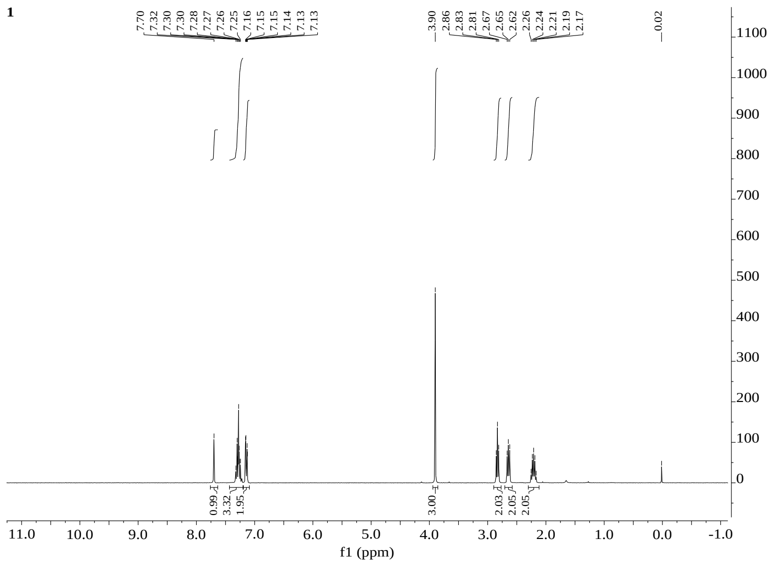

^{13}C NMR

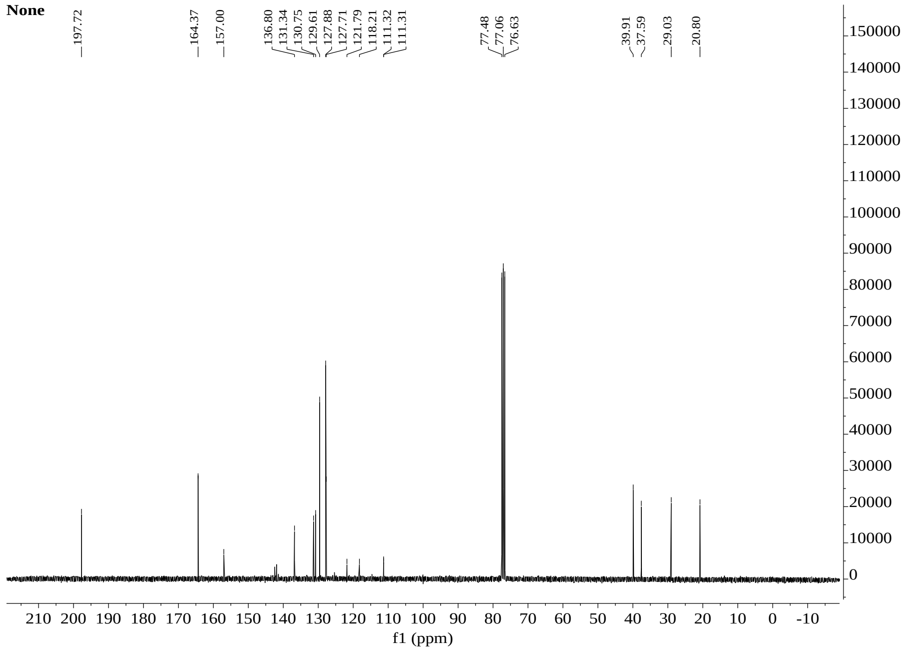
None
197.72
164.37
157.00
136.80
131.34
130.75
129.61
127.88
127.71
121.79
118.21
111.32
111.31
77.48
77.06
76.63
39.91
37.59
29.03
20.80
f1 (ppm)

HRMS

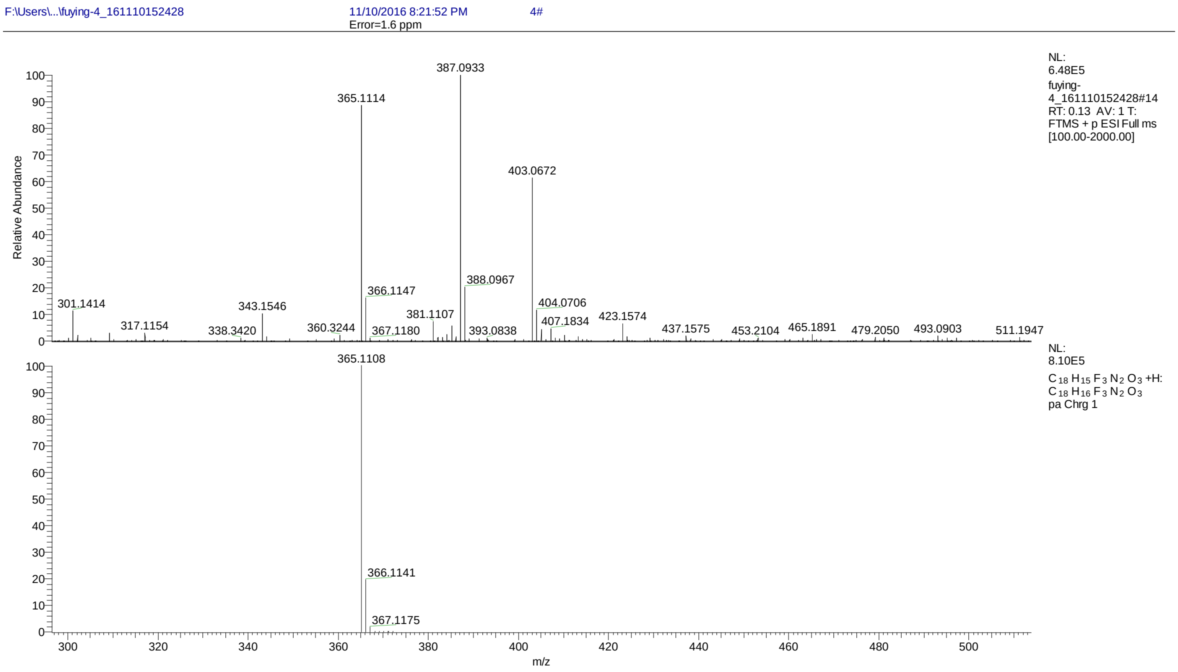
F:\Users\...\fuying-4_161110152428
11/10/2016 8:21:52 PM
4#
Error=1.6 ppm
NL: 6.48E5
fuying-4_161110152428#14 RT: 0.13 AV: 1 T: FTMS + p ESI Full ms [100.00-2000.00]
387.0933
365.1114
403.0672
388.0967
366.1147
404.0706
301.1414
343.1546
381.1107
407.1834
423.1574
317.1154
338.3420
360.3244
367.1180
393.0838
437.1575
453.2104
465.1891
479.2050
493.0903
511.1947
NL: 8.10E5
C18 H15 F3 N2 O3 +H: C18 H16 F3 N2 O3 pa Chrg 1
365.1108
366.1141
367.1175
Relative Abundance
m/z

3-[(2-Trifluoromethyl-4-methyl)pyrazoloyloxy]-2-phenyl-5-methyl-2-cyclohexen-1-one (S27)

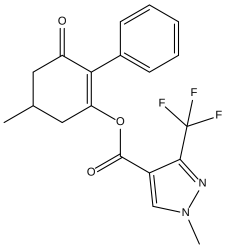

$C_{19}H_{17}F_3N_2O_3$

IR

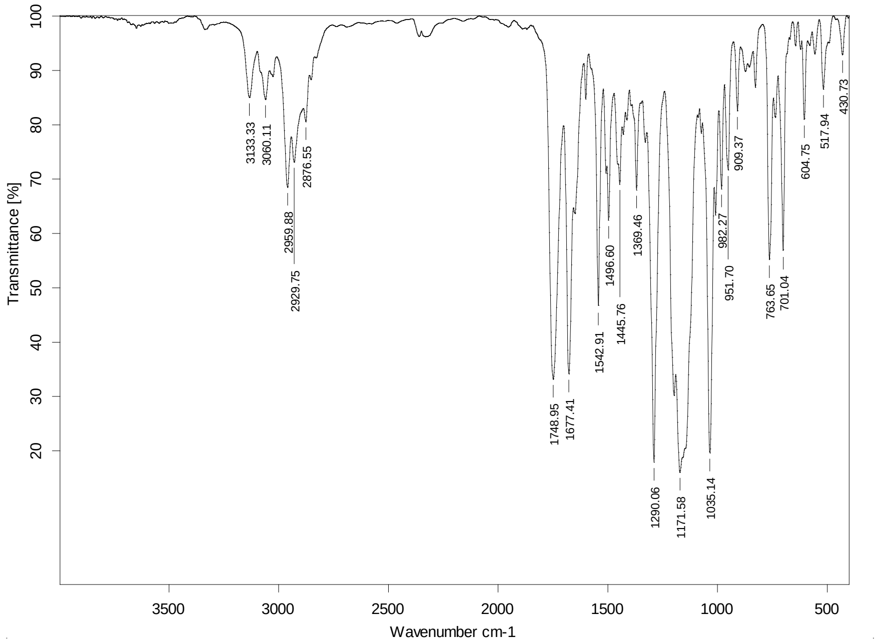

^{1}H NMR

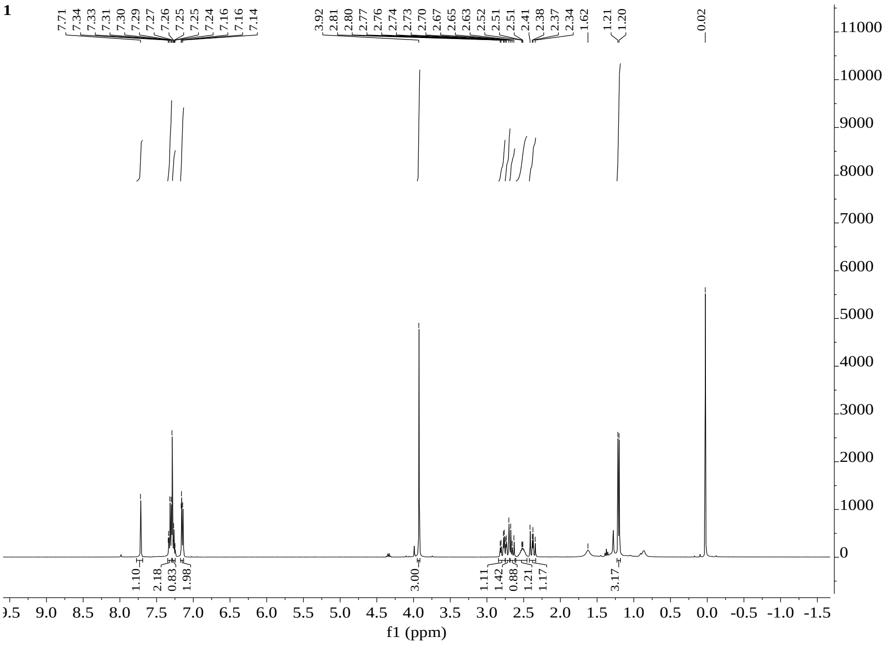

^{13}C NMR

2

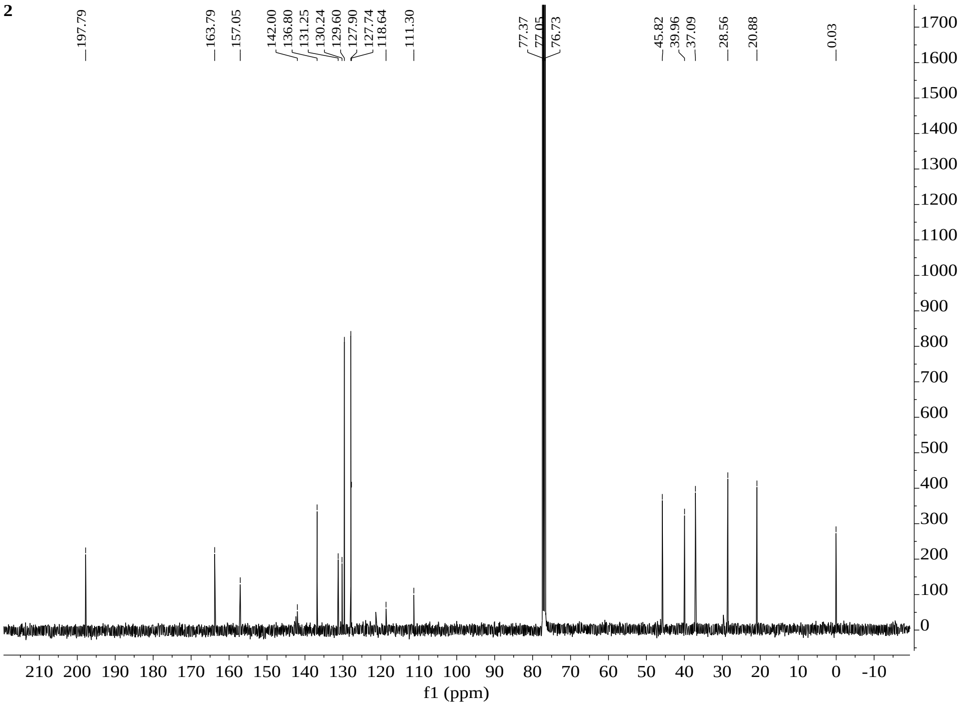

HRMS

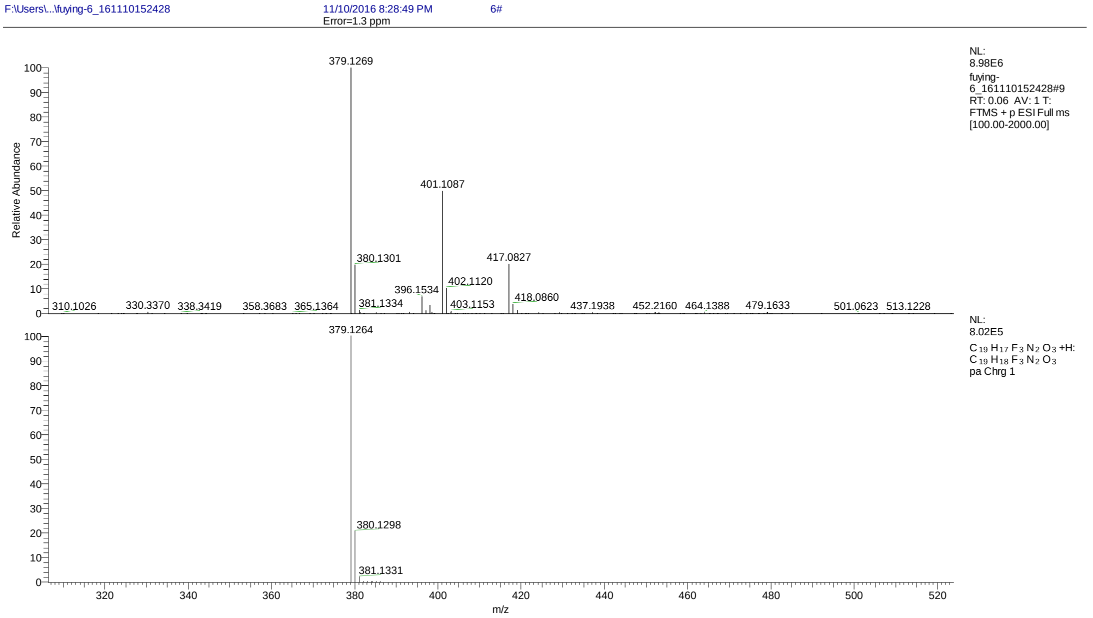

3-[(2-Trifluoromethyl-4-methyl)pyrazoloyloxy]-2-phenyl-5,5-dimethyl-2-cyclohexen-1-one (S28)

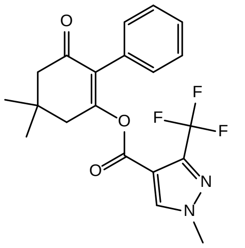

$C_{20}H_{19}F_3N_2O_3$

IR

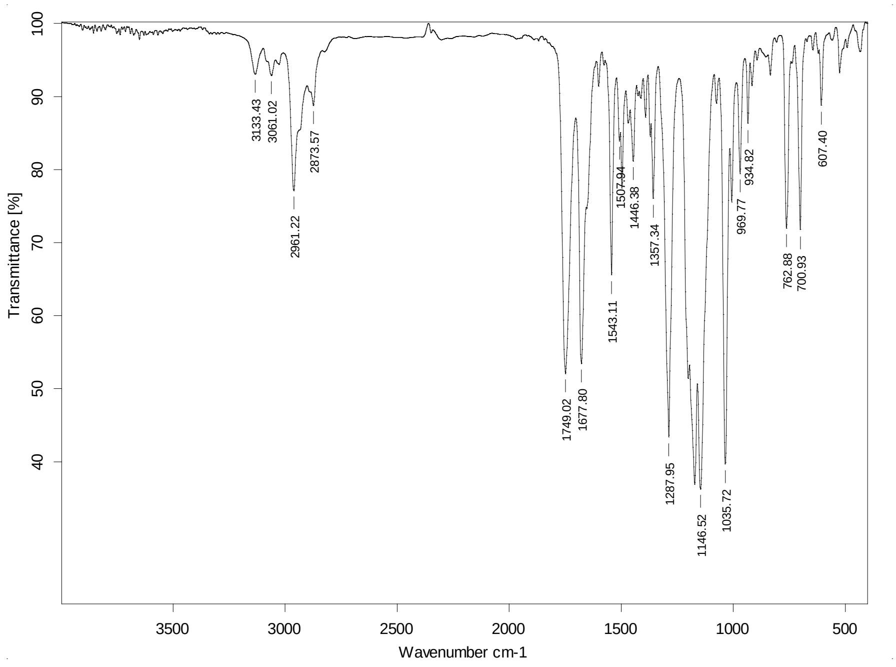

^{1}H NMR

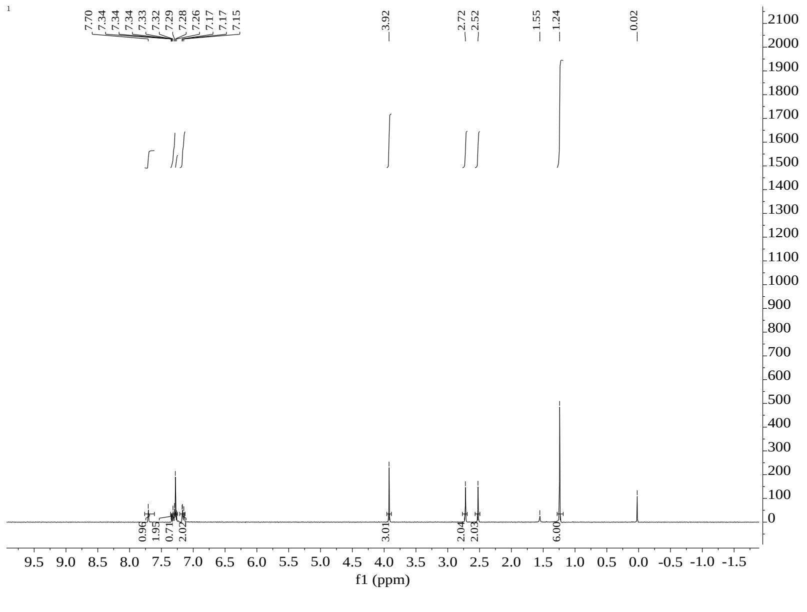

^{13}C NMR

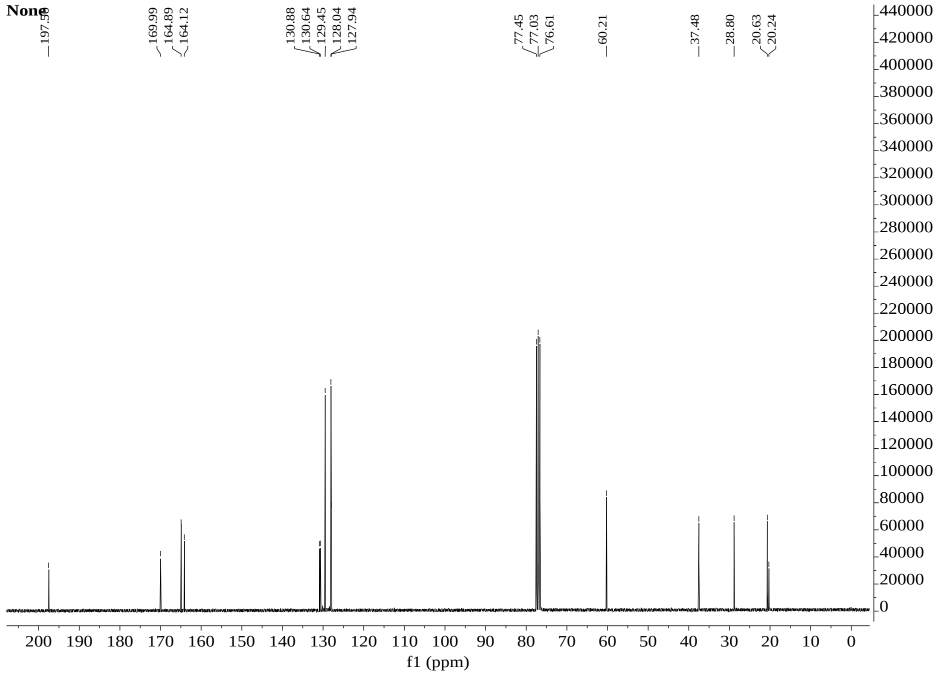
None
197.50
169.99
164.89
164.12
130.88
130.64
129.45
128.04
127.94
77.45
77.03
76.61
60.21
37.48
28.80
20.63
20.24
f1 (ppm)

HRMS

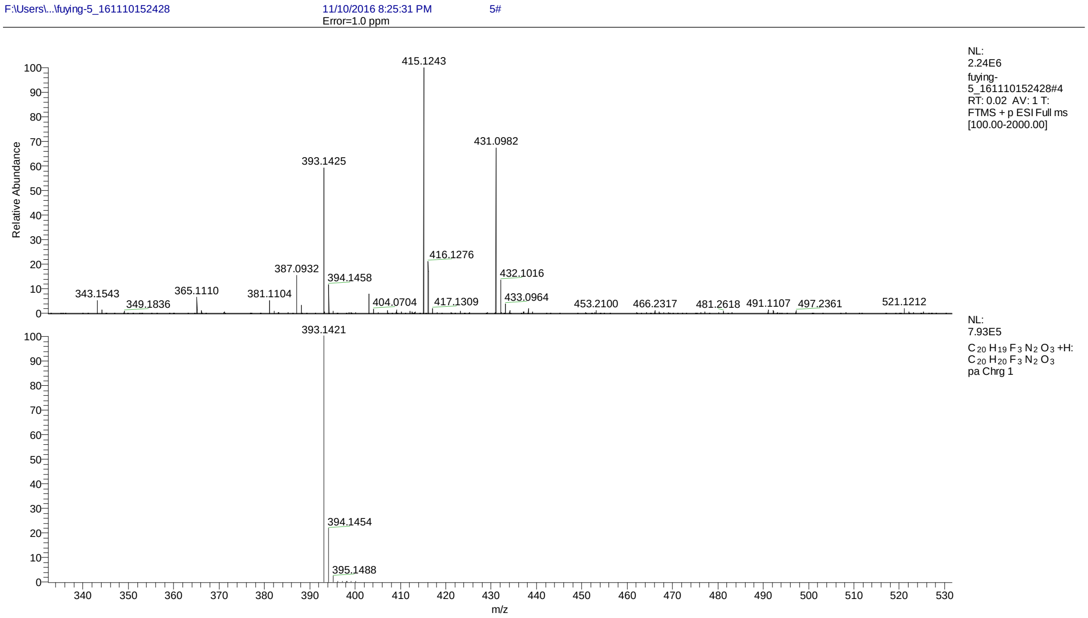
F:\Users\...\fuying-5_161110152428
11/10/2016 8:25:31 PM
5#
Error=1.0 ppm
NL: 2.24E6
fuying-5_161110152428#4 RT: 0.02 AV: 1 T: FTMS + p ESI Full ms [100.00-2000.00]
415.1243
431.0982
393.1425
416.1276
387.0932
394.1458
432.1016
343.1543
365.1110
381.1104
349.1836
404.0704
417.1309
433.0964
453.2100
466.2317
481.2618
491.1107
497.2361
521.1212
NL: 7.93E5
C 20 H 19 F 3 N 2 O 3 +H: C 20 H 20 F 3 N 2 O 3 pa Chrg 1
393.1421
394.1454
395.1488
Relative Abundance
m/z
